# Yoga is effective for treating chronic pain in veterans with Gulf War Illness at long-term follow-up

**DOI:** 10.1186/s12906-023-04145-y

**Published:** 2023-09-13

**Authors:** Santiago Allende, Danielle C. Mathersul, Jay R. Schulz-Heik, Timothy J. Avery, Louise Mahoney, Peter J. Bayley

**Affiliations:** 1 War Related Illness and Injury Study Center, VA Palo Alto Health Care System, Palo Alto, CA USA; 2 Department of Psychiatry and Behavioral Sciences, Stanford University School of Medicine, Stanford, CA USA; 3 School of Psychology, Murdoch University, Murdoch, WA 6150 Australia; 4 Centre for Molecular Medicine and Innovative Therapeutics, Health Futures Institute, Murdoch University, Murdoch, WA 6150 Australia; 5 Peninsula Behavioral Health, CA Palo Alto, 94306 USA; 6 Department of Veterans Affairs, Peninsula Vet Center, Menlo Park, CA 94025 United States of America

**Keywords:** Chronic pain, Fatigue, Gulf war illness, Long-term outcomes, Randomized clinical trial

## Abstract

**Background:**

Clinical Practice Guidelines for Gulf War Illness (GWI) recommend integrative health approaches such as yoga for relief from symptoms, yet little is known about the long-term efficacy of yoga in reducing symptoms of GWI. Here, we evaluated the long-term efficacy of yoga and cognitive-behavioral therapy (CBT) chronic pain treatment in a randomized controlled trial (RCT) of 75 Veterans (57 men, 42–71 ± 7.1 years of age) with Gulf War Illness (GWI).

**Methods:**

Participants received either 10 weeks of yoga or 10 weeks of CBT for chronic pain. The primary outcome measures were pain severity, and pain interference (Brief Pain Inventory-Short Form). The secondary outcome measures were fatigue, as indicated by a measure of functional exercise capacity (6-Minute Walk Test), depression, autonomic symptom severity, and quality of life. Piecewise linear mixed models were used to examine study hypotheses.

**Results:**

Compared to the CBT group, yoga was associated with greater reductions in pain severity during the 6-month follow-up period (group × time interaction: *b* = 0.036, *se* = 0.014, *p* = .011). Although we did not find between-group differences in the other primary or secondary outcome measures during follow-up (*p*’s > 0.05), exploratory analyses revealed within-group improvements in pain interference, total pain (an experimental outcome variable which combines pain severity and interference), and fatigue in the yoga group (*p*’s < 0.05) but not in the CBT group.

**Conclusions:**

This is the first study to report long-term follow-up results of yoga as a treatment for GWI. Our results suggest that yoga may offer long-term efficacy in reducing pain, which is a core symptom of GWI.

**Trial registration:**

Secondary analyses of ClinicalTrials.gov NCT02378025.

**Supplementary Information:**

The online version contains supplementary material available at 10.1186/s12906-023-04145-y.

## Background

Veterans from the First Gulf War Era (1990–1991) frequently present with a chronic multisymptom illness known as Gulf War Illness (GWI) that manifests as myriad symptoms, ranging from widespread chronic pain, headaches, sleep difficulties, fatigue, mood and cognitive impairments, to respiratory issues, gastrointestinal disturbances, and skin abnormalities [1–3]. Of the estimated 700,000 service members who served in Operation Desert Storm/Shield, approximately 203,000 (29%) to 420,000 (60%) met the CDC’s criteria for GWI [4].

There are currently few effective treatments for GWI. A 2019 review of GWI treatments [5], which found four treatments that had moved beyond Phase II trial, reported only modest efficacy of pharmacological [6], rehabilitation [7, 8], and psychological interventions [7]. For example, a randomized, double-blind, trial of doxycycline versus placebo for GWI failed to find significant between-group differences in measures of physical health, pain, fatigue, and cognitive function [6]. In a randomized controlled trial of aerobic exercise and cognitive behavioral therapy (CBT) for GWI, the adjusted odds ratio (OR_adj_) for physical function in the exercise alone group (primary outcome; vs. usual care) was 1.07, the OR_adj_ in the CBT alone group (vs. usual care) was 1.72, and the OR_adj_ in the exercise plus CBT group was 1.84 [7]. Secondary outcome measures of fatigue, distress, cognitive symptoms, and mental health functioning significantly improved in the exercise group and in the exercise plus CBT group but only cognitive symptoms and mental health functioning improved in the CBT alone group. Importantly, significant reductions in pain were not found in any of the treatment groups.

Similar conclusions regarding available treatments for GWI were reached by the VA/DoD Clinical Practice Guidelines for the management of chronic multisymptom illness [9] which recommends an integrative health approach to treating GWI including yoga, meditation or physical exercise. In particular, yoga, which may partially exert its salubrious effects through enhanced interoceptive awareness [10], has been shown to reduce pain in other pain-centric conditions, such as fibromyalgia [11], rheumatic diseases [12], and multiple sclerosis [13]. Therefore, further long-term studies are needed to establish yoga as an effective treatment for GWI.

We recently published an RCT examining yoga as a treatment for GWI which to our knowledge was the first such study [14]. Cognitive behavioral therapy was used as the active control condition because it is the only psychological treatment that has been tested for GWI [7], it is the only psychological treatment that is strongly recommended by the VA/DoD Clinical Practice Guidelines for the management of chronic multisymptom illness [9], and because it has not shown efficacy in treating pain in GWI [7]. Our study compared a 10-week yoga program to CBT (ClinicalTrials.gov NCT02378025; *N* = 75 randomized). At end-of-treatment, the primary outcomes of pain severity and interference (Brief Pain Inventory- Short Form) improved in the yoga condition but not in the CBT condition. While the differences between groups were not statistically significant, the difference in an a-priori-defined experimental outcome variable which combined these two variables into a “total pain” variable was statistically significant. Furthermore, fatigue was reduced significantly more in the yoga group than in the CBT group as indicated by a measure of functional exercise capacity (6-min walk test). While these effects of yoga on core symptoms of GWI are promising, it is important to establish the durability of the treatment effect. Here, we present findings from a long-term follow-up analysis that sought to determine whether these treatment differences and gains were sustained during the 6-month follow-up period.

## Methods

### Procedure

The parent trial randomized Veterans with GWI into one of two treatment arms: (a) 10 weeks of manualized CBT for pain or (b) 10 weeks of manualized yoga for pain (see [14] for details). The study was conducted from June of 2015 to March of 2018 at the War Related Illness & Injury Study Center at the Veterans Affairs Palo Alto Health Care System in accordance with the latest version of the Declaration of Helsinki. The study was approved by the Stanford University Institutional Review Board (ClinicalTrials.gov NCT02378025). In the parent trial, participants completed the Brief Pain Inventory-Short Form (BPI-SF; primary outcome) at baseline, weeks 2, 4, 6, and 8, and at end-of-treatment (EOT; within one week of session 10). The secondary outcome measures were completed at baseline and at EOT and included fatigue, health-related quality of life, depression, and autonomic symptom severity. In the present study, the post-treatment follow-up assessments for the BPI-SF were made at 2 months (8 weeks), 4 months (16 weeks), and 6 months (24 weeks). Secondary outcome measures were collected at 6 months (24 weeks) post-treatment.

### Participants

We used the Fukuda [15] criteria to diagnose GWI, which does not require deployment. To be included in the study, we required participants to be (a) in the US armed forces between 1990 and 1991, (b) score in the moderate to severe chronic pain range on the Short-Form McGill Pain Questionnaire [16], and (c) diagnosed with GWI per the Fukuda [15] criteria. Study exclusion criteria were (a) inability to stand or walk, (b) concurrent enrollment in a separate clinical trial, (c) considerable difficulty traveling to the study site, and (d) suicidality (intent or plan). All participants provided written informed consent. Demographic information is presented in Table 1 (*n* = 75, age range 42–71 years, mean 53, *SD* = 7.1; 24% female). Veterans were recruited using flyers, newspaper advertisements, and referrals from healthcare providers.

**Table 1:** Demographics and baseline clinical characteristics

Characteristics	All Participants		CBT		Yoga	
	n / mean	% / SD	n / mean	% / SD	n / mean	% / SD
N	75	-	36	-	39	-
Age	53.0	7.1	52.0	6.8	53.8	7.2
Male	57	76%	26	72%	31	79%
Race						
Caucasian	36	48%	20	56%	16	41%
African-American	11	15%	4	11%	7	18%
Other	10	13%	5	13%	5	13%
Asian American	9	12%	3	8%	6	15%
Native American	6	8%	3	8%	s3	8%
Hispanic ethnicity	15	24%	7	23%	8	25%
BMI	31.6	5.6	31.3	5.5	31.8	5.7
Married	36	48%	14	40%	22	58%
Education (years)	15.0	2.2	15.1	2.2	14.8	2.3
Employed	30	42%	14	40%	16	43%
Disabled^a^	28	38%	14	37%	14	38%
McGill Short Form						
Sensory pain	15.0	7.2	14.2	7.5	15.7	6.9
Affective pain	4.5	3.6	4.3	3.7	4.8	3.5
Pain right now^b^	5.2	2.5	5.0	2.5	5.4	2.4
GWI Symptoms^c^						
Fatigue (y/n)	43	57%	19	54%	24	62%
Cognitive (# of sx)^d^	3.5	2.7	3.3	2.9	3.7	2.6
Any cognitive sx (y/n)	56	75%	25	71%	31	79%
POMS	26.8	29.9	19.6	23.6	33.1	33.5
Prior yoga practice (y/n)	19	25%	11	31%	8	21%

*CBT* Cognitive behavior therapy, *SD* Standard deviation, *BMI* Body mass index, *GWI* Gulf War illness; sx = symptoms, *POMS* Profile of mood states

^a^Disabled = receiving disability benefits, irrespective of whether also employed, enrolled in school, etc

^b^Visual analog scale subsequently translated to a 0 – 10 scale

^c^Self-reported symptoms experienced subsequent to 1991 Gulf War and during three months prior to study

^d^Out of a total of 7 cognitive symptoms reflecting Fukuda cognitive symptom criteria (concentration, memory, word finding difficulties, anxiety, depression, irritability, feeling moody)

### Interventions

In both treatment arms, participants met once per week for 10 weeks for 60 min in groups of ≤ 10 individuals (mean = 5.4). A CBT protocol to improve physical function in Veterans with GWI was adapted for the present study by reducing the 12 sessions to 10 sessions by eliminating the problem-solving module and the memory enhancement module [7]. The CBT modules included psychoeducation on the CBT model of pain, pacing of activities, pleasant events scheduling, identifying and challenging cognitive distortions, sleep hygiene, relaxation and stress management, interpersonal skills, and problem solving. Each session was co-led by a primary licensed psychologist and an assistant doctoral student. Group facilitators were required to have prior training and experience in delivering CBT interventions and received specific training in the GWI pain protocol from a behavioral medicine clinical psychologist with extensive CBT training.

The 10-week yoga protocol [17] incorporated breath-linked postures from the Krishnamacharya/Desikachar lineage of Hatha yoga with therapeutic adaptations based on the yoga teachers’ experience with Gulf War and Vietnam era Veterans in the VA (see Table 3 in Appendix 1 for protocol details). The classes also emphasized awareness of breath and postural adaptations for use in daily activities. Each session was facilitated by one primary yoga instructor and either one or two additional assistant yoga instructors. All yoga instructors held at least a 200 h yoga teacher certification and had previously taught yoga to Veterans. In addition to modifiable and adaptable postures, sessions included controlled breathing, hand gestures, vocalization, and meditation. Apart from the 60-minutes of weekly in-class yoga practice, participants were also asked to use a homework handout with written instructions and pictures of postures to practice yoga at home for 15–30 min on an additional 5 days. Participants could also use one of the War Related Illness and Injury Study Center yoga classes at the Palo Alto VA Medical Center to substitute for homework practice. The study protocol was developed by study co-authors LM (certified yoga therapist) and a study physiatrist who consulted rehabilitation research and yoga texts.

### Measures

#### Primary outcome

The Brief Pain Inventory-Short Form (BPI-SF) was the primary outcome and was administered 2,4, and 6 months post-treatment [18]. The measure includes two subscales; pain severity and pain interference, designed to quantify the severity of pain and the degree to which pain adversely impacts functioning. Pain severity is computed as the mean of four items rated on an 11-point Likert-type scale: (a) pain at its worst in the last 24 h, (b) pain at its least in the last 24 h, (c) pain on average, and (d) pain right now. Pain interference is computed as the average of nine items related to activities of daily living, including general activity, walking ability, mood, enjoyment of life, ability to concentrate, sleep, appetite, relations with others, and normal work functioning, each rated on an 11-point Likert-type scale. Consistent with the parent study [14], we also derived a BPI-SF “total score” by computing the mean of the pain severity and pain interference items.

#### Secondary outcomes

##### Fatigue

In accordance with standard guidelines [19], we used the 6-Minute Walk Test of functional exercise capacity to assess fatigue [20]. This measured how far participants could walk in 6 min around an indoor 32 m flat-surfaced track [19, 20].

##### Quality of life

The Short Form Health Survey-36 (SF-36) was used to measure health-related quality of life [21]. The SF-36 comprises eight scales that assess physical functioning, physical health related role limitations, emotional health related role limitations, energy/fatigue, emotional wellbeing, social functioning, pain, and general health.

##### Depression

Depressive symptomatology was quantified using the Hamilton Depression Rating Scale-28 (HAM-D-28), an interviewer-administered questionnaire [22].

##### Autonomic symptom severity

Autonomic symptom severity was assessed using the 31-item self-report Composite Autonomic Symptom Scale (COMPASS-31) [23]. This measure assesses the frequency and severity of symptoms in a number of domains, including orthostatic intolerance, vasomotor, secretomotor, gastrointestinal, constipation, bladder, and pupillomotor. An example item from the gastrointestinal domain is, “In the past year, have you had a cramping or colicky abdominal pain?” and an example item from the pupillomotor domain is, “In the past year, without sunglasses or tinted glasses, has bright light bothered your eyes?”

### Data analytic plan

Following the intention-to-treat principle, we used piecewise linear mixed models to examine long-term differences between yoga and CBT on BPI-SF pain severity, BPI-SF pain interference, and BPI-SF total score. To adjust for baseline to end-of-treatment effects, we coded two segments for time with a knot at 10 weeks. As a consequence of maximum likelihood estimation, linear mixed models are found to perform better than ordinary least squares approaches when data are missing completely at random or at random conditional on observed data [24–26]. In addition, due to their flexibility in partitioning variance, mixed models can better handle correlated residuals in longitudinal data [24–26].

Software packages from the R statistical computing environment were used to analyze the data. We used the tidyverse set of R packages to clean and structure the data, the nlme R package to estimate the mixed models [27], and the ggeffects R package to produce the figures. We determined the best fitting models by comparing plots of model-estimated trajectories to mean-based trajectories, the likelihood ratio test, and model assumption plots at levels 1 and 2 (see Table 2 for model specifications). In addition to adjusting for baseline to end-of-treatment (EOT) by group interactions, we adjusted for baseline depression, a well-established prognostic covariate of chronic pain [28], to reduce baseline differences in the outcome variables [29]. The final models were estimated with restricted maximum-likelihood estimation. We also performed a sensitivity analysis by examining a three-way interaction between EOT to 6 months follow-up (FU), group, and a binary variable denoting whether participants were missing any of the BPI-SF follow-up time period assessments [30, 31]. For significant interaction terms, the emmeans R package, which makes use of the delta method to compute standard errors, was used to derive 6-month post-treatment estimates averaged over the levels of the binary missing data variable [30–32] (see Supplementary Material for the R Notebooks). The data from the present analyses are available upon reasonable request to the authors.

**Table 2:** Longitudinal outcomes in pain severity, pain interfence and pain yotal: yoga versus CBT

BPI-SF Pain Severity						BPI-SF Pain Interference					BPI-SF Pain Total Score				
*Predictors*	*b*	*se*	*CI*	*t*	*p*	*b*	*se*	*CI*	*t*	*p*	*b*	*se*	*CI*	*t*	*p*
Baseline HAM-D	0.11	0.03	0.06 – 0.17	4.20	<.001***	0.18	0.03	0.12 – 0.24	6.17	<.001***	0.16	0.03	0.11 – 0.22	6.14	<.001***
Baseline to EOT	-0.09	0.03	-0.15 – -0.03	-3.07	.002**	-0.15	0.03	-0.22 – -0.08	-4.38	<.001***	-0.13	0.03	-0.19 – -0.07	-4.33	<.001***
Group [Yoga]	-0.08	0.41	-0.90 – 0.75	-0.18	.855	-0.28	0.46	-1.19 – 0.63	-0.62	.540	-0.24	0.41	-1.06 – 0.58	-0.58	.565
EOT to FU [Weeks 10 to 34]	-0.03	0.01	-0.05 – -0.01	-3.29	.001**	-0.03	0.01	-0.05 – -0.00	-2.34	.020*	-0.03	0.01	-0.05 – -0.01	-2.87	.004**
Baseline to EOT × Group [Yoga]	0.06	0.05	-0.04 – 0.14	1.24	.215	0.11	0.06	-0.00 – 0.22	1.91	.057	0.09	0.05	-0.00 – 0.19	1.88	.061
EOT to FU × Group [Yoga]	0.04	0.01	0.01 – 0.06	2.55	.011*	0.02	0.02	-0.01 – 0.06	1.24	.216	0.03	0.02	-0.00 – 0.06	1.75	.082

*HAM-D* Hamilton Depression Rating Scale; *EOT* End-of-Treatment; [reference group]; *FU* Follow-up time period; *ID* participant identifier; Random effects of models are shown in Table 2 in Appendix 1; *p<.05, **p<.01, ***p<.001

To derive a Cohen’s *d*effect size at each follow-up time period, we divided the difference in treatment means at each time point by the square root of the sum of variances of the random effects (i.e., intercept, slope, and residual variance components) [33]. Consistent with the parent trial, we quantified clinically significant improvement as a 15% decrease and as a 1-point decrease from baseline to 6 months post-treatment for pain severity and pain interference, respectively [34]. Clinically significant improvement on the 6-Minute Walk Test was defined as a 30.5 m change in walking distance from baseline to 6 months post-treatment [35]. Due to small sample sizes, Fisher’s exact test was used to examine between-group clinically significant improvements only among participants who completed the BPI at 6 months.

The models for fatigue, health-related quality of life, depression, and autonomic symptom severity were random intercept models with fixed effects for baseline to EOT, EOT to FU, group, and interaction terms between each of the time variables and group. Each model was adjusted for baseline average pain using the BPI-SF total score. Examination of assumption plots for each of the models showed adequate normality, independence, homoscedasticity, and linearity. Missing data per treatment arm and follow-up time period are shown in Table 3.

## Results

### Primary outcome

**Fig. 1:**
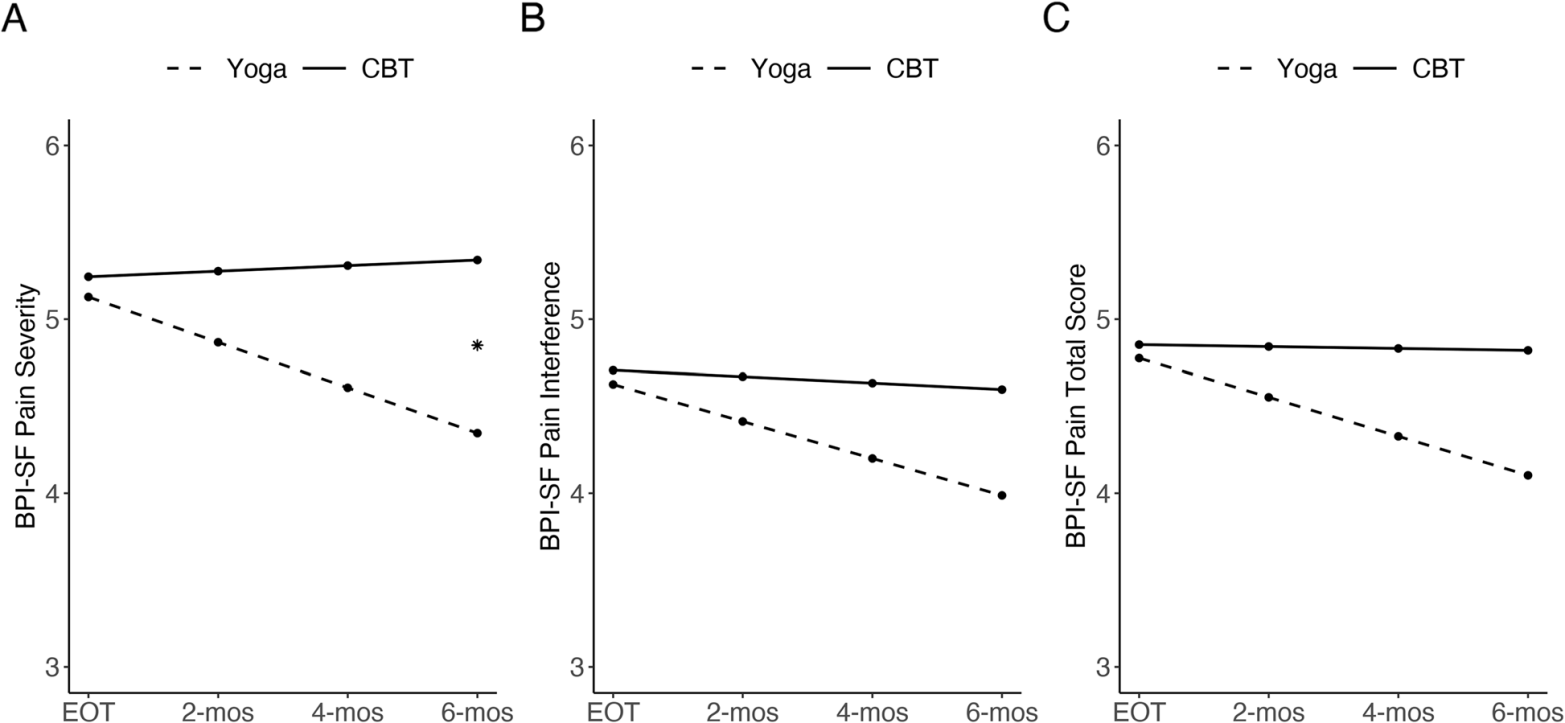
Model-derived trajectories for pain from end-of-treatment (EOT; week 10) to 6 months (mos) post-treatment for yoga and CBT; **A** BPI-SF Pain Severity; **B** BPI-SF Pain Interference; **C** BPI-SF Pain Total; y-axes do not start at 0; *p < .05

The model for BPI-SF pain severity showed a significant interaction between EOT to FU and treatment group, with a greater reduction in BPI-SF pain severity in the yoga group than in the CBT group (see Table 2; Fig. 1). The Cohen’s *d* effect size estimates at EOT, 2, 4, and 6 months post-treatment were 0.26, 0.04, 0.27, and 0.18, respectively (see Table 3 for other follow-up timepoints; *n* [yoga EOT] = 32; *n* [CBT EOT] = 19; *n* [yoga 6-month FU] = 29; *n* [CBT 6-month FU] = 18). Although our findings did not demonstrate between-group longitudinal differences in pain interference, the model demonstrated a significant within-group reduction in BPI-SF pain interference during the follow-up period for the yoga group (see Table 2; Fig. 1). In a secondary analysis, a separate model for the CBT group failed to show a reduction in BPI-SF pain interference during the follow-up time period (*b* = -0.004, *se* = 0.01, *t*(116) = -0.35, *p* = .724). Similarly, while the interaction term was not significant for BPI-SF total score, there was a statistically significant within-group effect for yoga during the follow-up period (see Table 2; Fig. 1). Using a separate model in a secondary analysis, we did not find within-group reductions in BPI-SF total score for the CBT group during the follow-up period (*b* = -0.001, *se* = 0.01, *t*(116) = -0.13, *p* = .899).

**Table 3:** Effect size estimates by group (Yoga vs. CBT) and follow-up time period for primary outcome measures

Treatment	Follow-up	Pain Severity Mean	Pain Severity Cohen’s *d*	Pain Interference Mean	Pain Interference Cohen’s *d*	Pain Total Mean	Pain Total Cohen’s *d*
Yoga	EOT	4.67 ±2.44 (*n* = 32)	0.26	3.65 ±2.17 (*n* = 31)	0.50	4.00 ±2.08 (*n* = 31)	0.47
	2m FU	5.19 ±1.89 (*n* = 24)	0.04	4.65 ±2.47 (*n* = 23)	0.36	4.84 ±2.19 (*n* = 23)	0.25
	4m FU	4.70 ±2.34 (*n* = 23)	0.27	4.37 ±2.44 (*n* = 23)	-0.00	4.47 ±2.24 (*n* = 23)	0.06
	6m FU	4.91 ±1.93 (*n* = 29)	0.18	4.60 ±2.49 (*n* = 27)	-0.20	4.70 ±2.17 (*n* = 27)	-0.11
CBT	EOT	5.20 ±2.60 (*n* = 19)	*0.26*	4.65 ±2.60 (*n* = 16)	*0.50*	4.95 ±2.35 (*n* = 16)	*0.47*
	2m FU	5.27 ±2.43 (*n* = 15)	*0.04*	5.36 ±3.15 (*n* = 15)	*0.36*	5.33 ±2.87 (*n* = 15)	*0.25*
	4m FU	5.23 ±2.55 (*n* = 15)	*0.27*	4.37 ±2.78 (*n* = 14)	*-0.00*	4.59 ±2.68 (*n* = 14)	*0.06*
	6m FU	5.26 ±2.67 (*n* = 18)	*0.18*	4.20 ±2.71 (*n* = 17)	*-0.20*	4.48 ±2.64 (*n* = 17)	*-0.11*

*Note*: *EOT* End-of-Treatment; *m* month; *FU* Follow-up time point; Cohen’s *d* estimated as the difference in treatment means at each follow-up time point divided by square root of the sum of variances of random effects

Fisher’s exact tests to assess between-group differences in clinical improvement for both BPI-SF pain severity and BPI-SF pain interference were not significant, (*OR* = 0.48, *CI*[0.11,1.94], *p* = .356) and (*OR* = 0.34, *CI*[0.06,1.49], *p* = .125), respectively. Of the 29 participants in the yoga group who completed the pain severity measure at 6 months post-intervention, 13 (45%) met criteria for clinical improvement. For BPI-SF pain interference, 48% (13/27) of the participants in the yoga group met criteria for clinical improvement. In the CBT group, 5 of the 18 participants (28%) who completed the BPI-SF pain severity measure at 6 months post-treatment met criteria for clinical improvement, while 24% (4/17) met criteria for clinical improvement in pain interference.

### Secondary outcomes

Our results did not show evidence of longitudinal group differences in walking distance, as measured by the 6-Minute Walk Test, from EOT to FU (*b* = -1.249, *se* = 0.821, *t*(83) = -1.52, *p* = .132, *d* = -3.05). However, the model showed a significant increase in walking distance from EOT to FU in the yoga group (*b* = 1.164, *se* = 0.508, *t*(83) = 2.29, *p* = .024; see Fig. 1). A secondary within-group analysis did not demonstrate an increase in walking distance in the CBT group (*b* = -0.088, *se* = 0.503, *t* [31] = -0.17, *p* = .863). Of the 24 participants in the yoga group who completed baseline and follow-up walking distance tests, 11 (46%) met criteria for clinical improvement, while 33% (5/15) in the CBT group met criteria for clinical improvement. However, Fisher’s exact test for clinical improvement was not significant (*OR* = 0.60, *CI*[0.12,2.69], *p* = .517). We did not find significance for depression (*b* = 0.127, *p* = .064, *d* = 0.04), autonomic symptom severity (*b* = 0.061, *p* = .708, *d* = -0.22), or quality of life (*b* = 0.193, *p* = .249, *d* = 0.26). Between-group effect size estimates, means, and standard deviations for each of the secondary measures are shown in Appendix 1.

### Sensitivity analyses

The models with pain severity and pain interference entered as dependent variables did not show significant three-way interactions (BPI-SF pain severity: *b* = 0.056, *t*(306) = 1.912, *p* = .057; BPI-SF pain interference: *b* = 0.063, *t*(294) = 1.706, *p* = .089). However, our results revealed a significant three-way interaction for BPI-SF total score, with the score total decreasing less for CBT participants who were missing follow-up data (*b* = 0.067, *se* = 0.031, *t*(294) = 2.12, *p* = .035). The between-group difference in BPI-SF total score at 6 months post-treatment, averaged over the levels of the binary missing data variable, was − 1.27 (*se* = 0.582, *p* = .033). The estimated marginal mean for BPI-SF total score at 6 months post-treatment in the yoga group was 3.91 (*se* = 0.373, *CI*[3.16,4.65]), while the estimated marginal mean for the CBT group was 5.18 (*se* = 0.441, *CI*[4.30,6.06].

## Discussion

The aim of this study was to examine the long-term efficacy of yoga (vs. CBT) on symptoms of GWI, including pain, walking distance/fatigue, depression, autonomic symptom severity, and quality of life. During the 6-month follow-up period, our results from the BPI-SF showed evidence of reduced pain severity in the yoga group compared to the CBT group, with almost twice as many veterans meeting criteria for clinical improvement in pain severity in the yoga group than the CBT group at 6 months post-intervention (45% vs. 28%). We did not find between-group longitudinal differences in BPI-SF pain interference, BPI-SF total score, walking distance, depression, autonomic symptom severity, or quality of life. However, secondary within-group analyses showed long-term improvements in BPI-SF pain interference, BPI-SF total score, and walking distance in the yoga group but not in the CBT group. Our results failed to demonstrate long-term within-group treatment gains for CBT or yoga on measures of depression, autonomic symptom severity, or quality of life.

To our knowledge, this is the first RCT to report long-term benefits of yoga for GWI. A few other types of CIH interventions have shown similar effects. In one such trial, mindfulness-based stress reduction (vs. treatment as usual) was associated with greater reductions in pain, fatigue, depression, and cognitive errors in everyday tasks at 6 months post-intervention [36]. In a 3-month follow-up study, a sleep-focused mind-body intervention for symptoms of GWI showed sustained improvements in sleep, PTSD symptomatology, fatigue, and depression [37]. Importantly, though, a behavioral sleep intervention (CBT-I) without CIH components also demonstrated sustained 6-month reductions in pain interference, aberrant sleep, fatigue, cognitive failures, anxiety, and depression [38]. This promising result may be related to sleep as a core regulator of homeostatic functions [39]. Given the few trials reporting long-term outcomes for GWI, our interpretations on the use of CIH approaches in Veterans with GWI are preliminary. Nevertheless, our findings are consistent with the current VA/DoD Clinical Practice Guidelines for the management of chronic multisymptom illness which recommends treating GWI with integrative health modalities, including yoga [9].

The pattern of results across multiple CIH studies is indicative of multisystemic healing and may be linked to the theoretical underpinnings of yoga and related CIH interventions. A core assumption underlying CIH approaches is the construct of holistic healing, which emphasizes the use of feedback processes between the mind and body to achieve greater homeostatic regulation [40, 41]. For example, a change in the quality of the breath is thought to impact the experience of thoughts in the mind, which interface with other physiological processes and attitudes towards life. A mechanism by which yoga and related holistic health approaches treat pain may be through enhanced interoceptive awareness, whereby practice-related improvements in noticing, distracting, worrying, attention regulation, body-listening, and trusting function to increase wellbeing and reduce pain and its sequelae [10]. Relatedly, increased proprioceptive awareness may also mediate the relationship between yoga practice and reduced pain by way of reducing muscle tension and improving posture [42]. Insofar that GWI is a multisystem illness associated with psychoneuroimmunologic dysregulation [43, 44], mind-body approaches that emphasize holistic healing may be uniquely poised to alleviate symptoms of GWI.

In our study, the yoga protocol emphasized the connection between movement and the breath, which may have increased relaxation and resulted in decreased chronic pain and fatigue, both of which are common in multisymptom illnesses [45, 40]. We also encouraged participants to attend to and adjust their posture throughout the day to improve circulation and decrease pain. The prior mindfulness-based stress reduction and mind-body sleep studies may have also demonstrated multisystemic improvements by way of targeting the breath/body connection [36, 37, 40]. While it was plausible that aerobic exercise, as a potent biopsychosocial intervention, would alleviate symptoms of GWI, prior findings have shown aerobic exercise has only modest efficacy in GWI [7]. This contradictory outcome may be due to less emphasis on the breath/body connection in exercise interventions. Future studies may benefit from integrating breath awareness practices into exercise routines to bolster the multisystemic effects of exercise and reduce GWI symptomatology.

Interestingly, consistent with prior results showing limited efficacy of CBT for GWI [7], our study also failed to show a long-term effect of CBT on symptoms of GWI. This observation may be understood by considering the distinction between bottom-up and top-down self-regulatory approaches to health. Bottom-up regulatory practices are characterized by a focus on the breath/body connection (e.g., yoga and meditation) whereas top-down regulatory practices are characterized by a focus on thoughts to affect emotions and bodily states (e.g., CBT) [46, 47] Compared to embodied yoga and meditation practices, top-down CBT techniques, such as cognitive reappraisal (i.e., challenging the initial reactive interpretation of distressing stimuli), possibly instill a sense of fighting or resisting the pain [48]. This could result in a dampened multisystemic effect of CBT on symptoms of GWI. On the other hand, the slowing of the breath in yoga and other contemplative practices is proposed to strengthen vagal activity, increase parasympathetic dominance, and cultivate a sense of safety in the mind/body [49]. This bottom-up generated relaxation response, echoing the construct of holistic healing, may function to improve the broad-ranging symptoms that characterize GWI by increasing compassionate acceptance of the pain [45].

While intriguing, our interpretations must be contextualized within the limitations of the present study. For example, there was greater dropout in the CBT group than in the yoga group. Even with the use of maximum likelihood estimation, the estimates may have been biased by differential attrition. Moreover, the clinically significant improvement and effect size statistics may have been biased by attrition. Due to the small sample size, the three-way interaction in the sensitivity analysis was likely underpowered. Although it revealed greater reductions in BPI-SF total score in the yoga group, as a conservative approach, we did not report this as a meaningful finding. To increase power and rectify common issues related sensitivity analyses, studies with larger sample sizes will be needed [30]. On the basis of our results, we encourage future GWI studies to consider the long-term benefits of treatment, and to examine if treatment gains are dependent on continued practice.

## Conclusions

This is the first long-term follow-up study of yoga for treating the symptoms of GWI. Neither yoga nor CBT showed long-term improvements in depression, autonomic symptom severity, or quality of life. However, our results suggest that yoga, but not CBT, is associated with long-term (6-month) reductions in pain among Veterans living with GWI.

### Supplementary Information

**Additional file 1.**

**Additional file 2.**

## Data Availability

The datasets used and/or analyzed during the current study are available from the corresponding author on reasonable request.

## Abbreviations

GWI: Gulf War Illness
CBT: Cognitive-behavioral therapy
BPI-SF: Brief Pain Inventory-Short Form)
SF-36: Short Form Health Survey-36
HAM-D-28: Hamilton Depression Rating Scale-28
COMPASS-31: Composite Autonomic Symptom Scale-31
EOT: End-of-Treatment
FU: Follow-up time point
m: month
ID: Participant identifier
ICC: Intraclass correlation coefficient
