# Supplementary material for: Yoga is effective for treating chronic pain in veterans with Gulf War Illness at long-term follow-up
Source: BMC Complement Med Ther. 2023 Sep 13;23:319. doi: 10.1186/s12906-023-04145-y (PMC10498617; doi:10.1186/s12906-023-04145-y)
Supplement: Supplementary file 1 — Additional file 1. [file 12906_2023_4145_MOESM1_ESM.pdf]

# APPENDIX 1

Table 1

*Effect size estimates by group (Yoga vs. CBT) and follow-up time period for secondary outcome measures*

| Treatment | Follow-up | Walk Distance Mean | Walk Distance Cohen's *d* | Depression Mean | Depression Cohen's *d* | Autonomic Symptom Severity Mean | Autonomic Symptom Severity Cohen's *d* | Quality of Life Mean | Quality of Life Cohen's *d* |
|---|---|---|---|---|---|---|---|---|---|
| Yoga | EOT | 492.49 ±121.53 (*n* = 30) | -1.26 | 11.25 ±8.04 (*n* = 32) | -0.05 | 21.46 ±17.00 (*n* = 30) | 0.07 | 51.56 ±21.34 (*n* = 32) | -0.25 |
| | 6m FU | 492.27 ±119.50 (*n* = 27) | -3.05 | 10.89 ±7.57 (*n* = 28) | 0.04 | 24.02 ±18.38 (*n* = 28) | -0.22 | 47.50 ±21.37 (*n* = 28) | 0.26 |
| CBT | EOT | 473.98 ±113.85 (*n* = 18) | -1.26 | 10.47 ±7.31 (*n* = 19) | -0.05 | 22.50 ±15.50 (*n* = 18) | 0.07 | 47.92 ±22.30 (*n* = 18) | -0.25 |
| | 6m FU | 447.35 ±98.89 (*n* = 15) | -3.05 | 11.53 ±8.35 (*n* = 17) | 0.04 | 20.79 ±17.09 (*n* = 17) | -0.22 | 51.39 ±22.74 (*n* = 18) | 0.26 |

*Note.* EOT = End-of-Treatment; m = month; FU = Follow-up time point; Cohen's *d* estimated as the difference in treatment means at each follow-up time point divided by square root of the sum of variances of random effects

Table 2

*Random effects of longitudinal outcomes in Pain Severity, Pain Interfence, and Pain Total: Yoga Versus CBT*

| | **BPI-SF Pain Severity** | **BPI-SF Pain Interference** | **BPI-SF Pain Total Score** |
|---|---|---|---|
| $\sigma^2$ | 0.96 | 1.49 | 1.10 |
| $\tau_{00}$ | 2.29 ID | 2.52 ID | 2.11 ID |
| $\tau_{11}$ | 0.01 ID baseline to EOT | 0.01 ID baseline to EOT | 0.01 ID baseline to EOT |
| $\rho_{01}$ | 0.34 ID | 0.08 ID | 0.17 ID |
| ICC | 0.75 | 0.66 | 0.70 |
| N | 71 ID | 70 ID | 70 ID |
| Observations | 383 | 370 | 370 |

Table 3

Yoga protocol targets, practices, and weekly class themes

<table>
<tr><th>Target</th><th>Yoga Tool</th><th>Weekly Class Themes</th></tr>
<tr><td>Breath and sensation awareness</td><td>Natural breath, body scan</td><td rowspan="9">1. Seated and standing posture and use of yoga props for posture adjustment<br>2. Cultivating compassion for yourself first and then others<br>3. Focus on maintaining Ujayii breathing during class<br>4. Move slowly and find comfort in each yoga posture and breath practice<br>5. Repeat of week 4<br>6. Be mindful of what is “right” with and for us in this moment<br>7. Be mindful of how pain has become familiar – like a protective shell – but we no longer need it and can let it go<br>8. Focus on slow gentle breath throughout the practice<br>9. Finding balance and what is “just right” for us in today’s practice as in the Goldilocks tale<br>10. Last day – reminder to bring yoga into your day-to-day life by paying attention to your body, mind, and spirit and be content with just enough – not too much, not too little.</td></tr>
<tr><td>Balancing, calming, activating breath practices</td><td>Sama Vritti, Surya Bhyedan, Nadi Shodhana, Ujayii, Simha, Brahmari, Kapalabhati, Breath of Joy</td></tr>
<tr><td>Posture and alignment</td><td>Seated or standing Tadasana</td></tr>
<tr><td>Focused breath with movement</td><td>Seated or standing adaptive Sun Salutations, flexion & extension of the spine (cat/cow)</td></tr>
<tr><td>Musculoskeletal strength, balance and flexibility AND moving with the pace of the breath</td><td>Virabhadrasana I & II, Utthita Tadasana, Utkatasana, Vatayanasana, Urdhva Hastasana with lateral bend, Prasarita Padottanasana, Parvostanasana, Vrikshasana, modified Supta Padangusthasana, Ardha Matsyendrasana, Vasisthasana, modified Salabhasana, Balasana, Bent Knee/Tabletop internal oblique strengthen, modified Paripurna Navasana, Setu Bandha Sarvangasana</td></tr>
<tr><td>Gastrointestinal Health</td><td>Apanasana, Bananasana, Supta Baddha Konasana</td></tr>
<tr><td>Calming</td><td>Makarasana, Jathara Parivartanasana, Viparita Karani, Savasana</td></tr>
<tr><td>Hand Gestures</td><td>Anjali, Surya, Mrigi Mudras</td></tr>
</table>
