# Supplementary material for: Yoga is effective for treating chronic pain in veterans with Gulf War Illness at long-term follow-up
Source: BMC Complement Med Ther. 2023 Sep 13;23:319. doi: 10.1186/s12906-023-04145-y (PMC10498617; doi:10.1186/s12906-023-04145-y)

Primary Follow-up Analyses Yoga GWI

Load Libraries

```
library(tidyverse)
library(nlme)
library(sjPlot)
library("PsychLab")
library(lmerTest)
library(ggeffects)
library(naniar) # missing data pattern viz
source("http://janhove.github.io/RCode/CommonLanguageEffectSizes.R")
```

Load Data

```
analyzeMe <- read_csv("data.csv")
```

```
## Rows: 75 Columns: 1449
## -- Column specification --------------------------------------------------------
## Delimiter: ","
## chr    (74): ID, Sex, Race, Ethnicity, Marital_Status, employment, service_b...
## dbl  (1367): treatment, tx_grp_mod, completed, cardiac_data, number_sessions...
## lgl     (6): pre_SDANNms, pre_SDNNIms, post_SDANNms, post_SDNNIms, followup_...
## time    (2): walk_time.rb, walk_time.wk10
##
## i Use `spec()` to retrieve the full column specification for this data.
## i Specify the column types or set `show_col_types = FALSE` to quiet this message.
```

```
options(max.print = 1000)
```

Select BPI Variables and Structure Data

```
bpiPainSeverityDat <- analyzeMe %>%
        select(ID, treatment, 16:51) %>%
        rowid_to_column(., "id") %>% select(-ID) %>%
        pivot_longer(3:38, names_to = "bpiVars", values_to = "scores") %>%
        mutate(week = case_when(
                str_ends(bpiVars, "rb") ~ 0,
                str_ends(bpiVars, "w2") ~ 2,
```

```
                str_ends(bpiVars, "w4") ~ 4,
                str_ends(bpiVars, "w6") ~ 6,
                str_ends(bpiVars, "w8") ~ 8,
                str_ends(bpiVars, "w10") ~ 10,
                str_ends(bpiVars, "w18") ~ 18,
                str_ends(bpiVars, "w26") ~ 26,
                str_ends(bpiVars, "w34") ~ 34
        )) %>%
        drop_na(week) %>%
        select(-bpiVars) %>%
        group_by(id, week) %>%
        summarise(painSeverityMean = mean(scores, na.rm = T)) %>%
        ungroup() %>%
        mutate(painSeverityMean = ifelse(is.nan(painSeverityMean), NA, painSeverityMean))
## `summarise()` has grouped output by 'id'. You can override using the `.groups`
## argument.


bpiPainInterferenceDat <- analyzeMe %>%
        select(ID, treatment, baseHamd = hamdtotrb, starts_with("BPI9m")) %>%
        rowid_to_column(., "id") %>% select(-ID) %>%
        pivot_longer(4:12, names_to = "week", values_to = "painInterferenceMean") %>%
        mutate(week = case_when(
                week == "BPI9mrb" ~ 0,
                week == "BPI9mw2" ~ 2,
                week == "BPI9mw4" ~ 4,
                week == "BPI9mw6" ~ 6,
                week == "BPI9mw8" ~ 8,
                week == "BPI9mw10" ~ 10,
                week == "BPI9mw18" ~ 18,
                week == "BPI9mw26" ~ 26,
                week == "BPI9mw34" ~ 34
        ))

bpiTotalDat <- analyzeMe %>%
        select(ID, treatment, starts_with("BPIm")) %>%
        select(1:11) %>%
        rowid_to_column(., "id") %>% select(-ID) %>%
        pivot_longer(3:11, names_to = "week", values_to = "bpiTotal") %>%
        mutate(week = case_when(
                week == "BPImrb" ~ 0,
                week == "BPImw2" ~ 2,
                week == "BPImw4" ~ 4,
                week == "BPImw6" ~ 6,
                week == "BPImw8" ~ 8,
                week == "BPImw10" ~ 10,
                week == "BPImw18" ~ 18,
                week == "BPImw26" ~ 26,
                week == "BPImw34" ~ 34,

        ))

bpiDat <- left_join(bpiPainInterferenceDat, bpiPainSeverityDat, c("id", "week")) %>%
```

```
        left_join(., bpiTotalDat, by = c("id", "treatment", "week"))

bpiDat %>%
        group_by(week, treatment) %>%
        mutate(treatment = factor(treatment, levels = c(0, 1), labels = c("yoga", "cbt"))) %>%
        summarise(numNa = sum(is.na(painSeverityMean))) %>%
        ggplot(aes(week, numNa, color = treatment)) +
        geom_point() +
        geom_line() +
        scale_x_continuous(breaks = c(0, 2, 4, 6, 8, 10, 18, 26, 34)) +
        ylab("Number of Participants Missing Interference Scores")
## `summarise()` has grouped output by 'week'. You can override using the
## `.groups` argument.
```

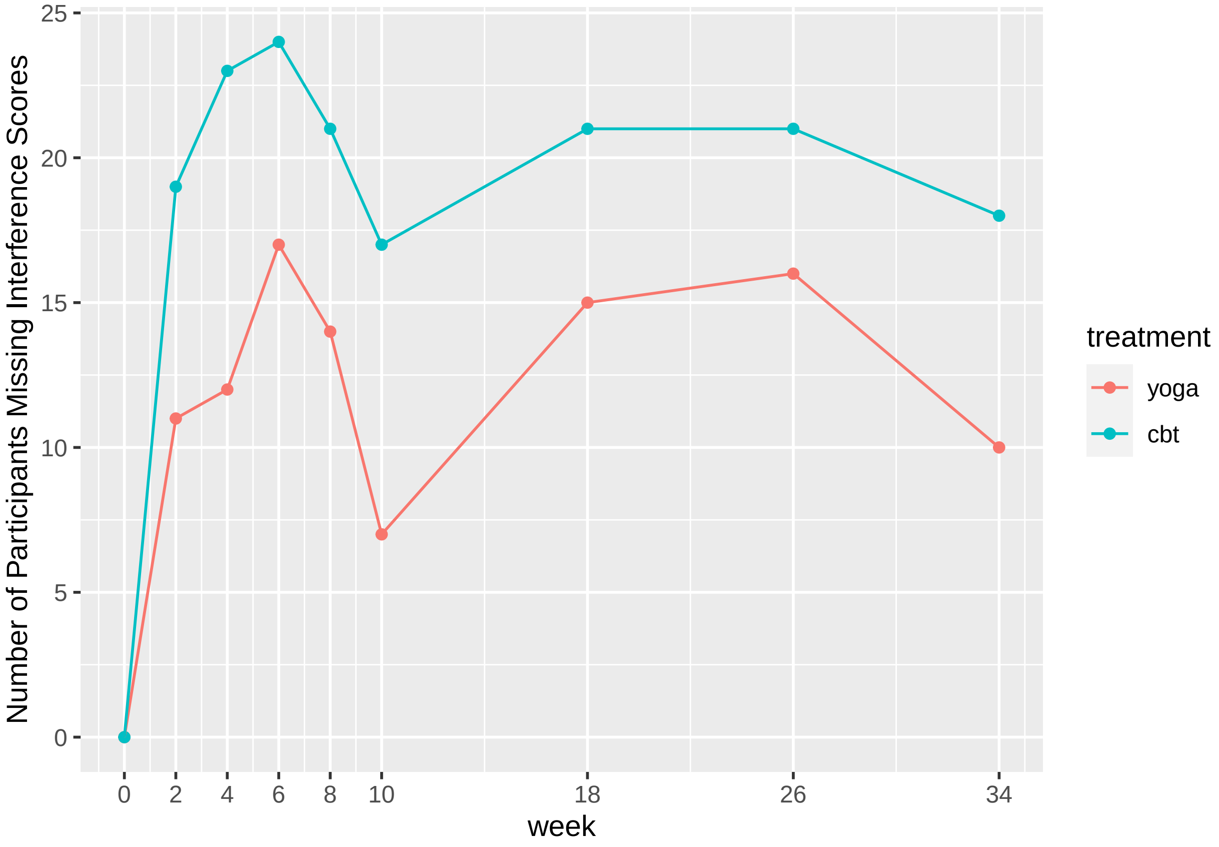

Viz BPI

```
bpiDat %>%
        group_by(treatment, week) %>%
        summarise(painIntMean = mean(painInterferenceMean, na.rm = T)) %>%
        mutate(treatment = factor(treatment, levels = c(0, 1), labels = c("yoga", "cbt"))) %>%
        ggplot(., aes(week, painIntMean, color = treatment)) +
        geom_point() +
        geom_line() +
```

```
        ylim(0, 6) +
        scale_x_continuous(breaks = c(0, 2, 4, 6, 8, 10, 18, 26, 34)) +
        ylab("Pain Interference")
## `summarise()` has grouped output by 'treatment'. You can override using the
## `.groups` argument.
```

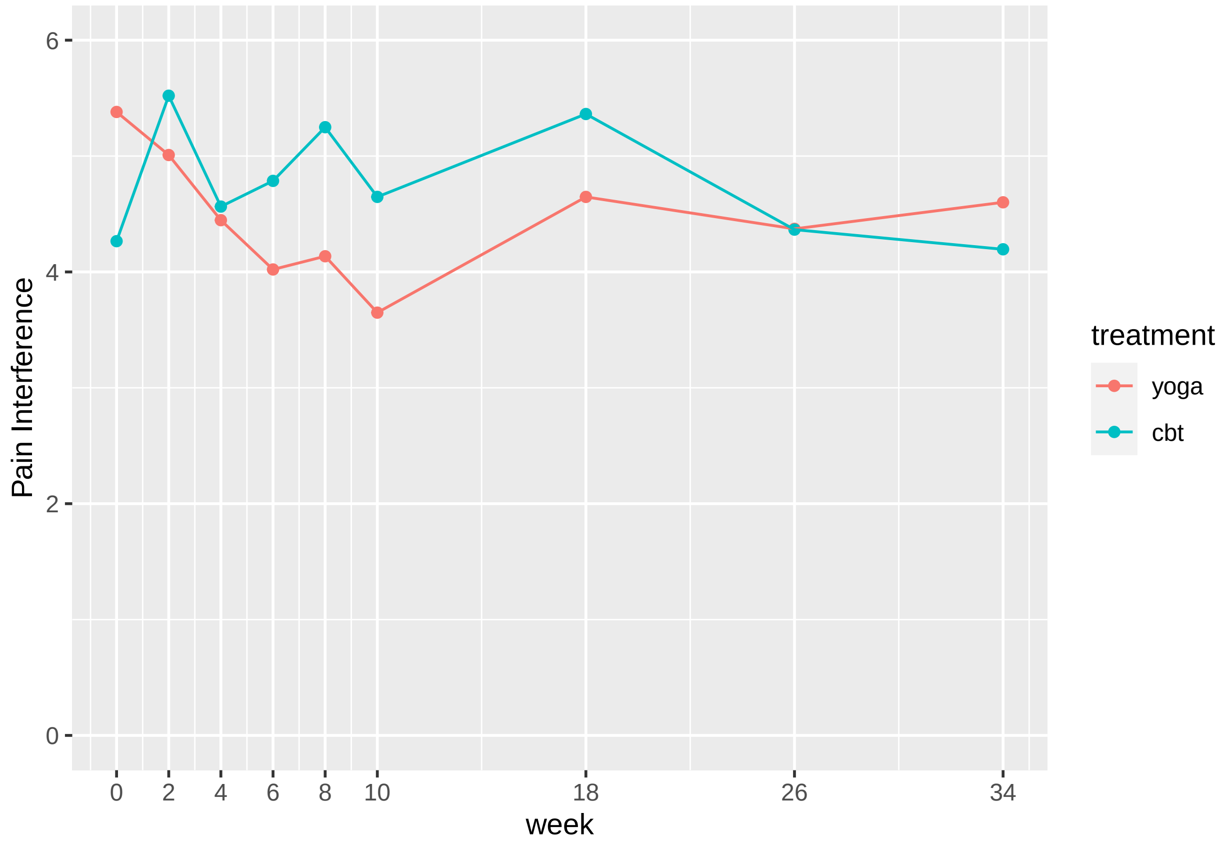


```
bpiDat %>%
        group_by(treatment, week) %>%
        mutate(treatment = factor(treatment, levels = c(0, 1), labels = c("yoga", "cbt"))) %>%
        summarise(painSevMean = mean(painSeverityMean, na.rm = T)) %>%
        ggplot(., aes(week, painSevMean, color = treatment)) +
        geom_point() +
        geom_line() +
        scale_x_continuous(breaks = c(0, 2, 4, 6, 8, 10, 18, 26, 34)) +
        ylim(0, 6) +
        ylab("Pain Severity")
## `summarise()` has grouped output by 'treatment'. You can override using the
## `.groups` argument.
```

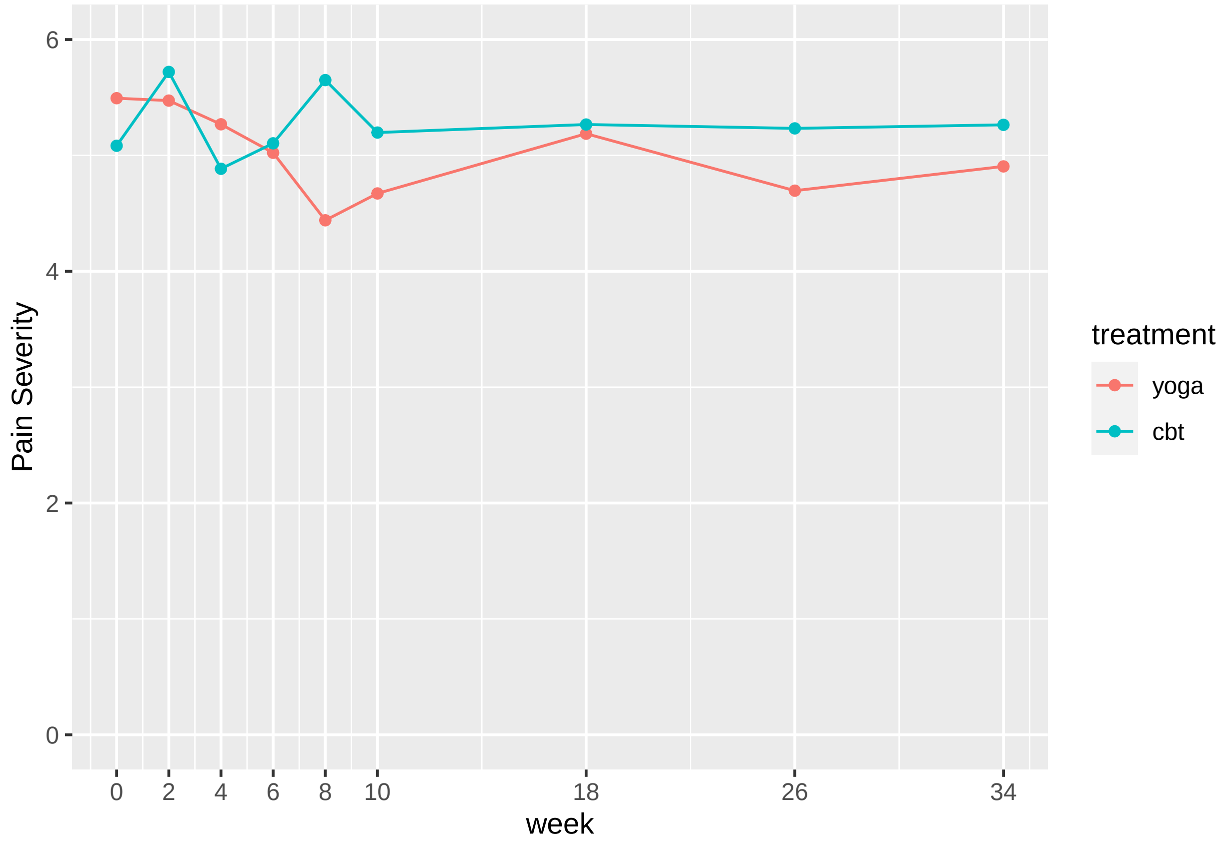

Structure Data for Piecewise Growth Models

```
bpiDat <- bpiDat %>%
        mutate(baseToEndTx = ifelse(week <= 10, week, 1)) %>%
        mutate(endOfTxToFu = case_when(
                week <= 10 ~ 0,
                week == 18 ~ 8,
                week == 26 ~ 16,
                week == 34 ~ 24
        )) %>%
        select(id, treatment, baseHamd, week, baseToEndTx, endOfTxToFu,
               painInterferenceMean, painSeverityMean, bpiTotal) %>%
        mutate(baseHamd = scale(baseHamd, scale=F))
```

Pain Severity Modeling Procedure

```
###build models: step-up procedure
#empty intercept-only model
model1 <- gls(painSeverityMean ~ 1,
                  data = bpiDat,
                  method = "REML",
```

```
                  na.action = "na.exclude")
summary(model1)
## Generalized least squares fit by REML
##   Model: painSeverityMean ~ 1
##   Data: bpiDat
##        AIC      BIC    logLik
##   1788.639 1796.662 -892.3195
##
## Coefficients:
##                Value Std.Error  t-value p-value
## (Intercept) 5.125306 0.1058102 48.43867       0
##
## Standardized residuals:
##         Min          Q1         Med          Q3         Max
## -2.39513801 -0.75953154  0.05827169  0.87607493  2.27802333
##
## Residual standard error: 2.139879
## Degrees of freedom: 409 total; 408 residual

#random intercept model
model2 <- lme(painSeverityMean ~ 1,
                 data = bpiDat,
                 method = "REML",
                 random = ~1|id,
                 na.action = "na.exclude")
summary(model2)
## Linear mixed-effects model fit by REML
##   Data: bpiDat
##        AIC      BIC    logLik
##   1408.125 1420.158 -701.0623
##
## Random effects:
##  Formula: ~1 | id
##         (Intercept) Residual
## StdDev:    1.800488 1.062031
##
## Fixed effects:  painSeverityMean ~ 1
##                Value Std.Error  DF  t-value p-value
## (Intercept) 5.119371 0.2203059 334 23.23756       0
##
## Standardized Within-Group Residuals:
##         Min          Q1         Med          Q3         Max
## -3.68372136 -0.49263323  0.01554522  0.49510300  4.33422886
##
## Number of Observations: 409
## Number of Groups: 75
anova(model1, model2)
##        Model df      AIC      BIC    logLik   Test  L.Ratio p-value
## model1     1  2 1788.639 1796.661 -892.3195
## model2     2  3 1408.124 1420.158 -701.0623 1 vs 2 382.5145  <.0001
icc(model2)
## [1] 0.74
```

```r
#add fixed effects for time, fixed linear time model
model3 <- lme(painSeverityMean ~ baseToEndTx + endOfTxToFu,
                 data = bpiDat,
                 method = "REML",
                 random = ~1|id,
                 na.action = "na.exclude")
summary(model3)
## Linear mixed-effects model fit by REML
##   Data: bpiDat
##        AIC      BIC    logLik
##   1411.11 1431.141 -700.5548
##
## Random effects:
##  Formula: ~1 | id
##         (Intercept) Residual
## StdDev:    1.809223 1.040112
##
## Fixed effects:  painSeverityMean ~ baseToEndTx + endOfTxToFu
##                  Value  Std.Error  DF   t-value p-value
## (Intercept)  5.370640 0.23009595 332 23.340870  0.0000
## baseToEndTx -0.067161 0.01705993 332 -3.936784  0.0001
## endOfTxToFu -0.016544 0.00703114 332 -2.352997  0.0192
##  Correlation:
##             (Intr) bsTEnT
## baseToEndTx -0.259
## endOfTxToFu -0.213  0.450
##
## Standardized Within-Group Residuals:
##         Min          Q1         Med          Q3         Max
## -3.39881874 -0.48793167 -0.04395554  0.52739319  4.22316722
##
## Number of Observations: 409
## Number of Groups: 75


#add random effect for endOfTxToFu, random linear time model
model4 <- lme(painSeverityMean ~ baseToEndTx + endOfTxToFu,
                 data = bpiDat,
                 method = "REML",
                 random = ~endOfTxToFu|id,
                 na.action = "na.exclude")
summary(model4)
## Linear mixed-effects model fit by REML
##   Data: bpiDat
##         AIC      BIC    logLik
##   1414.017 1442.062 -700.0086
##
## Random effects:
##  Formula: ~endOfTxToFu | id
##  Structure: General positive-definite, Log-Cholesky parametrization
##             StdDev     Corr
## (Intercept) 1.78049389 (Intr)
## endOfTxToFu 0.01321448 0.432
```

```
## Residual    1.03397004
##
## Fixed effects:  painSeverityMean ~ baseToEndTx + endOfTxToFu
##                  Value  Std.Error  DF   t-value p-value
## (Intercept)  5.372086 0.22683727 332 23.682553  0.0000
## baseToEndTx -0.066921 0.01695655 332 -3.946594  0.0001
## endOfTxToFu -0.016899 0.00729190 332 -2.317521  0.0211
##  Correlation:
##             (Intr) bsTEnT
## baseToEndTx -0.262
## endOfTxToFu -0.122  0.439
##
## Standardized Within-Group Residuals:
##         Min          Q1         Med          Q3         Max
## -3.41975097 -0.49481810 -0.02791689  0.50671018  4.22195542
##
## Number of Observations: 409
## Number of Groups: 75
anova(model3, model4) # not a better fit
##        Model df      AIC      BIC    logLik   Test  L.Ratio p-value
## model3     1  5 1411.110 1431.141 -700.5548
## model4     2  7 1414.017 1442.062 -700.0086 1 vs 2 1.092551  0.5791

#add random effect for baseToEndTx, random linear time model --best fitting base model--
model5 <- lme(painSeverityMean ~ baseToEndTx + endOfTxToFu,
                 data = bpiDat,
                 method = "REML",
                 random = ~baseToEndTx|id,
                 na.action = "na.exclude")
summary(model5)
## Linear mixed-effects model fit by REML
##   Data: bpiDat
##        AIC      BIC    logLik
##   1401.848 1429.892 -693.9239
##
## Random effects:
##  Formula: ~baseToEndTx | id
##  Structure: General positive-definite, Log-Cholesky parametrization
##             StdDev     Corr
## (Intercept) 1.70653110 (Intr)
## baseToEndTx 0.09690324 0.289
## Residual    0.98127639
##
## Fixed effects:  painSeverityMean ~ baseToEndTx + endOfTxToFu
##                  Value  Std.Error  DF   t-value p-value
## (Intercept)  5.376192 0.21707590 332 24.766416  0.0000
## baseToEndTx -0.070290 0.02115378 332 -3.322833  0.0010
## endOfTxToFu -0.015858 0.00665317 332 -2.383502  0.0177
##  Correlation:
##             (Intr) bsTEnT
## baseToEndTx -0.056
## endOfTxToFu -0.213  0.352
##
```

```
## Standardized Within-Group Residuals:
##         Min          Q1         Med          Q3         Max
## -3.08881581 -0.46715064 -0.04273285  0.51852841  4.24944053
##
## Number of Observations: 409
## Number of Groups: 75
anova(model3, model5) # better fit
##        Model df      AIC      BIC    logLik   Test  L.Ratio p-value
## model3     1  5 1411.110 1431.141 -700.5548
## model5     2  7 1401.848 1429.892 -693.9239 1 vs 2 13.26194  0.0013

#add random effect for both endOfTxToFu and baseToEndTx, random linear time model
model6 <- lme(painSeverityMean ~ baseToEndTx + endOfTxToFu,
                 data = bpiDat,
                 method = "REML",
                 random = ~baseToEndTx + endOfTxToFu | id,
                 na.action = "na.exclude")
summary(model6)
## Linear mixed-effects model fit by REML
##   Data: bpiDat
##        AIC      BIC    logLik
##   1389.201 1429.264 -684.6005
##
## Random effects:
##  Formula: ~baseToEndTx + endOfTxToFu | id
##  Structure: General positive-definite, Log-Cholesky parametrization
##             StdDev     Corr
## (Intercept) 1.65398175 (Intr) bsTEnT
## baseToEndTx 0.13900222 0.099
## endOfTxToFu 0.04638885 0.055  0.928
## Residual    0.91817623
##
## Fixed effects:  painSeverityMean ~ baseToEndTx + endOfTxToFu
##                  Value  Std.Error  DF   t-value p-value
## (Intercept)   5.378940 0.20921324 332 25.710323  0.0000
## baseToEndTx -0.071376 0.02446742 332 -2.917186  0.0038
## endOfTxToFu -0.018227 0.00897649 332 -2.030509  0.0431
##  Correlation:
##             (Intr) bsTEnT
## baseToEndTx -0.096
## endOfTxToFu -0.114  0.711
##
## Standardized Within-Group Residuals:
##         Min          Q1         Med          Q3         Max
## -3.14123410 -0.48452307 -0.03820371  0.46355048  3.85155850
##
## Number of Observations: 409
## Number of Groups: 75
anova(model4, model6) # better fit
##        Model df      AIC      BIC    logLik   Test  L.Ratio p-value
## model4     1  7 1414.017 1442.062 -700.0086
## model6     2 10 1389.201 1429.264 -684.6005 1 vs 2 30.81621  <.0001
```

```
#test model6 with lmer package for convergence
mod6.5 <- lme4::lmer(painSeverityMean ~ baseToEndTx + endOfTxToFu +
                             (baseToEndTx + endOfTxToFu | id),
               data = bpiDat)

# best fitting model - per random effects plot below
model7 <- lme(painSeverityMean ~ baseHamd + baseToEndTx*treatment + endOfTxToFu*treatment,
                 data = bpiDat,
                 method = "REML",
                 random = ~baseToEndTx + endOfTxToFu | id,
                 na.action = "na.exclude")
summary(model7)
## Linear mixed-effects model fit by REML
##   Data: bpiDat
##        AIC      BIC    logLik
##   1314.003 1369.018 -643.0017
##
## Random effects:
##  Formula: ~baseToEndTx + endOfTxToFu | id
##  Structure: General positive-definite, Log-Cholesky parametrization
##             StdDev     Corr
## (Intercept) 1.43071036 (Intr) bsTEnT
## baseToEndTx 0.14248689 0.178
## endOfTxToFu 0.04360271 0.172  0.914
## Residual    0.92585972
##
## Fixed effects:  painSeverityMean ~ baseHamd + baseToEndTx * treatment + endOfTxToFu *      treatment
##                           Value Std.Error  DF   t-value p-value
## (Intercept)            5.438444 0.2693050 308 20.194370  0.0000
## baseHamd               0.117399 0.0264838  68  4.432861  0.0000
## baseToEndTx           -0.089272 0.0334732 308 -2.666979  0.0081
## treatment             -0.064684 0.3924588  68 -0.164817  0.8696
## endOfTxToFu           -0.032581 0.0117849 308 -2.764661  0.0060
## baseToEndTx:treatment  0.052639 0.0523266 308  1.005968  0.3152
## treatment:endOfTxToFu  0.036594 0.0183558 308  1.993578  0.0471
##  Correlation:
##                       (Intr) basHmd bsTEnT trtmnt enOTTF bsTET:
## baseHamd              -0.152
## baseToEndTx           -0.072  0.007
## treatment             -0.703  0.217  0.050
## endOfTxToFu           -0.082  0.011  0.700  0.058
## baseToEndTx:treatment  0.045  0.000 -0.640 -0.068 -0.448
## treatment:endOfTxToFu  0.052 -0.001 -0.449 -0.077 -0.642  0.687
##
## Standardized Within-Group Residuals:
##         Min          Q1         Med          Q3         Max
## -3.03615302 -0.47858068 -0.04866581  0.50308878  3.84127173
##
## Number of Observations: 383
## Number of Groups: 71
intervals(model7)
## Approximate 95% confidence intervals
##
```

```
##  Fixed effects:
##                               lower        est.        upper
## (Intercept)            4.9085338980  5.43844421  5.968354524
## baseHamd               0.0645513504  0.11739892  0.170246480
## baseToEndTx           -0.1551373409 -0.08927228 -0.023407227
## treatment             -0.8478234543 -0.06468403  0.718455387
## endOfTxToFu           -0.0557704766 -0.03258132 -0.009392173
## baseToEndTx:treatment -0.0503239829  0.05263892  0.155601819
## treatment:endOfTxToFu  0.0004750809  0.03659374  0.072712404
##
##  Random Effects:
##   Level: id
##                                    lower       est.     upper
## sd((Intercept))               1.15598555 1.43071036 1.7707247
## sd(baseToEndTx)               0.10068726 0.14248689 0.2016394
## sd(endOfTxToFu)               0.02690775 0.04360271 0.0706561
## cor((Intercept),baseToEndTx) -0.24969699 0.17832251 0.5480492
## cor((Intercept),endOfTxToFu) -0.30233771 0.17169947 0.5776475
## cor(baseToEndTx,endOfTxToFu)  0.02653243 0.91440758 0.9957930
##
##  Within-group standard error:
##     lower      est.     upper
## 0.8427645 0.9258597 1.0171480

#cohen's d
#https://stats.stackexchange.com/questions/257985/how-can-i-derive-effect-sizes-in-lme4-and-describe-th
nlmeCohenD <- function(meanDif, model) {
        cohenD <- meanDif / (sqrt(as.numeric(VarCorr(model)[1,1]) +
                                as.numeric(VarCorr(model)[2,1]) +
                                as.numeric(VarCorr(model)[3,1])))
        cohenD <- as.numeric(cohenD)
        return(cohenD)
}

painSevCohenDat <- bpiDat %>%
        group_by(treatment, week) %>%
        summarise(meanPainSev = mean(painSeverityMean, na.rm = T)) %>%
        ungroup() %>%
        group_by(week) %>%
        mutate(meanDif = last(meanPainSev) - first(meanPainSev)) %>%
        ungroup() %>%
        mutate(painSevCohenD = purrr::map_dbl(meanDif, nlmeCohenD, model7)) %>%
        filter(week >= 10) %>%
        select(treatment, week, meanPainSev, painSevCohenD)
## `summarise()` has grouped output by 'treatment'. You can override using the
## `.groups` argument.

#plot model8
#https://stats.stackexchange.com/questions/184525/how-to-determine-whether-or-not-the-y-axis-of-a-graph
painSevFig <- plot(ggpredict(model7, c("endOfTxToFu", "treatment"), ci.lvl = NA), use.theme = F,
     connect.lines = TRUE, colors = "bw") +
        ylim(3,6) +
        geom_point() +
```

```
        theme(axis.text=element_text(size = 16),
                                            axis.title=element_text(size = 18, face="bold"),
                                            strip.text.x = element_text(size = 18)) +
        scale_color_manual(name = "",
                           labels = c("Yoga", "CBT"),
                           values = c("#000000", "#000000"),
                           guide = guide_legend(reverse = F)) +
        scale_linetype_manual(name = "",
                           labels = c("Yoga", "CBT"),
                           values = c("dashed", "solid"),
                           guide = guide_legend(reverse = F)) +
        jtools::theme_apa(legend.pos = "top", x.font.size = 18, y.font.size = 18,
                          facet.title.size = 18, legend.use.title = F) +
        scale_x_continuous(breaks=c(0, 8, 16, 24),
                       labels=c("EOT", "2-mos", "4-mos", "6-mos")) +
        theme(axis.text.x = element_text(size = 18),
              axis.text.y = element_text(size = 18),
              panel.border = element_blank()) +
        labs(tag = "A", title = "", size = 18) +
        xlab("") + ylab("BPI-SF Pain Severity") +
        theme(axis.line = element_line(color = "black")) +
        theme(legend.text=element_text(size=18))
## Scale for y is already present.
## Adding another scale for y, which will replace the existing scale.
## Scale for colour is already present.
## Adding another scale for colour, which will replace the existing scale.
```

Check Assumptions: Model 8

```
#check for independence and normality of within-group errors (participants)
plot(model7, resid(., type="p") ~ fitted(.), abline=0)
```

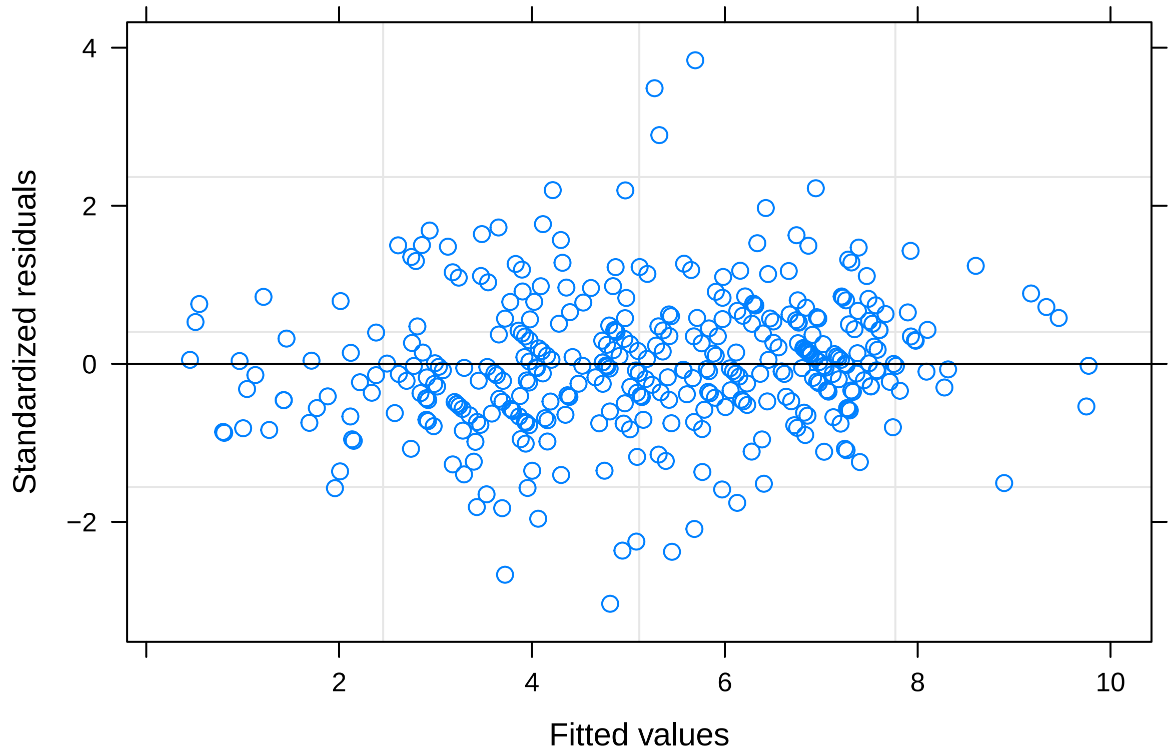


```
plot(model7, id~resid(.), abline = 0 )
```

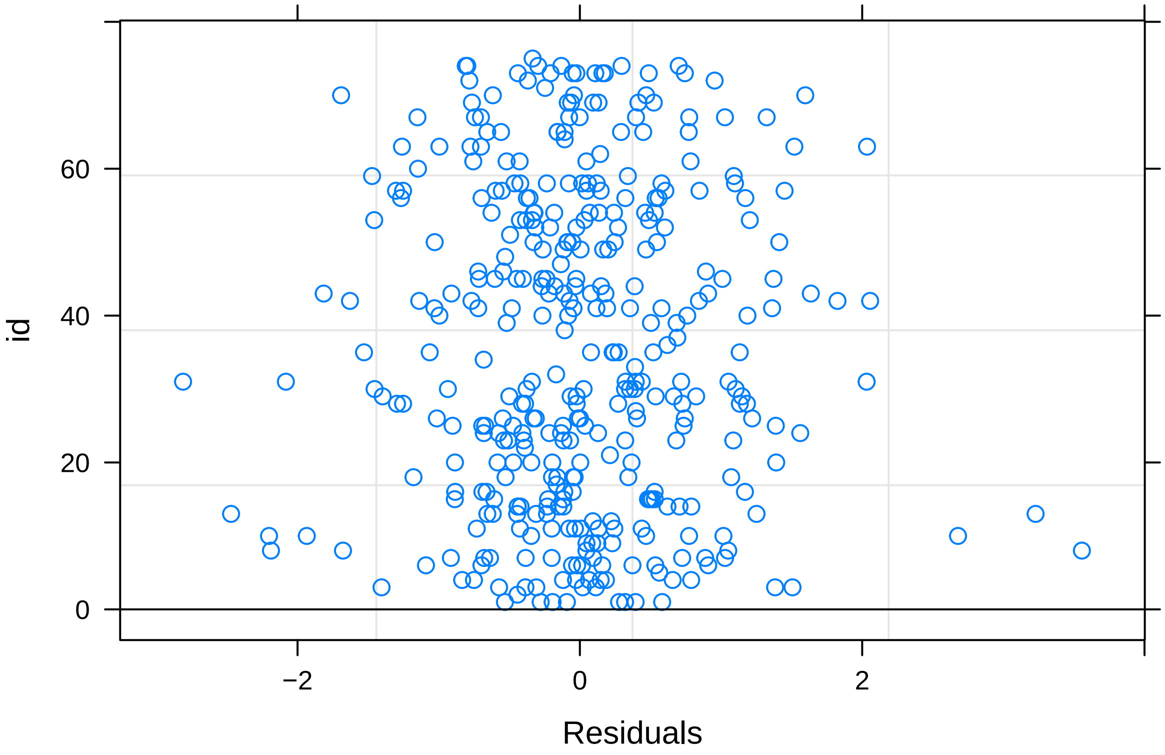


```
plot(model7, resid(., type = "p") ~ fitted(.) | week, id = 0.05, adj = -0.3 )
```

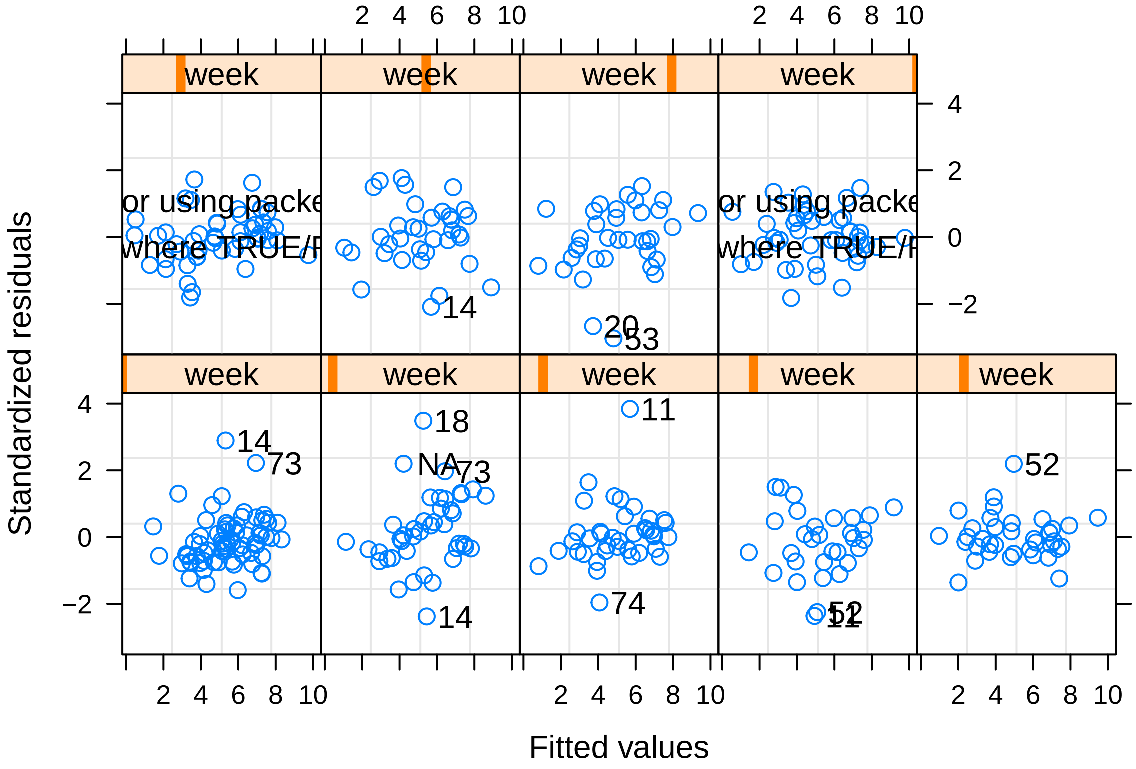


```
plot(model7, painSeverityMean ~ fitted(.) | week, id = 0.05, adj = -0.3 )
```

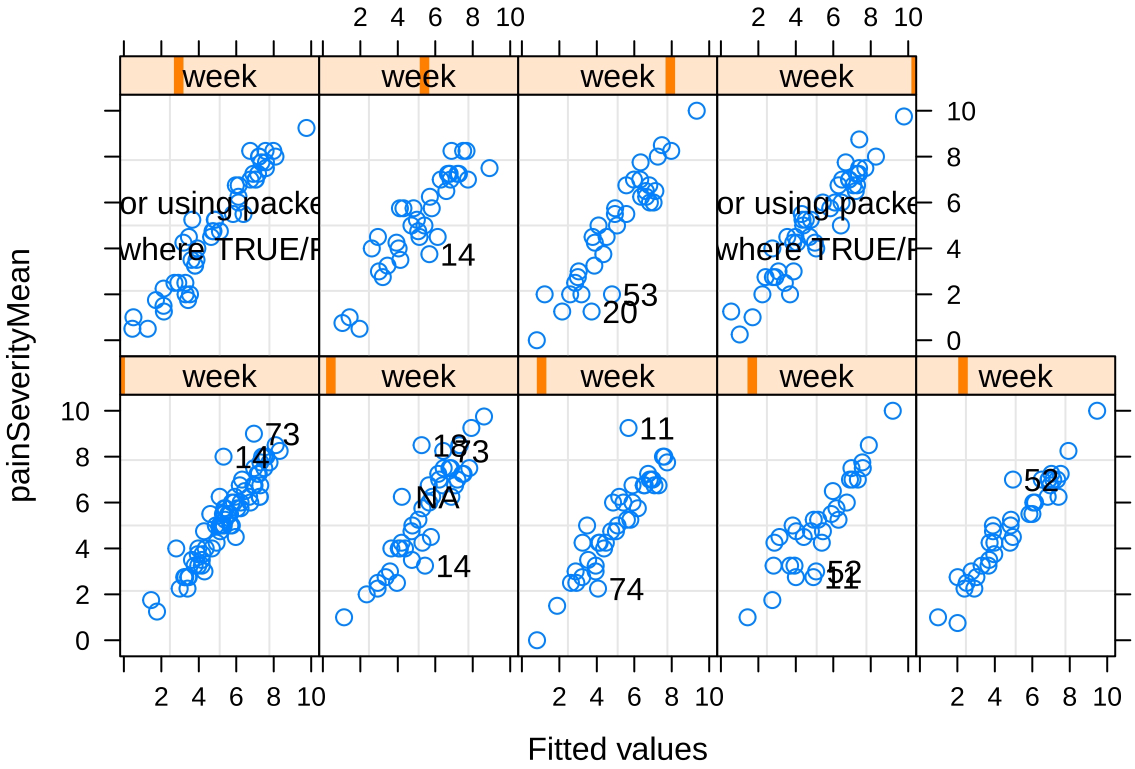

```
plot(model7, painSeverityMean ~ fitted(.), id=.05, adj=-0.3)
```

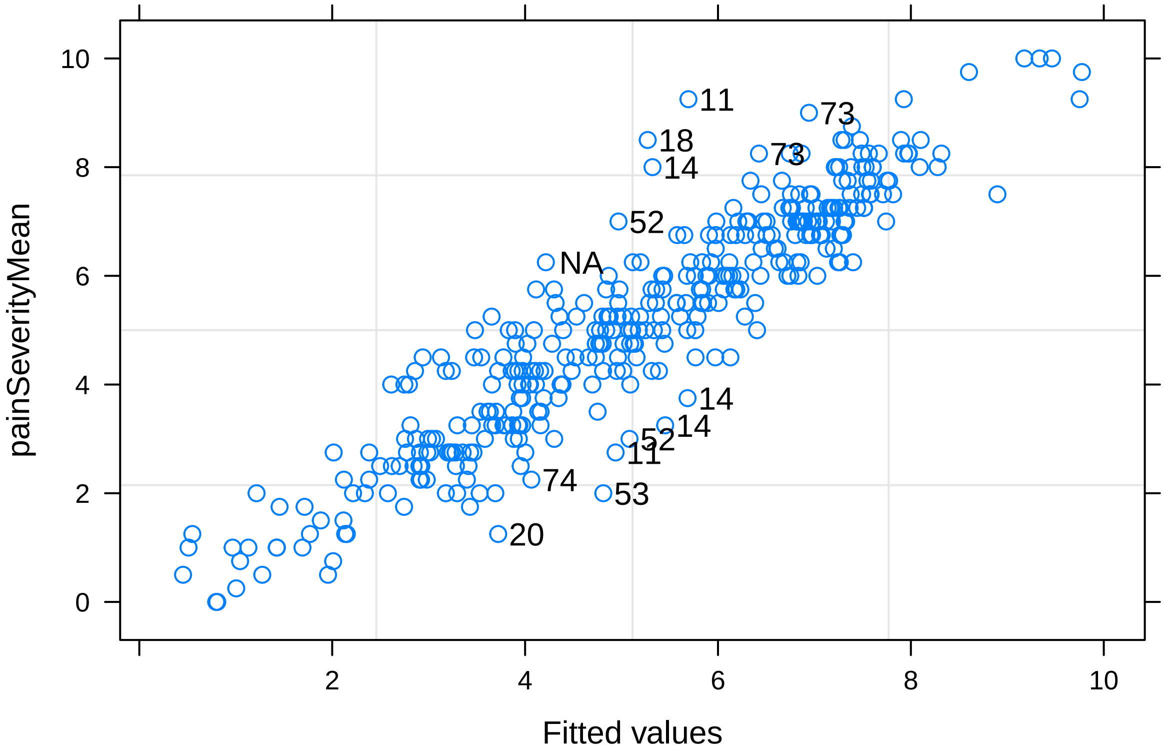


```
hist(residuals(model7), breaks=20, id=.05)
## Warning in plot.window(xlim, ylim, "", ...): "id" is not a graphical parameter
## Warning in title(main = main, sub = sub, xlab = xlab, ylab = ylab, ...): "id"
## is not a graphical parameter
## Warning in axis(1, ...): "id" is not a graphical parameter
## Warning in axis(2, at = yt, ...): "id" is not a graphical parameter
```

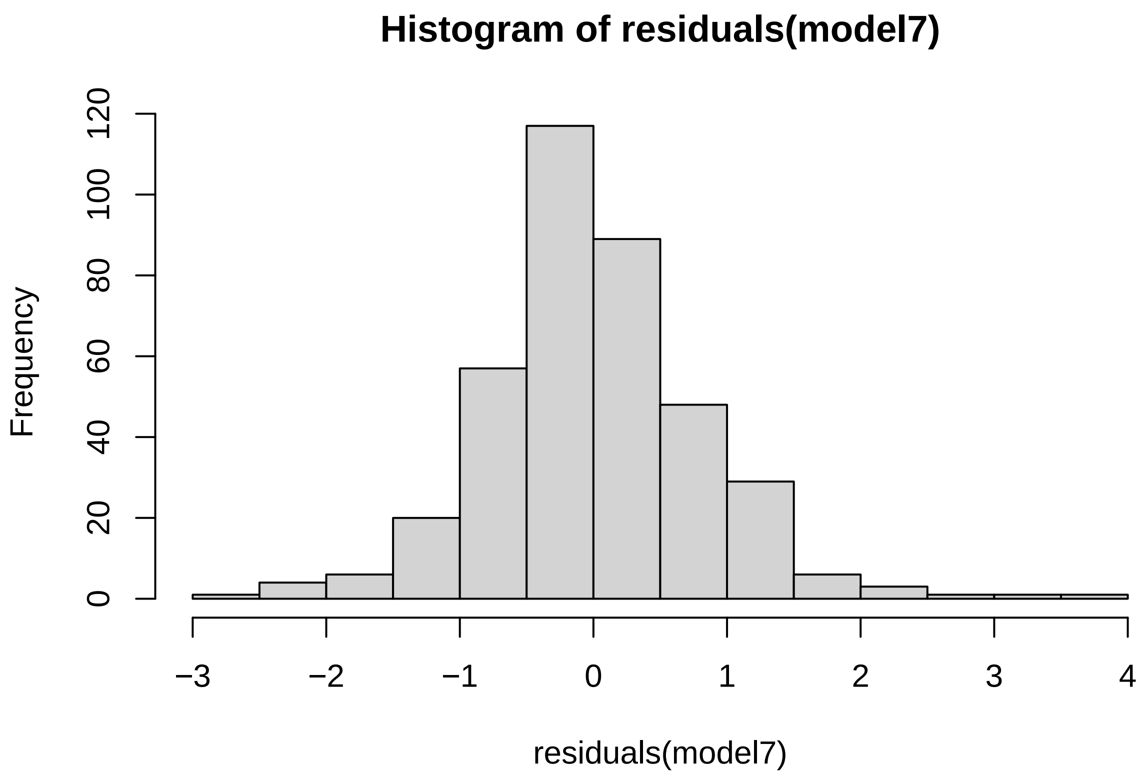


```
qqnorm(model7)
```

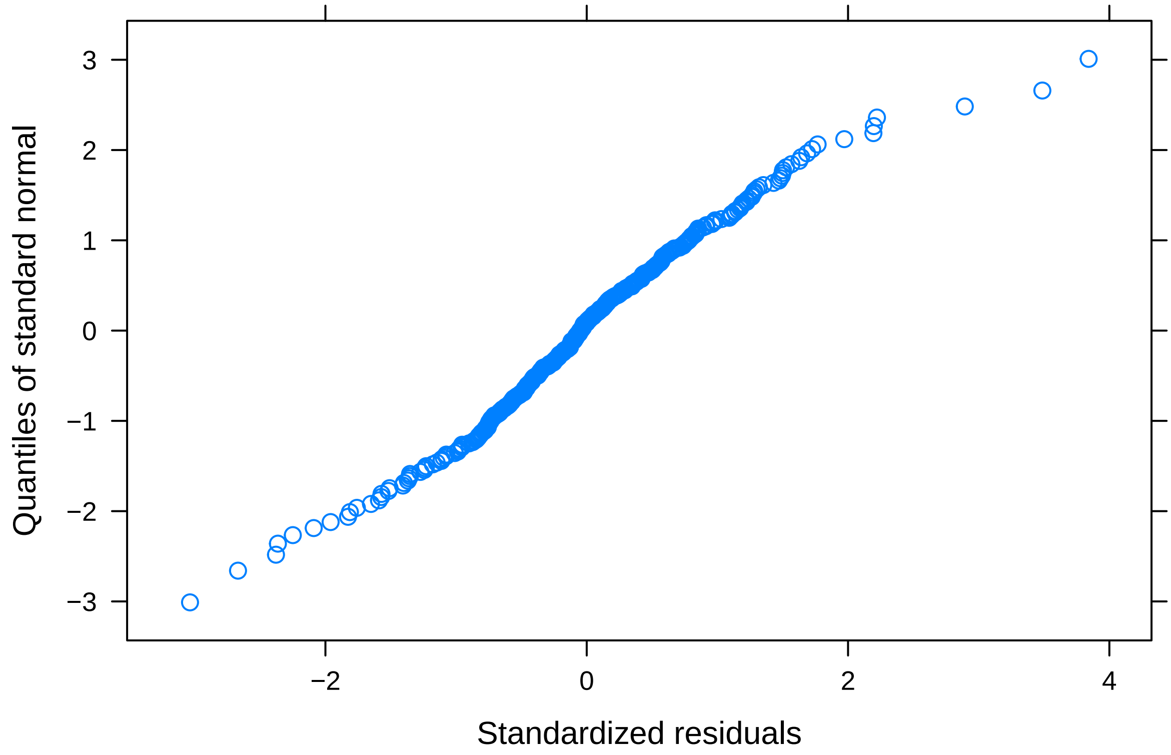

```r
#check for normality and independence of random effects
qqnorm(model7, ~ranef(.), id=0.10, cex=0.7)
```

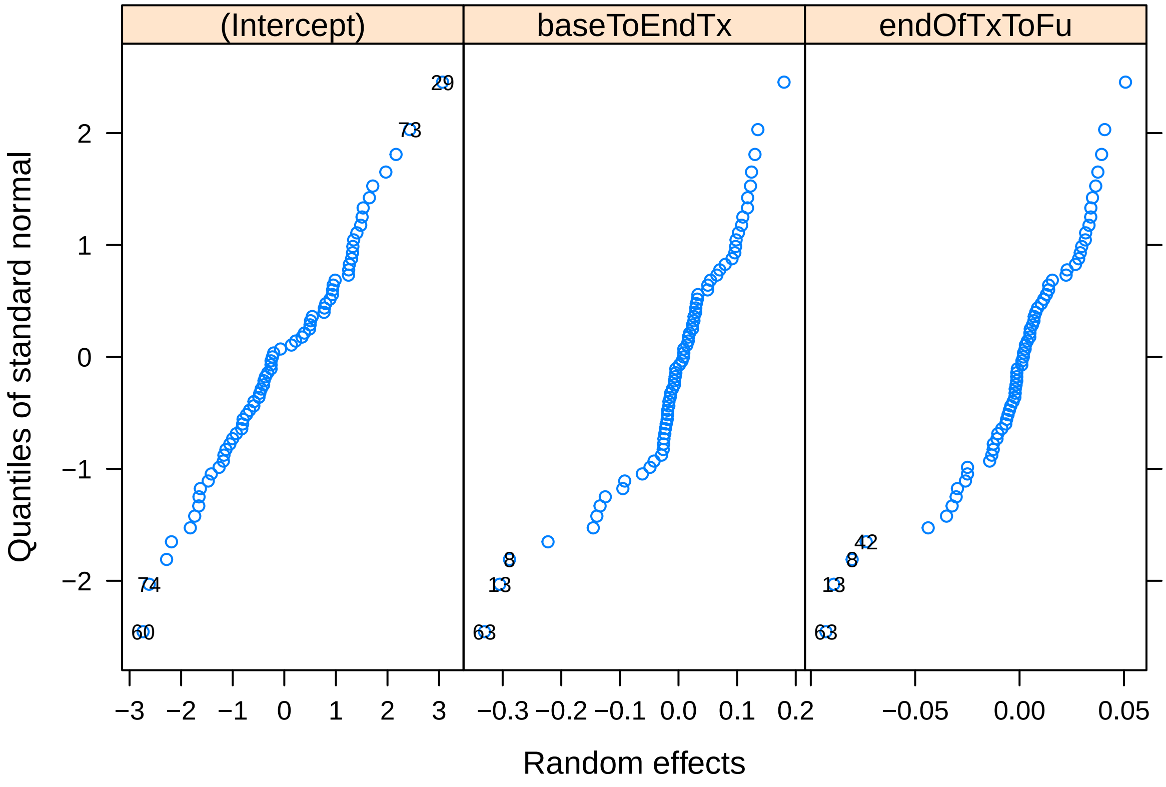


```r
pairs(model7, ~ranef(., level=1), adj=-0.3)
```

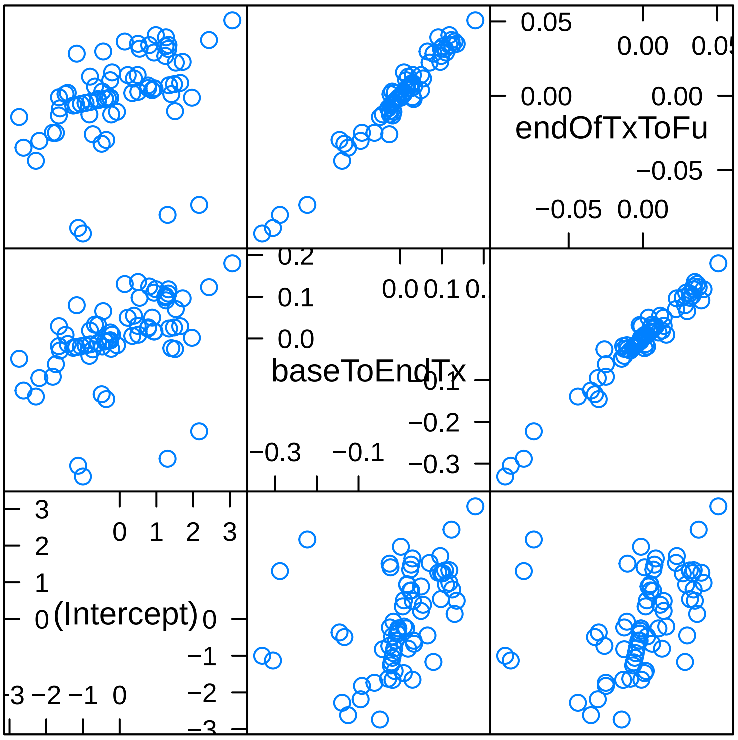

Scatter Plot Matrix

Pain Interference Modeling Procedure

```
###build models: step-up procedure
#empty intercept-only model
model1 <- gls(painInterferenceMean ~ 1,
                  data = bpiDat,
                  method = "REML",
                  na.action = "na.exclude")
summary(model1)
## Generalized least squares fit by REML
##   Model: painInterferenceMean ~ 1
##   Data: bpiDat
##        AIC      BIC    logLik
##   1825.373 1833.326 -910.6866
##
## Coefficients:
##                Value Std.Error  t-value p-value
## (Intercept) 4.59455 0.1218967 37.69217       0
##
## Standardized residuals:
##         Min          Q1         Med          Q3         Max
## -1.89649900 -0.79577515  0.02976773  0.76358363  2.23121542
##
## Residual standard error: 2.422648
## Degrees of freedom: 395 total; 394 residual
```

```r
#random intercept model
model2 <- lme(painInterferenceMean ~ 1,
                 data = bpiDat,
                 method = "REML",
                 random = ~1|id,
                 na.action = "na.exclude")
summary(model2)
## Linear mixed-effects model fit by REML
##   Data: bpiDat
##        AIC      BIC    logLik
##   1526.439 1538.368 -760.2197
##
## Random effects:
##  Formula: ~1 | id
##         (Intercept) Residual
## StdDev:     2.04679 1.327045
##
## Fixed effects:  painInterferenceMean ~ 1
##                Value Std.Error  DF  t-value p-value
## (Intercept) 4.581061 0.2547373 321 17.98347       0
##
## Standardized Within-Group Residuals:
##         Min          Q1         Med          Q3         Max
## -3.09451504 -0.47518712 -0.03046783  0.57731526  2.80984005
##
## Number of Observations: 395
## Number of Groups: 74
anova(model1, model2)
##        Model df      AIC      BIC    logLik   Test  L.Ratio p-value
## model1     1  2 1825.373 1833.326 -910.6866
## model2     2  3 1526.439 1538.368 -760.2197 1 vs 2 300.9338  <.0001
icc(model2)
## [1] 0.7

#add fixed effects for time, fixed linear time model
model3 <- lme(painInterferenceMean ~ baseToEndTx + endOfTxToFu,
                 data = bpiDat,
                 method = "REML",
                 random = ~1|id,
                 na.action = "na.exclude")
summary(model3)
## Linear mixed-effects model fit by REML
##   Data: bpiDat
##        AIC      BIC    logLik
##   1517.412 1537.268 -753.7061
##
## Random effects:
##  Formula: ~1 | id
##         (Intercept) Residual
## StdDev:    2.078954 1.274387
##
## Fixed effects:  painInterferenceMean ~ baseToEndTx + endOfTxToFu
##                 Value  Std.Error  DF   t-value p-value
```

```
## (Intercept)  4.966427 0.26946911 319 18.430413  0.0000
## baseToEndTx -0.112863 0.02141395 319 -5.270552  0.0000
## endOfTxToFu -0.018740 0.00879569 319 -2.130582  0.0339
##  Correlation:
##             (Intr) bsTEnT
## baseToEndTx -0.276
## endOfTxToFu -0.224  0.437
##
## Standardized Within-Group Residuals:
##          Min           Q1          Med           Q3          Max
## -2.861413708 -0.528226440 -0.004418457  0.529530229  3.125958927
##
## Number of Observations: 395
## Number of Groups: 74


#add random effect for endOfTxToFu, random linear time model
model4 <- lme(painInterferenceMean ~ baseToEndTx + endOfTxToFu,
                 data = bpiDat,
                 method = "REML",
                 random = ~endOfTxToFu|id,
                 na.action = "na.exclude")
summary(model4)
## Linear mixed-effects model fit by REML
##   Data: bpiDat
##        AIC      BIC    logLik
##   1517.689 1545.488 -751.8443
##
## Random effects:
##  Formula: ~endOfTxToFu | id
##  Structure: General positive-definite, Log-Cholesky parametrization
##             StdDev     Corr
## (Intercept) 2.01223018 (Intr)
## endOfTxToFu 0.02302361 0.663
## Residual    1.25959972
##
## Fixed effects:  painInterferenceMean ~ baseToEndTx + endOfTxToFu
##                  Value  Std.Error  DF   t-value p-value
## (Intercept)  4.966789 0.26188018 319 18.965883  0.0000
## baseToEndTx -0.111410 0.02115133 319 -5.267257  0.0000
## endOfTxToFu -0.018908 0.00940710 319 -2.009929  0.0453
##  Correlation:
##             (Intr) bsTEnT
## baseToEndTx -0.281
## endOfTxToFu -0.036  0.421
##
## Standardized Within-Group Residuals:
##         Min          Q1         Med          Q3         Max
## -2.90096709 -0.50845325  0.01568676  0.52873392  3.13870886
##
## Number of Observations: 395
## Number of Groups: 74
anova(model3, model4) # not a better fit
```

```
##        Model df      AIC      BIC    logLik   Test L.Ratio p-value
## model3     1  5 1517.412 1537.268 -753.7061
## model4     2  7 1517.689 1545.487 -751.8443 1 vs 2 3.72346  0.1554

#add random effect for baseToEndTx, random linear time model --best fitting base model--
model5 <- lme(painInterferenceMean ~ baseToEndTx + endOfTxToFu,
                 data = bpiDat,
                 method = "REML",
                 random = ~baseToEndTx|id,
                 na.action = "na.exclude")
summary(model5)
## Linear mixed-effects model fit by REML
##   Data: bpiDat
##        AIC      BIC    logLik
##   1513.815 1541.614 -749.9074
##
## Random effects:
##  Formula: ~baseToEndTx | id
##  Structure: General positive-definite, Log-Cholesky parametrization
##             StdDev    Corr
## (Intercept) 2.1454945 (Intr)
## baseToEndTx 0.1190884 -0.272
## Residual    1.2023849
##
## Fixed effects:  painInterferenceMean ~ baseToEndTx + endOfTxToFu
##                 Value  Std.Error  DF   t-value p-value
## (Intercept)  4.966485 0.27373979 319 18.143087  0.0000
## baseToEndTx -0.110541 0.02598091 319 -4.254681  0.0000
## endOfTxToFu -0.018397 0.00835294 319 -2.202451  0.0283
##  Correlation:
##             (Intr) bsTEnT
## baseToEndTx -0.338
## endOfTxToFu -0.205  0.328
##
## Standardized Within-Group Residuals:
##         Min          Q1         Med          Q3         Max
## -2.66270271 -0.52718371 -0.01429268  0.52787094  3.27133401
##
## Number of Observations: 395
## Number of Groups: 74
anova(model3, model5) # better fit
##        Model df      AIC      BIC    logLik   Test  L.Ratio p-value
## model3     1  5 1517.412 1537.268 -753.7061
## model5     2  7 1513.815 1541.614 -749.9074 1 vs 2 7.597416  0.0224

#add random effect for both endOfTxToFu and baseToEndTx, random linear time model
model6 <- lme(painInterferenceMean ~ baseToEndTx + endOfTxToFu,
                 data = bpiDat,
                 method = "REML",
                 random = ~baseToEndTx + endOfTxToFu | id,
                 na.action = "na.exclude")
summary(model6)
## Linear mixed-effects model fit by REML
```

```
##   Data: bpiDat
##        AIC      BIC   logLik
##   1515.146 1554.859 -747.573
##
## Random effects:
##  Formula: ~baseToEndTx + endOfTxToFu | id
##  Structure: General positive-definite, Log-Cholesky parametrization
##             StdDev     Corr
## (Intercept) 2.05369731 (Intr) bsTEnT
## baseToEndTx 0.13236726 -0.201
## endOfTxToFu 0.03093057  0.404  0.458
## Residual    1.17614094
##
## Fixed effects:  painInterferenceMean ~ baseToEndTx + endOfTxToFu
##                  Value  Std.Error  DF   t-value p-value
## (Intercept)  4.967018 0.26301225 319 18.885119  0.0000
## baseToEndTx -0.109925 0.02690629 319 -4.085478  0.0001
## endOfTxToFu -0.018632 0.00935338 319 -1.992029  0.0472
##  Correlation:
##             (Intr) bsTEnT
## baseToEndTx -0.303
## endOfTxToFu -0.038  0.429
##
## Standardized Within-Group Residuals:
##        Min         Q1        Med         Q3        Max
## -2.5929074 -0.5234285  0.0199741  0.5124456  3.3445007
##
## Number of Observations: 395
## Number of Groups: 74
anova(model4, model6) # better fit
##        Model df      AIC      BIC    logLik   Test  L.Ratio p-value
## model4     1  7 1517.689 1545.487 -751.8443
## model6     2 10 1515.146 1554.859 -747.5730 1 vs 2 8.542613   0.036

#test model6 with lmer package for convergence --FAILS TO CONVERGE: USE model5 AS BASE MODEL--
mod6.5 <- lme4::lmer(painInterferenceMean ~ baseToEndTx + endOfTxToFu + (baseToEndTx + endOfTxToFu | id
               data = bpiDat)
## Warning in checkConv(attr(opt, "derivs"), opt$par, ctrl = control$checkConv, :
## Model failed to converge with max|grad| = 0.00926882 (tol = 0.002, component 1)

#test convergence of model5 with tx interactions
model6 <- lme(painInterferenceMean ~ baseToEndTx*treatment + endOfTxToFu*treatment,
              data = bpiDat,
              method = "REML",
              na.action = "na.exclude",
              random = ~baseToEndTx|id)

summary(model6)
## Linear mixed-effects model fit by REML
##   Data: bpiDat
##        AIC     BIC    logLik
##   1523.735 1563.37 -751.8673
##
```

```
## Random effects:
##  Formula: ~baseToEndTx | id
##  Structure: General positive-definite, Log-Cholesky parametrization
##             StdDev    Corr
## (Intercept) 2.1390970 (Intr)
## baseToEndTx 0.1142529 -0.307
## Residual    1.2024045
##
## Fixed effects:  painInterferenceMean ~ baseToEndTx * treatment + endOfTxToFu *      treatment
##                          Value Std.Error  DF   t-value p-value
## (Intercept)           5.343861 0.3727976 317 14.334484  0.0000
## baseToEndTx          -0.157337 0.0322914 317 -4.872395  0.0000
## treatment            -0.825639 0.5476051  72 -1.507728  0.1360
## endOfTxToFu          -0.026456 0.0106300 317 -2.488833  0.0133
## baseToEndTx:treatment  0.123028 0.0528138 317  2.329475  0.0205
## treatment:endOfTxToFu  0.020191 0.0171920 317  1.174465  0.2411
##  Correlation:
##                      (Intr) bsTEnT trtmnt enOTTF bsTET:
## baseToEndTx          -0.378
## treatment            -0.681  0.257
## endOfTxToFu          -0.215  0.345  0.146
## baseToEndTx:treatment  0.231 -0.611 -0.347 -0.211
## treatment:endOfTxToFu  0.133 -0.213 -0.203 -0.618  0.327
##
## Standardized Within-Group Residuals:
##         Min          Q1         Med          Q3         Max
## -2.78637452 -0.53144971 -0.01656472  0.52933002  3.33693766
##
## Number of Observations: 395
## Number of Groups: 74

# model6 is best fitting model with predictors of interest
model8 <- lme(painInterferenceMean ~ baseHamd + baseToEndTx*treatment + endOfTxToFu*treatment,
              data = bpiDat,
              method = "REML",
              na.action = "na.exclude",
              random = ~baseToEndTx | id)

summary(model8)
## Linear mixed-effects model fit by REML
##   Data: bpiDat
##       AIC      BIC    logLik
##   1418.78 1461.619 -698.3901
##
## Random effects:
##  Formula: ~baseToEndTx | id
##  Structure: General positive-definite, Log-Cholesky parametrization
##             StdDev    Corr
## (Intercept) 1.5869562 (Intr)
## baseToEndTx 0.1175798 0.078
## Residual    1.2220191
##
## Fixed effects:  painInterferenceMean ~ baseHamd + baseToEndTx * treatment + endOfTxToFu *      treat
```

```
##                            Value Std.Error  DF   t-value p-value
## (Intercept)             5.150542 0.3101691 296 16.605591  0.0000
## baseHamd                0.181401 0.0294060  67  6.168829  0.0000
## baseToEndTx            -0.152557 0.0348494 296 -4.377600  0.0000
## treatment              -0.281007 0.4559717  67 -0.616283  0.5398
## endOfTxToFu            -0.026587 0.0113828 296 -2.335715  0.0202
## baseToEndTx:treatment   0.106525 0.0558261 296  1.908161  0.0573
## treatment:endOfTxToFu   0.021982 0.0177460 296  1.238708  0.2164
##  Correlation:
##                        (Intr) basHmd bsTEnT trtmnt enOTTF bsTET:
## baseHamd               -0.143
## baseToEndTx            -0.234  0.014
## treatment              -0.695  0.199  0.160
## endOfTxToFu            -0.275  0.011  0.342  0.188
## baseToEndTx:treatment   0.146 -0.009 -0.624 -0.221 -0.213
## treatment:endOfTxToFu   0.174  0.012 -0.219 -0.259 -0.641  0.328
##
## Standardized Within-Group Residuals:
##        Min         Q1        Med         Q3        Max
## -2.6990103 -0.5297913  0.0138089  0.5049364  3.3011425
##
## Number of Observations: 370
## Number of Groups: 70

#yoga only
yogaBpiDat <- bpiDat %>% filter(treatment == 0)

model11 <- lme(painInterferenceMean ~ baseHamd + baseToEndTx + endOfTxToFu,
                data = yogaBpiDat,
                method = "REML",
                random = ~baseToEndTx|id,
                na.action = "na.exclude")
summary(model11)
## Linear mixed-effects model fit by REML
##   Data: yogaBpiDat
##        AIC     BIC    logLik
##   834.5412 861.469 -409.2706
##
## Random effects:
##  Formula: ~baseToEndTx | id
##  Structure: General positive-definite, Log-Cholesky parametrization
##             StdDev     Corr
## (Intercept) 1.51089270 (Intr)
## baseToEndTx 0.07936184 -0.326
## Residual    1.27016280
##
## Fixed effects:  painInterferenceMean ~ baseHamd + baseToEndTx + endOfTxToFu
##                 Value  Std.Error  DF   t-value p-value
## (Intercept)  5.182859 0.30384986 180 17.057302  0.0000
## baseHamd     0.158166 0.03515392  34  4.499235  0.0001
## baseToEndTx -0.154427 0.03143430 180 -4.912682  0.0000
## endOfTxToFu -0.026665 0.01181215 180 -2.257428  0.0252
##  Correlation:
```

```
##              (Intr) basHmd bsTEnT
## baseHamd    -0.173
## baseToEndTx -0.454  0.033
## endOfTxToFu -0.294  0.015  0.384
##
## Standardized Within-Group Residuals:
##          Min           Q1          Med           Q3          Max
## -2.631027927 -0.549565233 -0.006220577  0.485588104  3.208212271
##
## Number of Observations: 218
## Number of Groups: 36

#cbt only
cbtBpiDat <- bpiDat %>% filter(treatment == 1)

model12 <- lme(painInterferenceMean ~ baseHamd + baseToEndTx + endOfTxToFu,
                data = cbtBpiDat,
                method = "REML",
                random = ~baseToEndTx|id,
                na.action = "na.exclude")
summary(model12)
## Linear mixed-effects model fit by REML
##   Data: cbtBpiDat
##        AIC      BIC    logLik
##   591.1252 615.1029 -287.5626
##
## Random effects:
##  Formula: ~baseToEndTx | id
##  Structure: General positive-definite, Log-Cholesky parametrization
##             StdDev    Corr
## (Intercept) 1.6955513 (Intr)
## baseToEndTx 0.1710536 0.303
## Residual    1.1385070
##
## Fixed effects:  painInterferenceMean ~ baseHamd + baseToEndTx + endOfTxToFu
##                  Value Std.Error  DF   t-value p-value
## (Intercept)   4.916970 0.3429303 116 14.338103  0.0000
## baseHamd      0.212506 0.0477758  32  4.447977  0.0001
## baseToEndTx -0.062812 0.0509146 116 -1.233682  0.2198
## endOfTxToFu -0.004515 0.0127570 116 -0.353922  0.7240
##  Correlation:
##             (Intr) basHmd bsTEnT
## baseHamd     0.218
## baseToEndTx -0.017  0.020
## endOfTxToFu -0.218  0.040  0.273
##
## Standardized Within-Group Residuals:
##        Min         Q1        Med         Q3        Max
## -2.3238572 -0.5031548 -0.0628340  0.5331349  3.0018283
##
## Number of Observations: 152
## Number of Groups: 34
```

```
#cohen's d
painIntCohenDat <- bpiDat %>%
        group_by(treatment, week) %>%
        summarise(meanPainInt = mean(painInterferenceMean, na.rm = T)) %>%
        ungroup() %>%
        group_by(week) %>%
        mutate(meanDif = last(meanPainInt) - first(meanPainInt)) %>%
        ungroup() %>%
        mutate(painIntCohenD = purrr::map_dbl(meanDif, nlmeCohenD, model8)) %>%
        filter(week >= 10) %>%
        select(treatment, week, meanPainInt, painIntCohenD)
## `summarise()` has grouped output by 'treatment'. You can override using the
## `.groups` argument.

painIntFig <- plot(ggpredict(model8, c("endOfTxToFu", "treatment"), ci.lvl = NA), use.theme = F,
     connect.lines = TRUE, colors = "bw") +
        ylim(3,6) +
        geom_point() +
        theme(axis.text=element_text(size = 18),
                                            axis.title=element_text(size = 18, face="bold"),
                                            strip.text.x = element_text(size = 18)) +
        scale_color_manual(name = "",
                           labels = c("Yoga", "CBT"),
                           values = c("#000000", "#000000"),
                           guide = guide_legend(reverse = F)) +
        scale_linetype_manual(name = "",
                           labels = c("Yoga", "CBT"),
                           values = c("dashed", "solid"),
                           guide = guide_legend(reverse = F)) +
        jtools::theme_apa(legend.pos = "top", x.font.size = 18, y.font.size = 18,
                          facet.title.size = 18, legend.use.title = F) +
        scale_x_continuous(breaks=c(0, 8, 16, 24),
                        labels=c("EOT", "2-mos", "4-mos", "6-mos")) +
        theme(axis.text.x = element_text(size = 18),
              axis.text.y = element_text(size = 18),
              panel.border = element_blank()) +
        labs(tag = "B", title = "", size = 18) +
        xlab("") + ylab("BPI-SF Pain Interference") +
        theme(axis.line = element_line(color = "black")) +
        theme(legend.text=element_text(size=18))
## Scale for y is already present.
## Adding another scale for y, which will replace the existing scale.
## Scale for colour is already present.
## Adding another scale for colour, which will replace the existing scale.
```

```
plot(ggpredict(model8, c("baseToEndTx", "treatment"), ci.lvl = NA), use.theme = F) +
                                        scale_x_continuous(breaks = c(0, 2, 4, 6, 8, 10)) +
                                        theme(axis.text=element_text(size = 14),
                                              axis.title=element_text(size = 16,face="bold"),
                                              strip.text.x = element_text(size = 16)) +
        ylim(3,6) +
        geom_point() +
        scale_colour_brewer(palette = "Set1", labels = c("yoga", "cbt"))
```

```
## Scale for y is already present.
## Adding another scale for y, which will replace the existing scale.
## Scale for colour is already present.
## Adding another scale for colour, which will replace the existing scale.
```

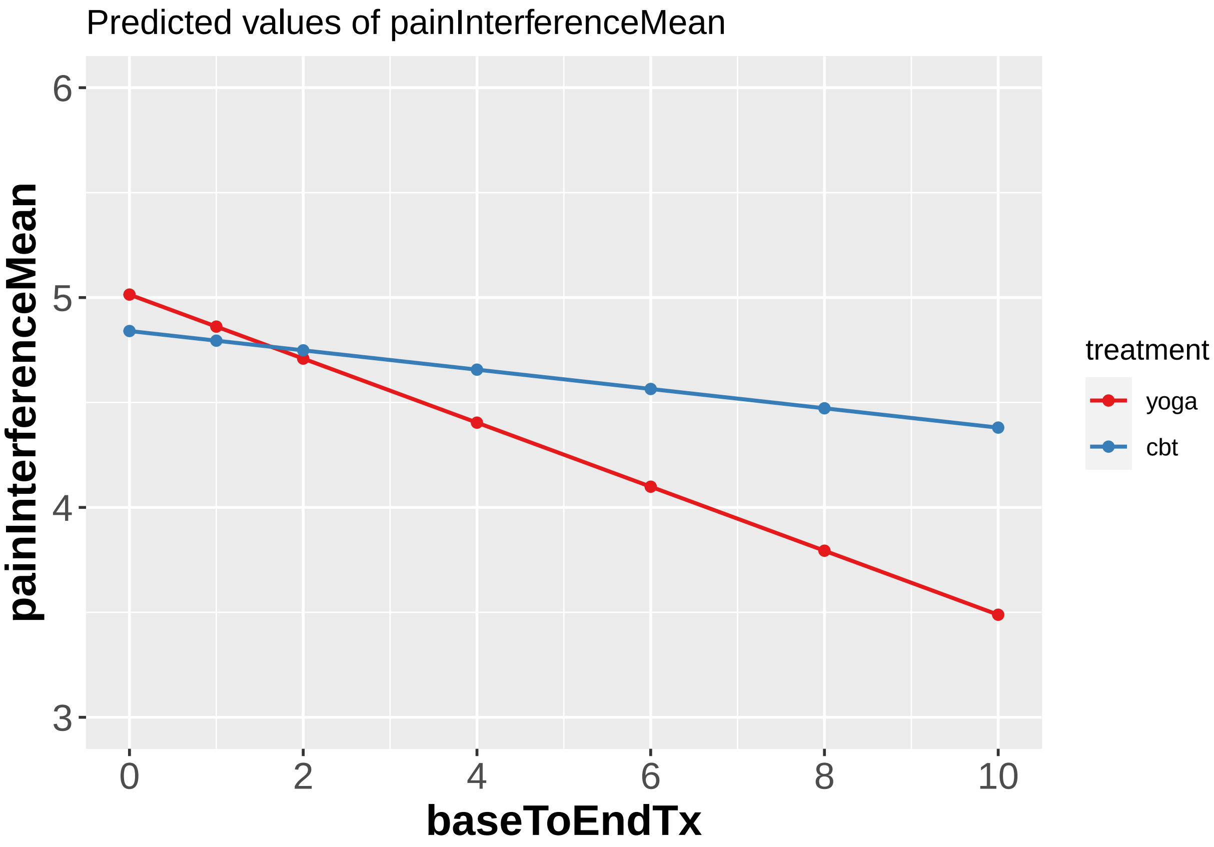


```
    predictedDat <- bpiDat %>% na.omit(bpiDat) %>% modelr::add_predictions(model8, type = "response
```

Check Assumptions: Model 8

```
#check for independence and normality of within-group errors (participants)
plot(model8, resid(., type="p") ~ fitted(.), abline=0)
```

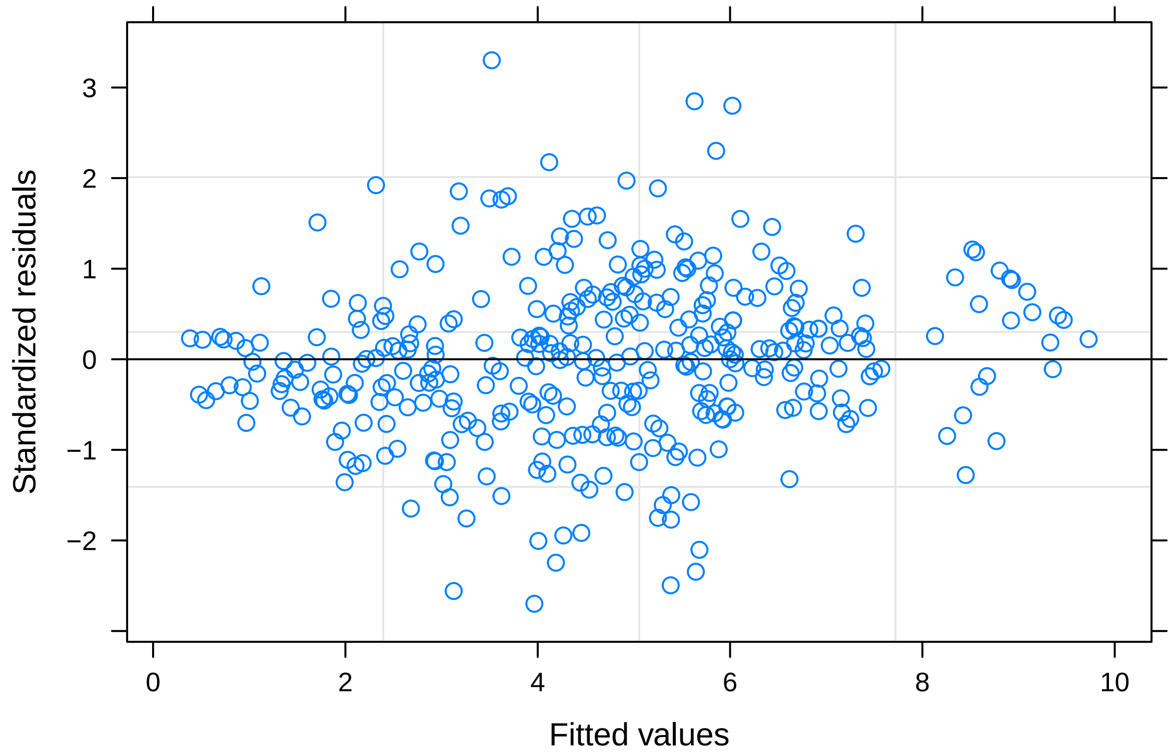


```r
plot(model8, id~resid(.), abline = 0 )
```

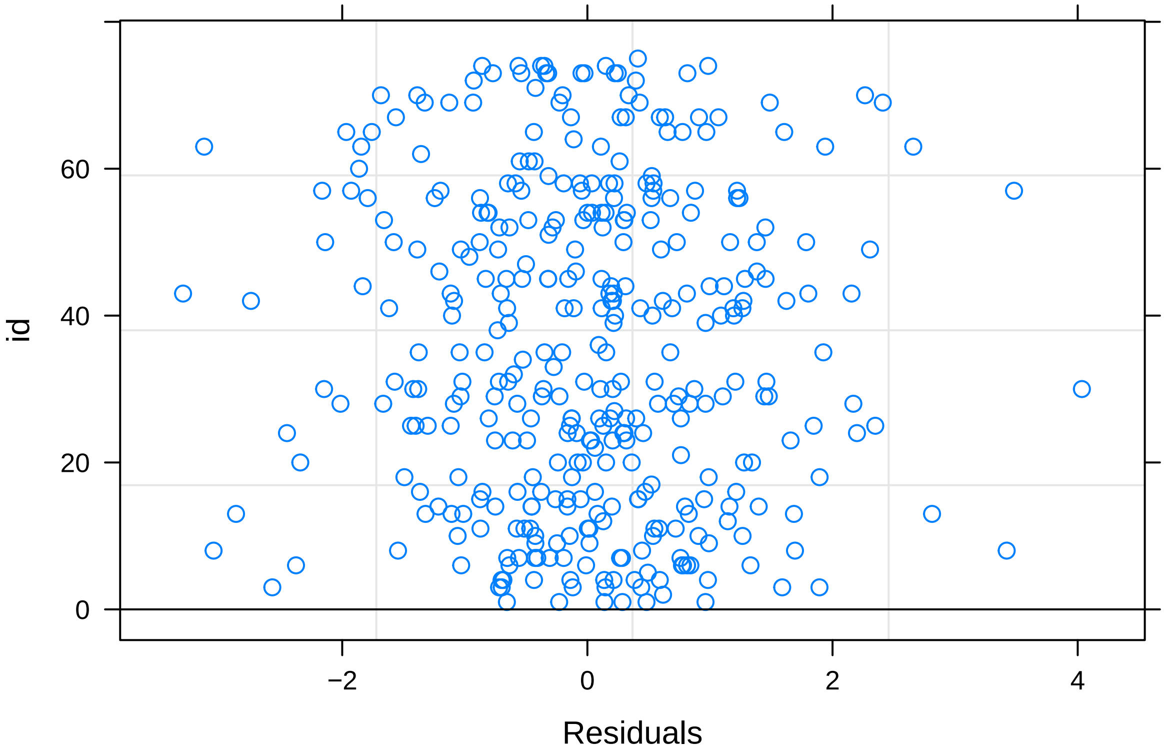


```r
plot(model8, resid(., type = "p") ~ fitted(.) | week, id = 0.05, adj = -0.3 )
```

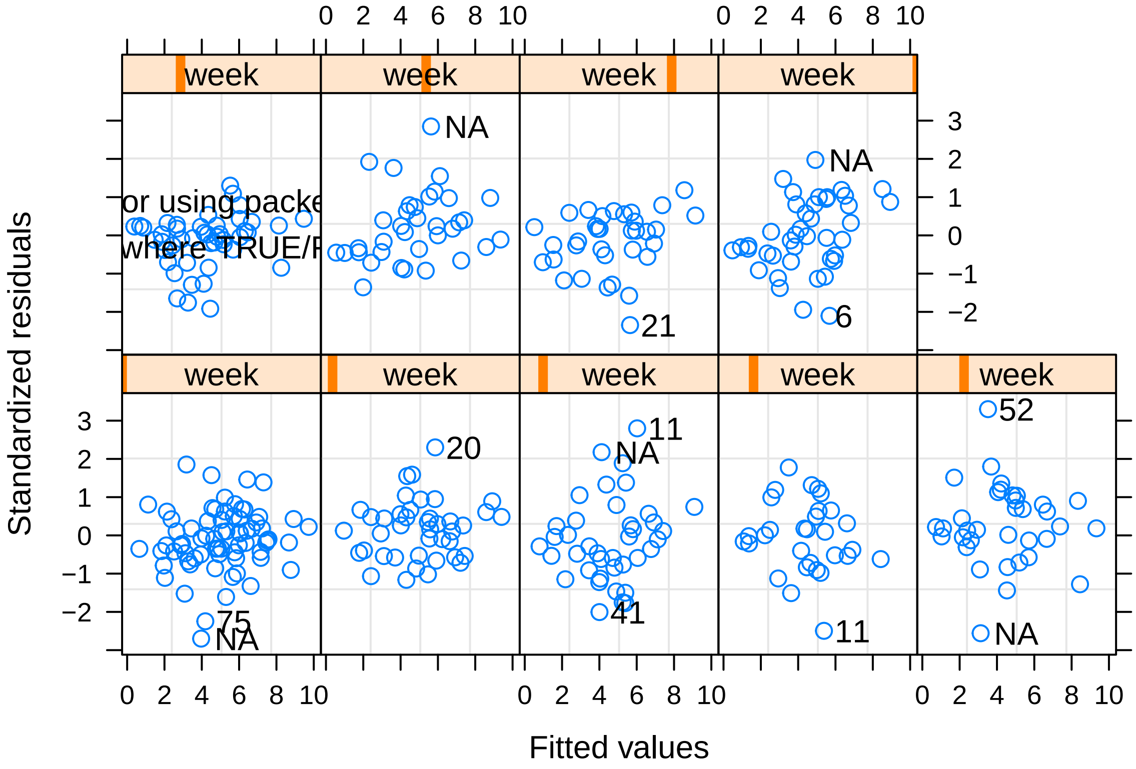


```
plot(model8, painInterferenceMean ~ fitted(.) | week, id = 0.05, adj = -0.3 )
```

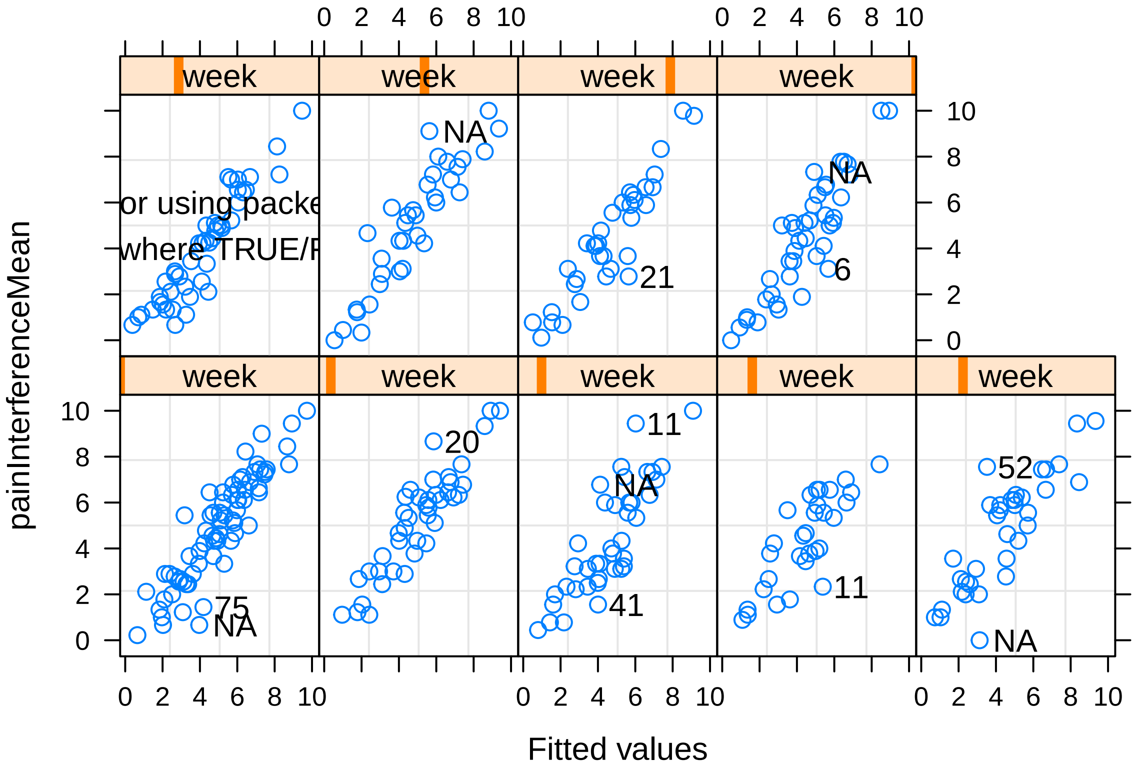

```
plot(model8, painInterferenceMean ~ fitted(.), id=.05, adj=-0.3)
```

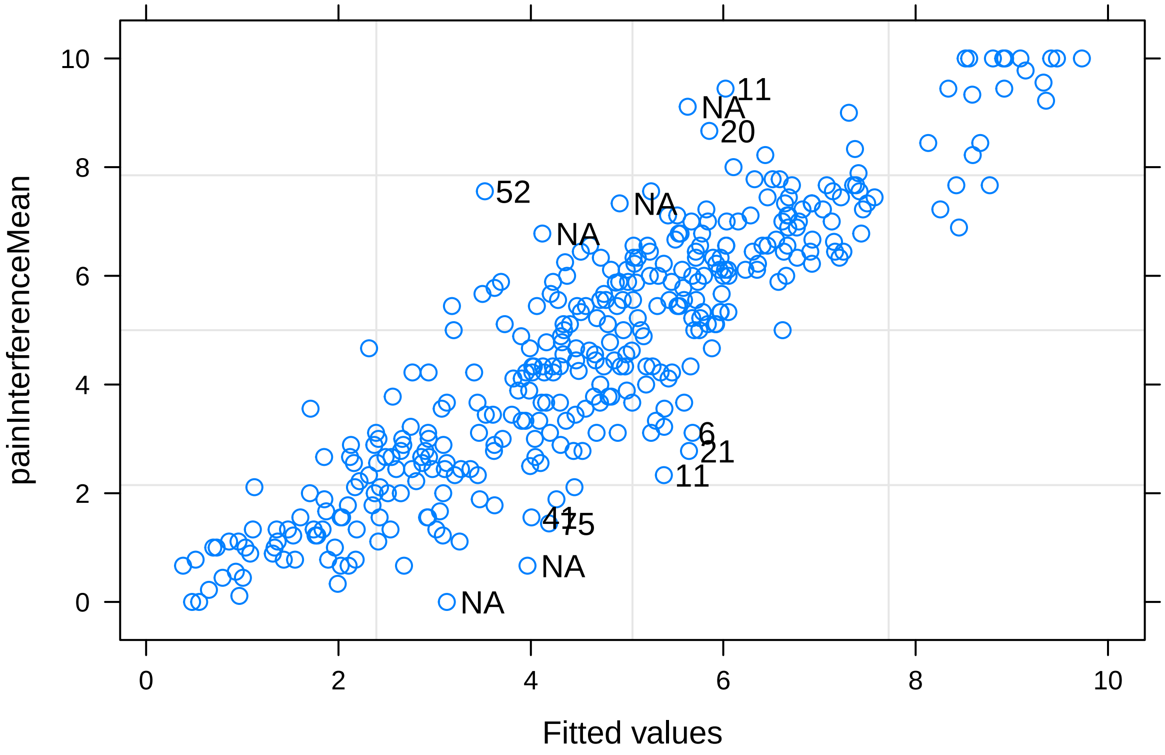


```
hist(residuals(model8), breaks=20, id=.05)
## Warning in plot.window(xlim, ylim, "", ...): "id" is not a graphical parameter
## Warning in title(main = main, sub = sub, xlab = xlab, ylab = ylab, ...): "id"
## is not a graphical parameter
## Warning in axis(1, ...): "id" is not a graphical parameter
## Warning in axis(2, at = yt, ...): "id" is not a graphical parameter
```

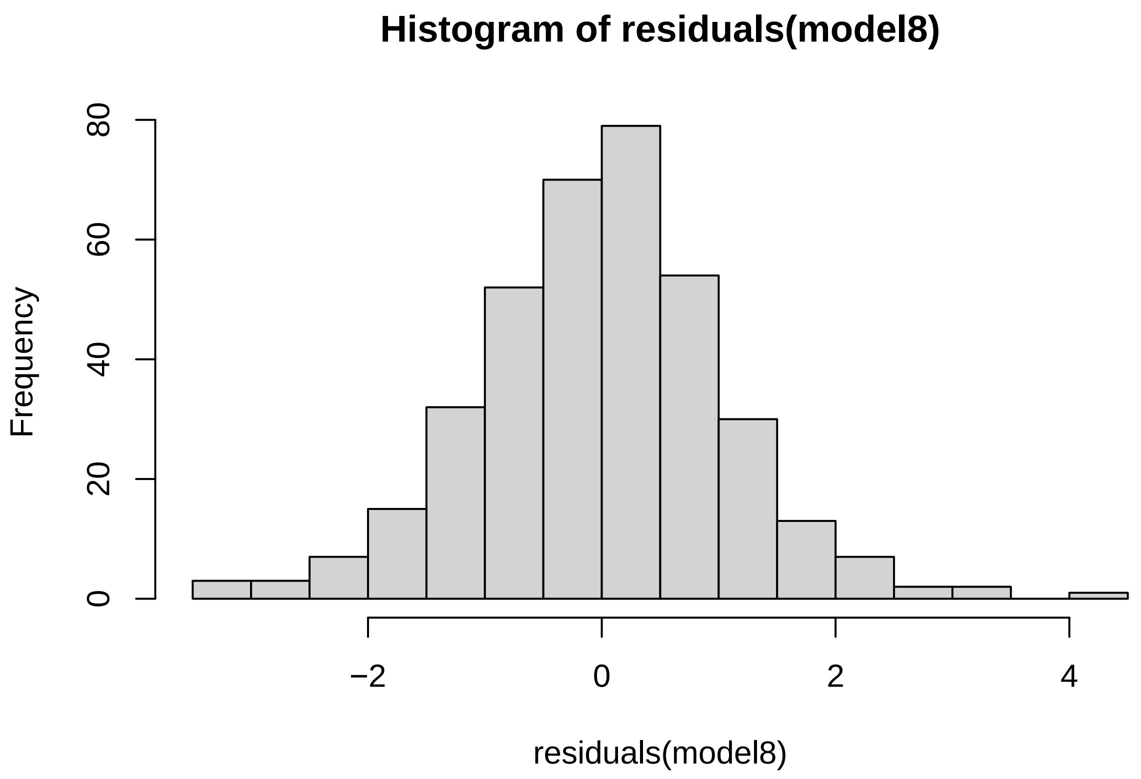


```
qqnorm(model8)
```

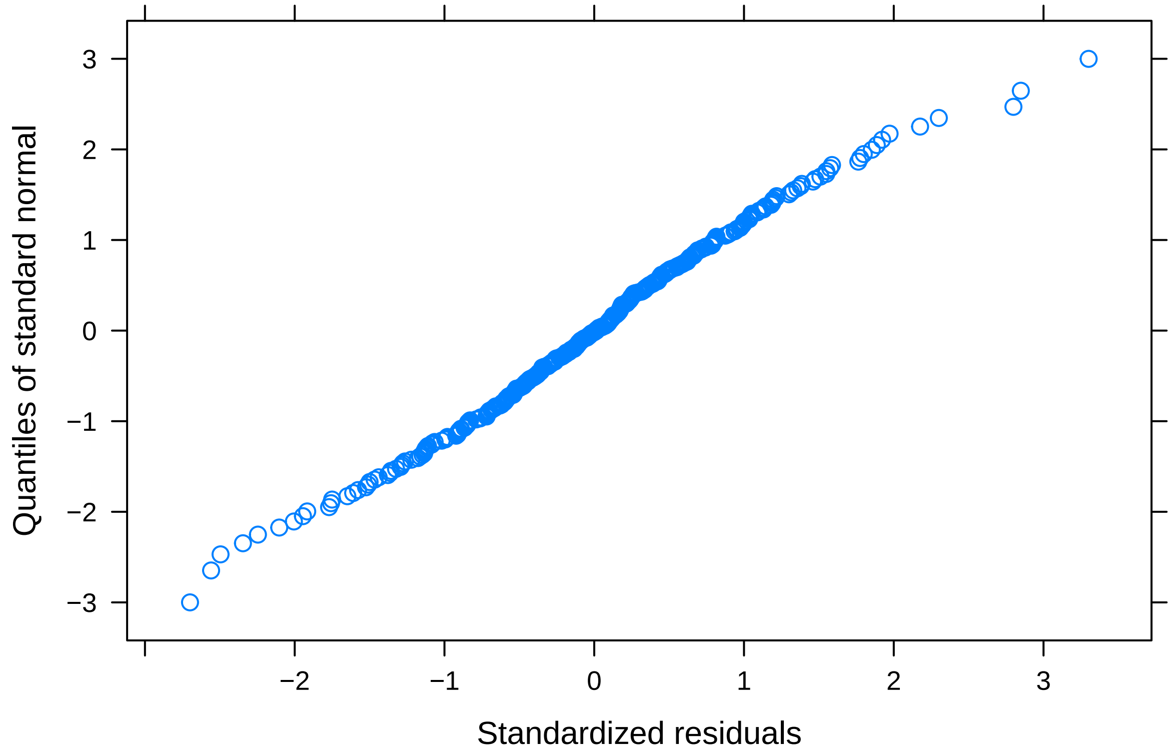

```r
#check for normality and independence of random effects
qqnorm(model8, ~ranef(.), id=0.10, cex=0.7)
```

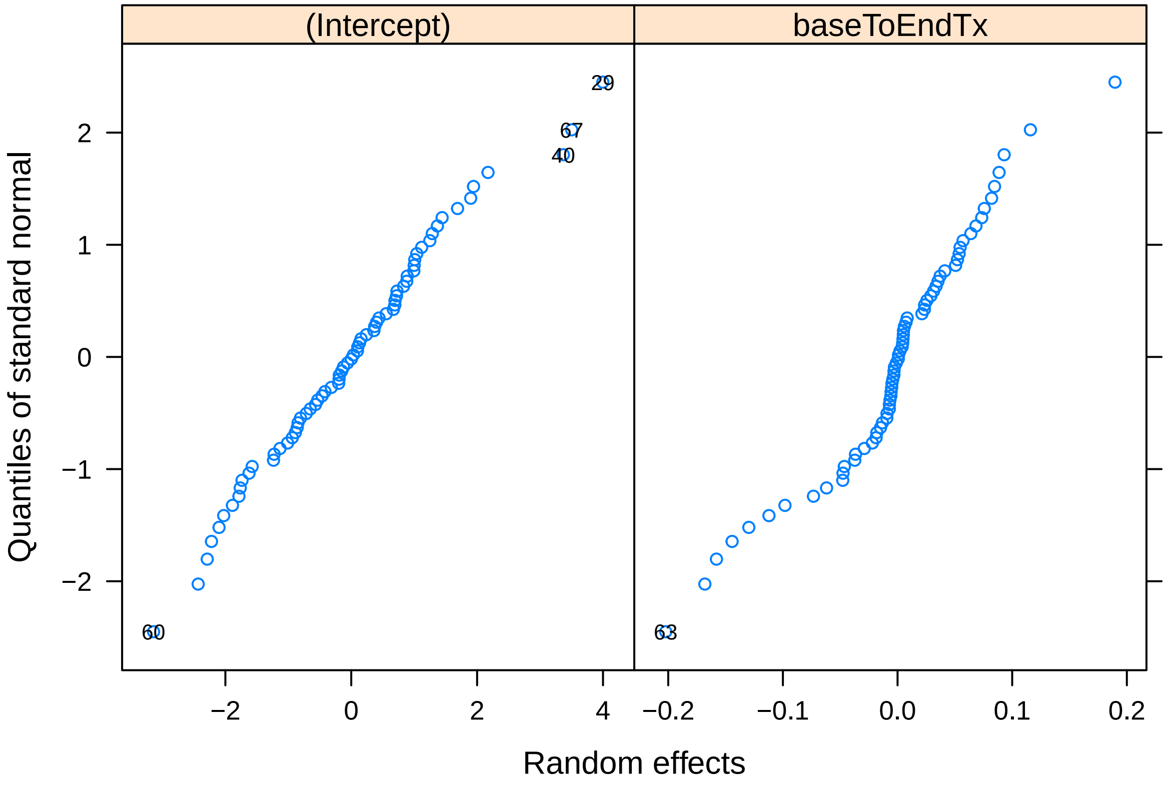


```r
pairs(model8, ~ranef(., level=1), adj=-0.3)
```

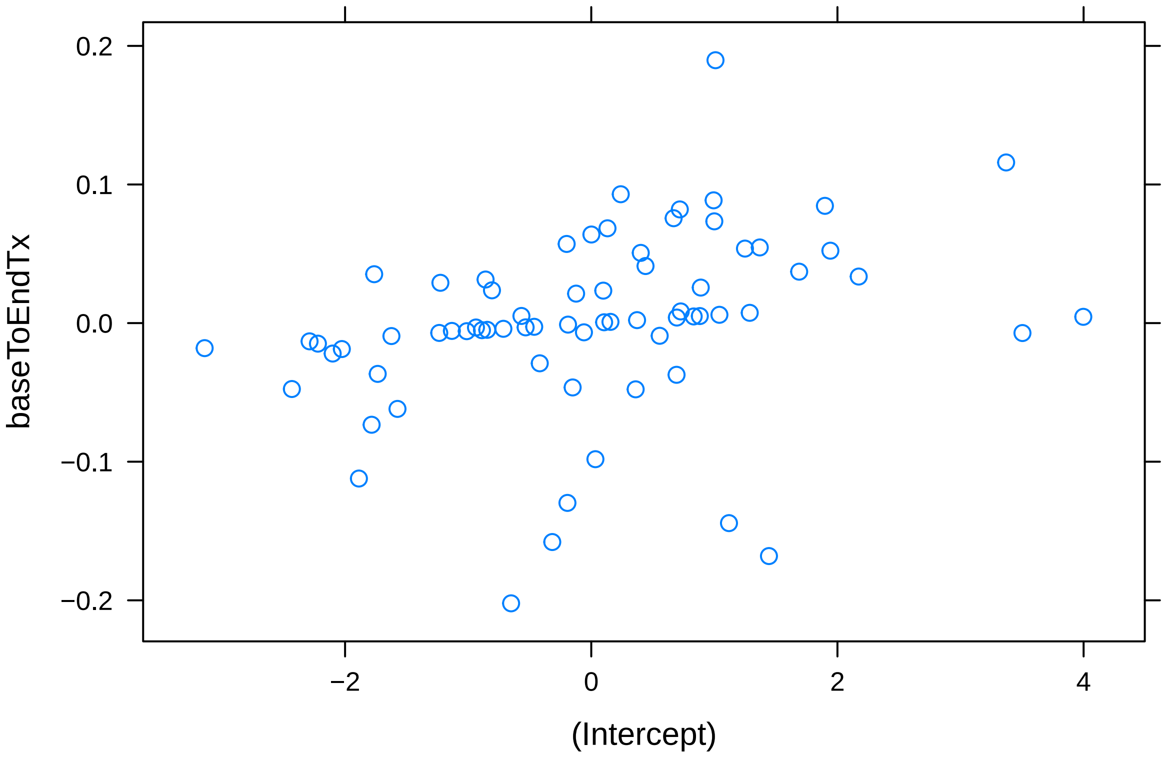

Check Assumptions: Model 9

```
#check for independence and normality of within-group errors (participants)
plot(model6, resid(., type="p") ~ fitted(.), abline=0)
```

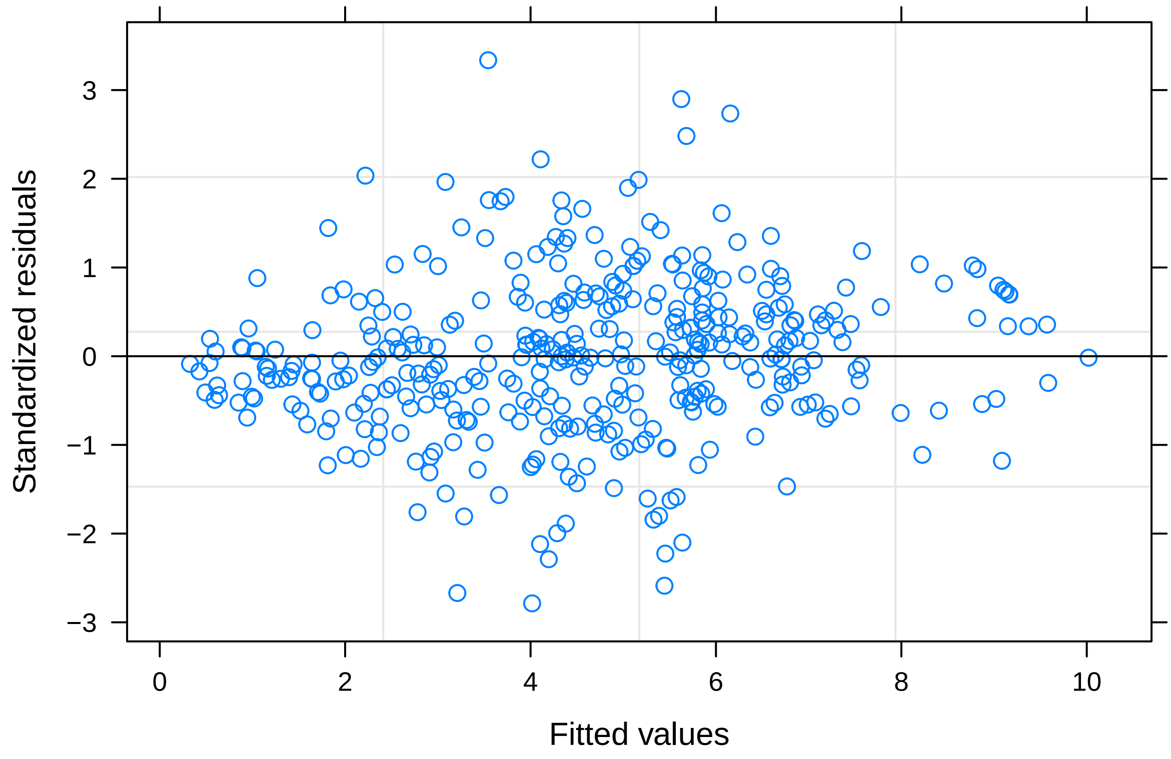


```
plot(model6, id~resid(.), abline = 0 )
```

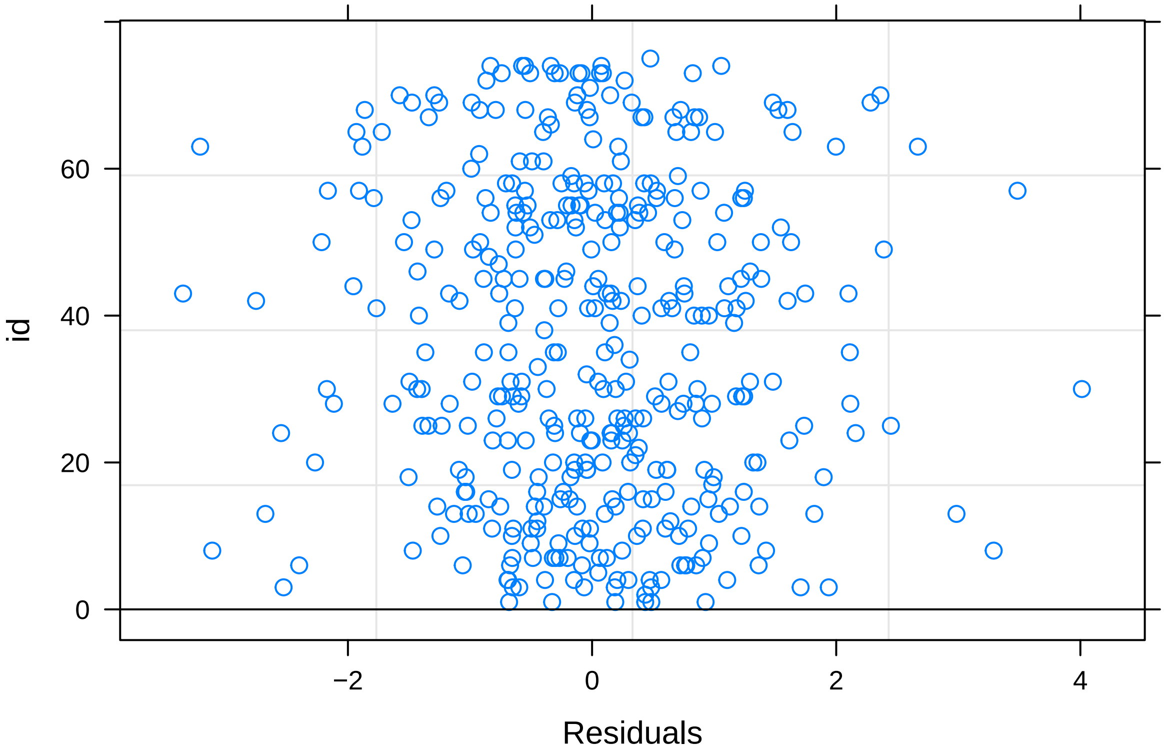


```
plot(model6, resid(., type = "p") ~ fitted(.) | week, id = 0.05, adj = -0.3 )
```

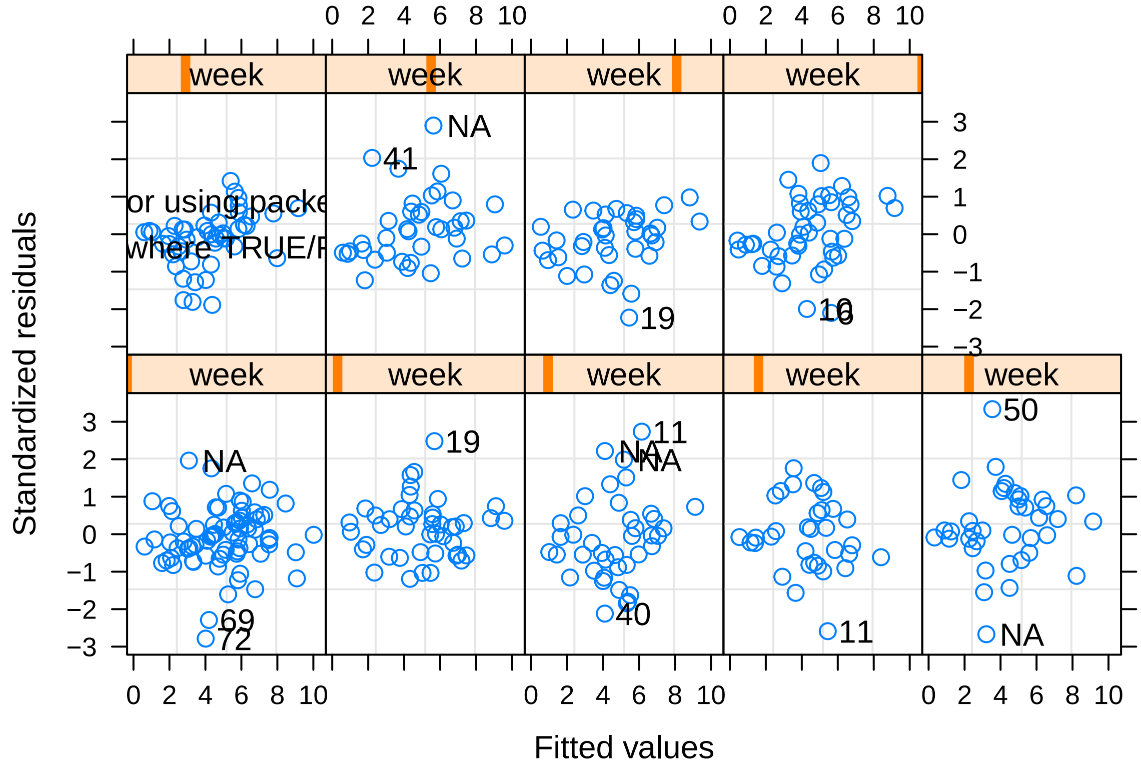


```
plot(model6, painInterferenceMean ~ fitted(.) | week, id = 0.05, adj = -0.3 )
```

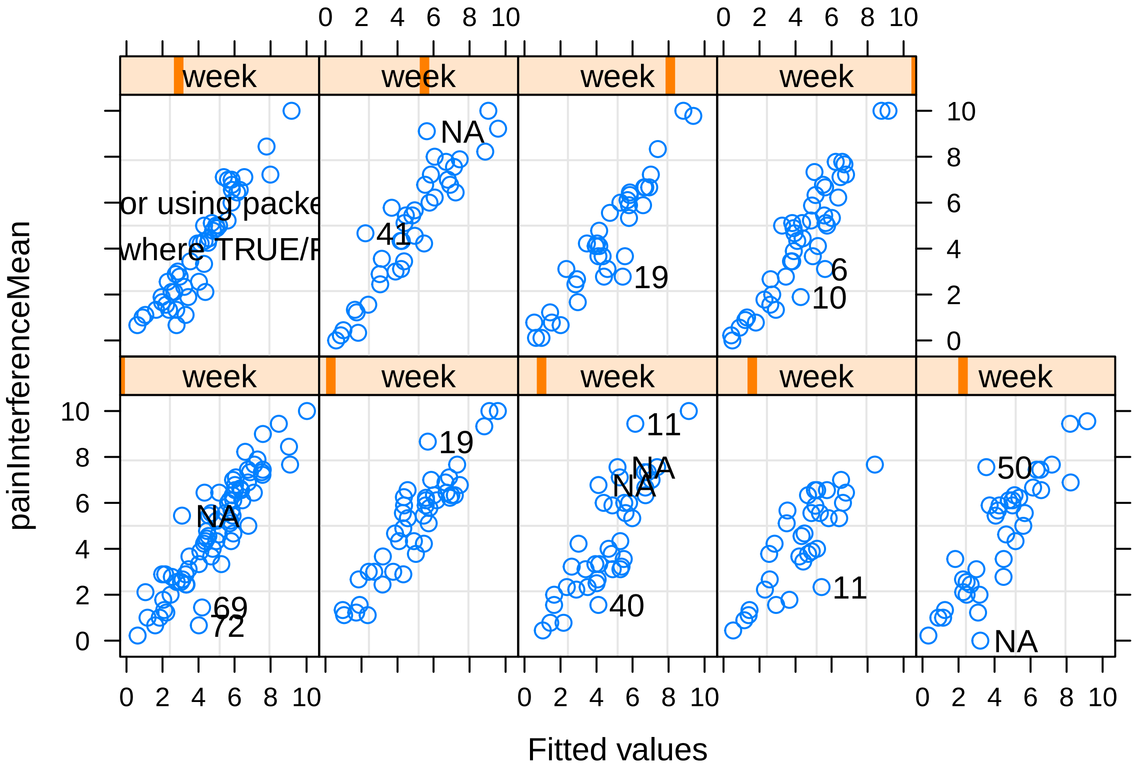

```r
plot(model6, painInterferenceMean ~ fitted(.), id=.05, adj=-0.3)
```

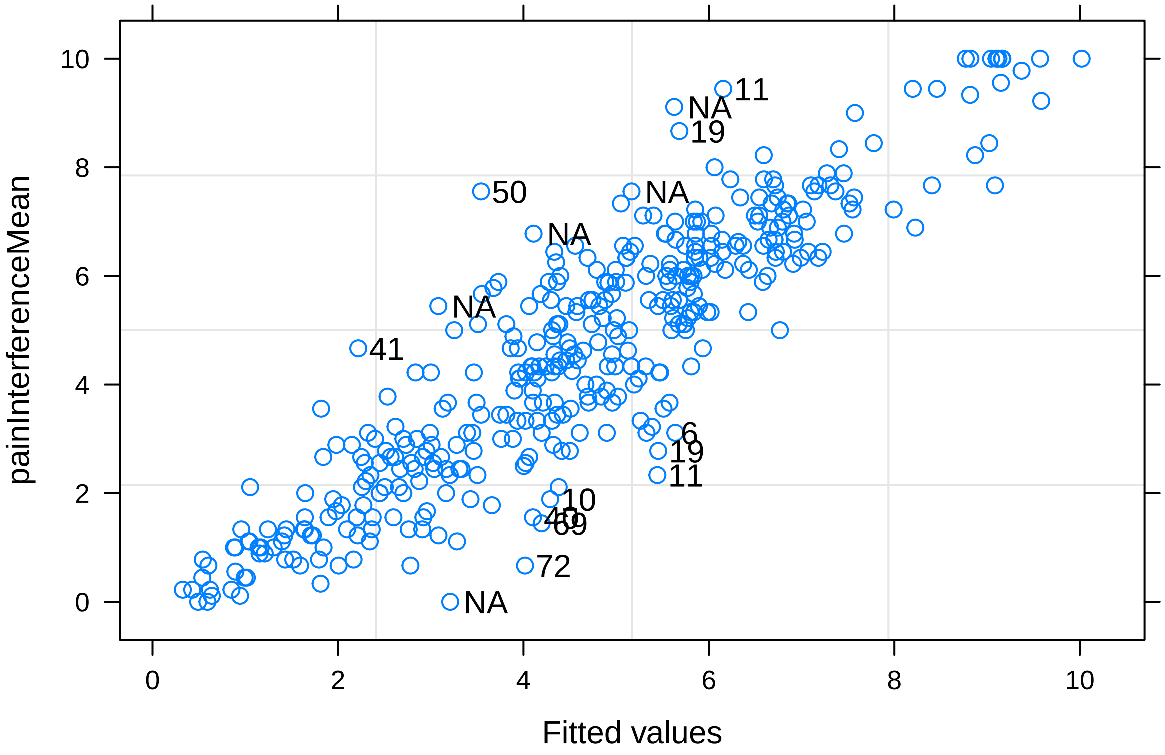


```r
hist(residuals(model6), breaks=20, id=.05)
## Warning in plot.window(xlim, ylim, "", ...): "id" is not a graphical parameter
## Warning in title(main = main, sub = sub, xlab = xlab, ylab = ylab, ...): "id"
## is not a graphical parameter
## Warning in axis(1, ...): "id" is not a graphical parameter
## Warning in axis(2, at = yt, ...): "id" is not a graphical parameter
```

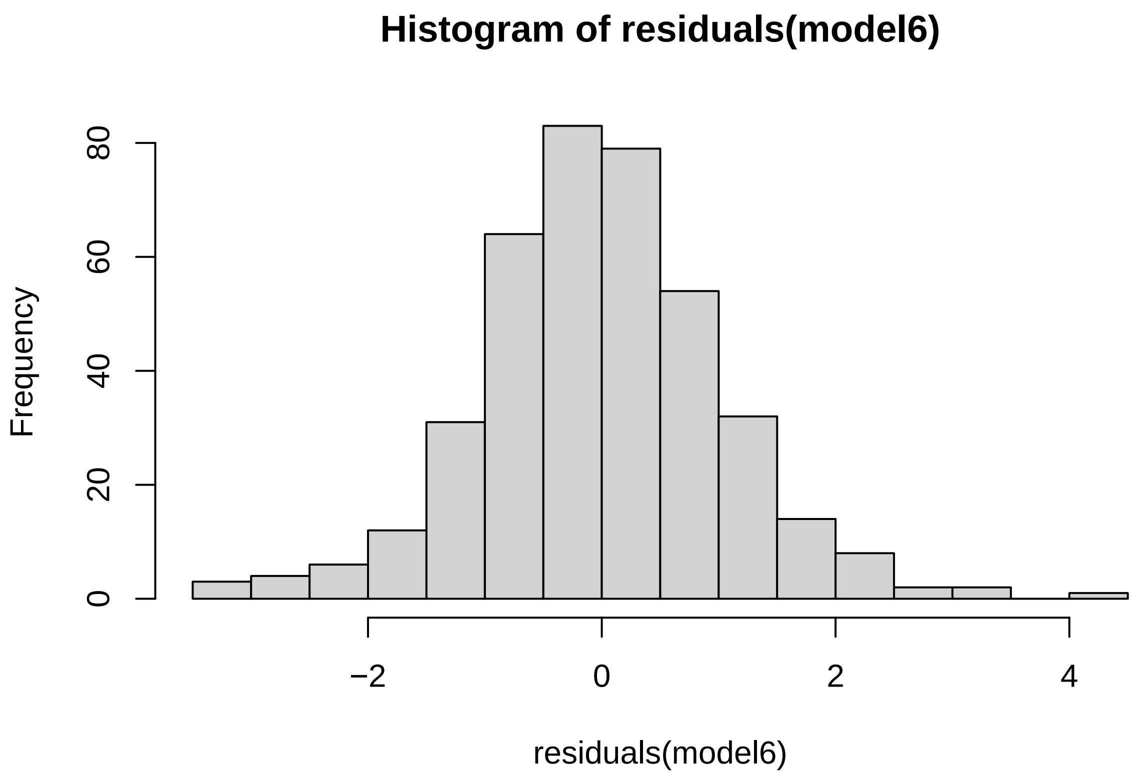


```
qqnorm(model6)
```

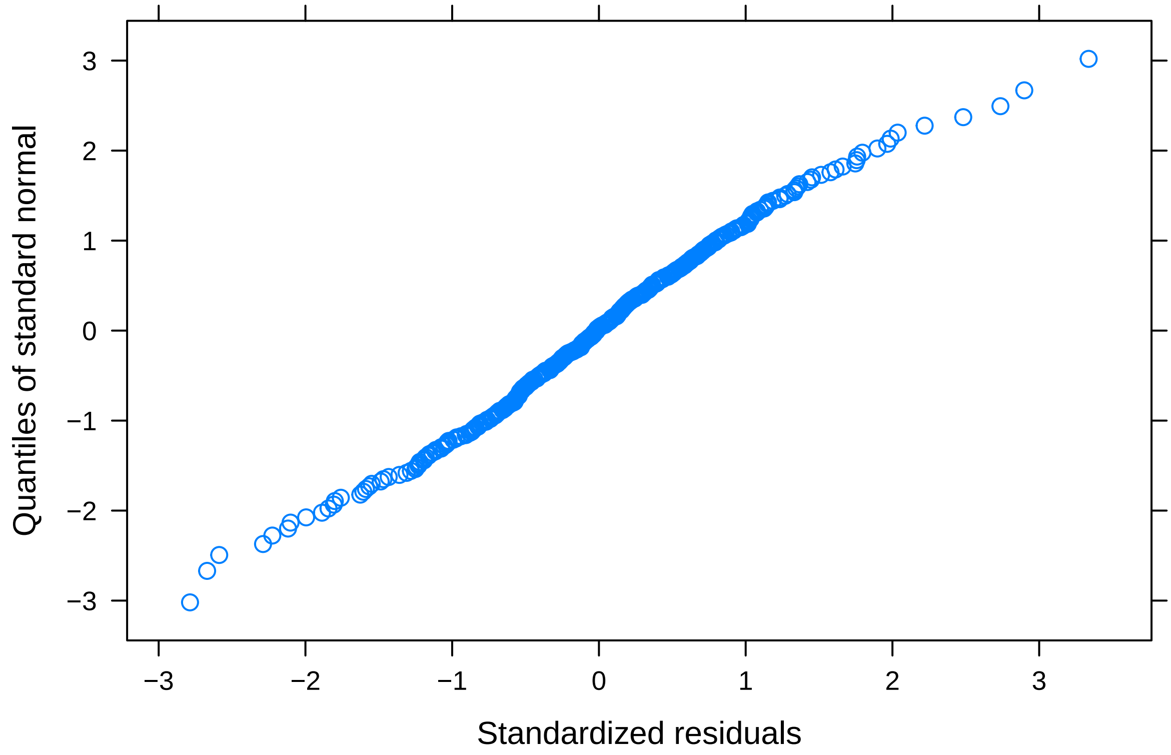

```
#check for normality and independence of random effects
qqnorm(model6, ~ranef(.), id=0.10, cex=0.7)
```

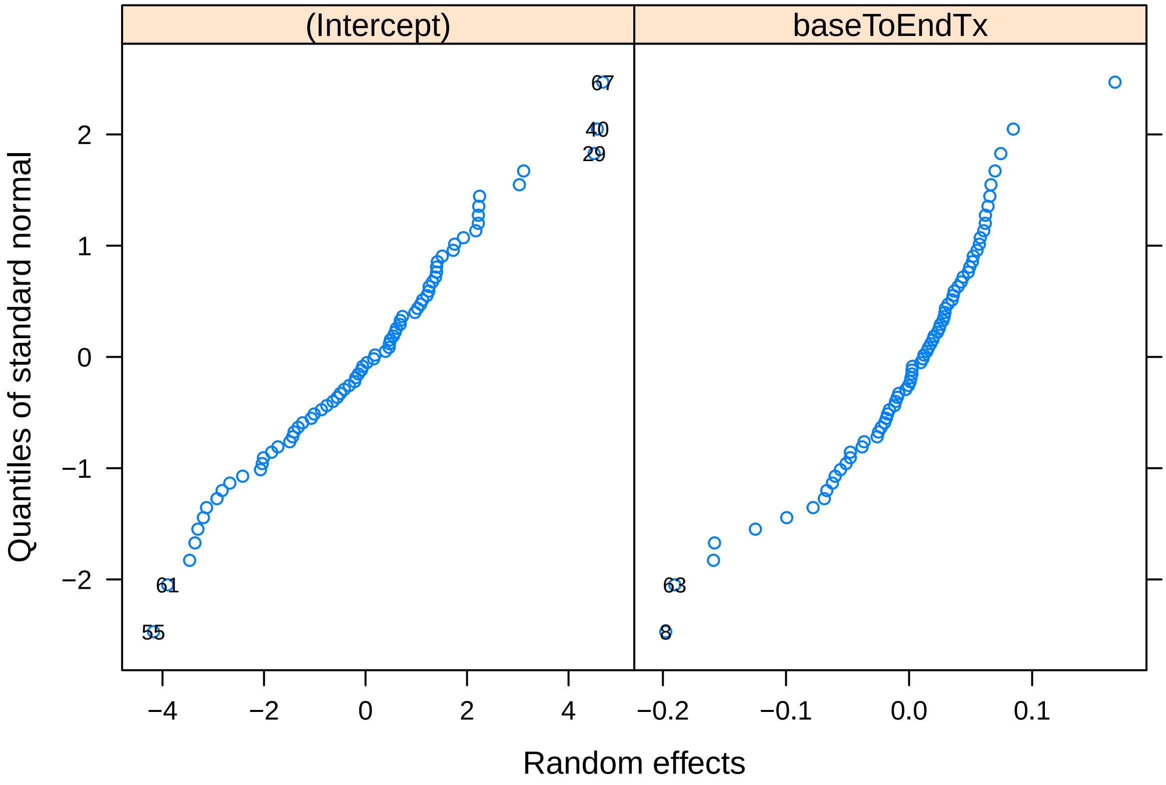


```
pairs(model6, ~ranef(., level=1), adj=-0.3)
```

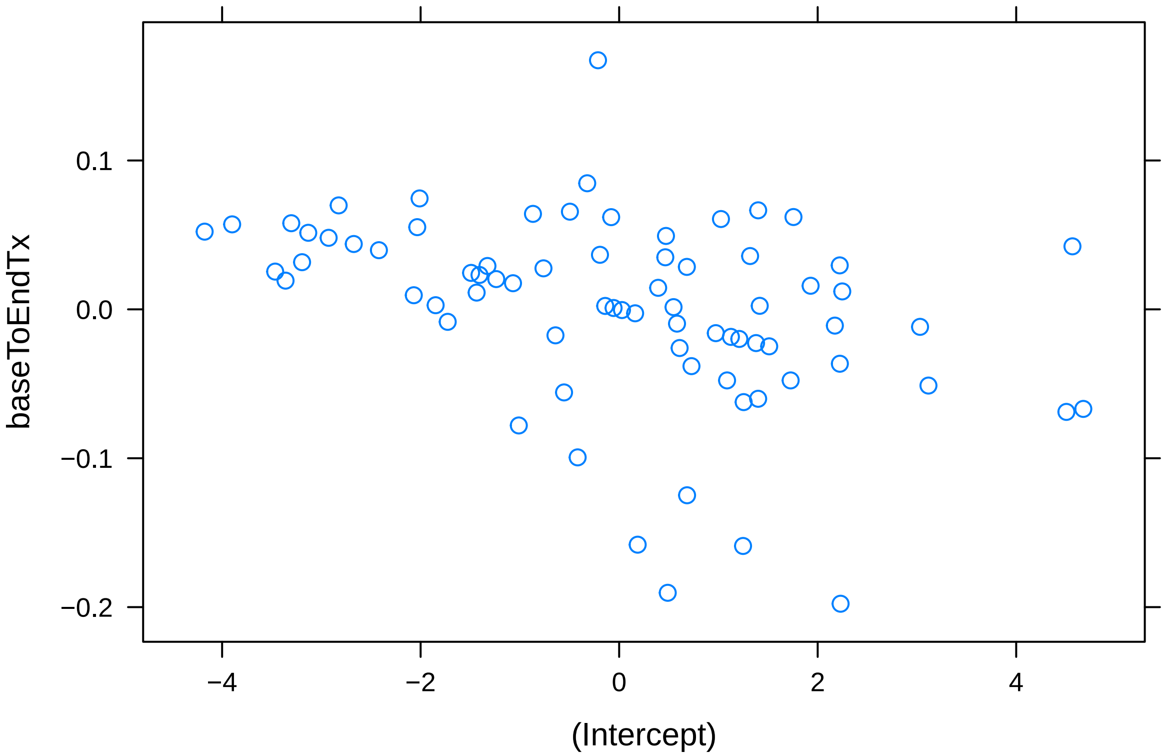

Pain Total Modeling Procedure

```
###build models: step-up procedure
#empty intercept-only model
model1 <- gls(bpiTotal ~ 1,
                  data = bpiDat,
                  method = "REML",
                  na.action = "na.exclude")
summary(model1)
## Generalized least squares fit by REML
##   Model: bpiTotal ~ 1
##   Data: bpiDat
##        AIC      BIC    logLik
##   1748.669 1756.621 -872.3344
##
## Coefficients:
##                Value Std.Error  t-value p-value
## (Intercept) 4.759737 0.1105904 43.03934       0
##
## Standardized residuals:
##         Min          Q1         Med          Q3         Max
## -2.13054651 -0.76563227  0.03931715  0.70427537  2.38416981
##
## Residual standard error: 2.19794
## Degrees of freedom: 395 total; 394 residual

#random intercept model
```

```r
model2 <- lme(bpiTotal ~ 1,
                 data = bpiDat,
                 method = "REML",
                 random = ~1|id,
                 na.action = "na.exclude")
summary(model2)
## Linear mixed-effects model fit by REML
##   Data: bpiDat
##        AIC      BIC    logLik
##   1420.733 1432.662 -707.3664
##
## Random effects:
##  Formula: ~1 | id
##         (Intercept) Residual
## StdDev:    1.874345 1.150102
##
## Fixed effects:  bpiTotal ~ 1
##                Value Std.Error  DF  t-value p-value
## (Intercept) 4.739172 0.2318627 321 20.43957       0
##
## Standardized Within-Group Residuals:
##         Min          Q1         Med          Q3         Max
## -3.26010878 -0.50377645 -0.02586891  0.52295271  3.34535994
##
## Number of Observations: 395
## Number of Groups: 74
anova(model1, model2)
##        Model df      AIC      BIC    logLik   Test L.Ratio p-value
## model1     1  2 1748.669 1756.621 -872.3344
## model2     2  3 1420.733 1432.662 -707.3664 1 vs 2 329.936  <.0001
icc(model2)
## [1] 0.73

#add fixed effects for time, fixed linear time model
model3 <- lme(bpiTotal ~ baseToEndTx + endOfTxToFu,
                 data = bpiDat,
                 method = "REML",
                 random = ~1|id,
                 na.action = "na.exclude")
summary(model3)
## Linear mixed-effects model fit by REML
##   Data: bpiDat
##        AIC      BIC    logLik
##   1412.556 1432.412 -701.2778
##
## Random effects:
##  Formula: ~1 | id
##         (Intercept) Residual
## StdDev:    1.901035 1.105117
##
## Fixed effects:  bpiTotal ~ baseToEndTx + endOfTxToFu
##                 Value  Std.Error  DF   t-value p-value
## (Intercept)  5.078799 0.24400722 319 20.814134  0.0000
```

```
## baseToEndTx -0.097467 0.01858946 319 -5.243112  0.0000
## endOfTxToFu -0.018366 0.00763353 319 -2.406016  0.0167
##  Correlation:
##             (Intr) bsTEnT
## baseToEndTx -0.263
## endOfTxToFu -0.214  0.438
##
## Standardized Within-Group Residuals:
##          Min           Q1          Med           Q3          Max
## -3.032190911 -0.520201666 -0.007941723  0.532909208  3.227137353
##
## Number of Observations: 395
## Number of Groups: 74


#add random effect for endOfTxToFu, random linear time model
model4 <- lme(bpiTotal ~ baseToEndTx + endOfTxToFu,
                 data = bpiDat,
                 method = "REML",
                 random = ~endOfTxToFu|id,
                 na.action = "na.exclude")
summary(model4)
## Linear mixed-effects model fit by REML
##   Data: bpiDat
##        AIC    BIC    logLik
##   1412.801 1440.6 -699.4003
##
## Random effects:
##  Formula: ~endOfTxToFu | id
##  Structure: General positive-definite, Log-Cholesky parametrization
##             StdDev     Corr
## (Intercept) 1.83761436 (Intr)
## endOfTxToFu 0.01691568 0.807
## Residual    1.09653652
##
## Fixed effects:  bpiTotal ~ baseToEndTx + endOfTxToFu
##                 Value  Std.Error  DF   t-value p-value
## (Intercept)  5.079509 0.23696792 319 21.435429  0.0000
## baseToEndTx -0.096099 0.01842690 319 -5.215128  0.0000
## endOfTxToFu -0.018753 0.00803026 319 -2.335335  0.0201
##  Correlation:
##             (Intr) bsTEnT
## baseToEndTx -0.270
## endOfTxToFu -0.021  0.430
##
## Standardized Within-Group Residuals:
##         Min          Q1         Med          Q3         Max
## -3.06349872 -0.49463742 -0.01864136  0.54780860  3.28065885
##
## Number of Observations: 395
## Number of Groups: 74
anova(model3, model4) # not a better fit
##        Model df      AIC      BIC    logLik   Test  L.Ratio p-value
```

```
## model3     1  5 1412.556 1432.412 -701.2778
## model4     2  7 1412.801 1440.600 -699.4003 1 vs 2 3.754889   0.153

#add random effect for baseToEndTx, random linear time model --best fitting base model--
model5 <- lme(bpiTotal ~ baseToEndTx + endOfTxToFu,
                 data = bpiDat,
                 method = "REML",
                 random = ~baseToEndTx|id,
                 na.action = "na.exclude")
summary(model5)
## Linear mixed-effects model fit by REML
##   Data: bpiDat
##        AIC      BIC    logLik
##   1408.409 1436.208 -697.2045
##
## Random effects:
##  Formula: ~baseToEndTx | id
##  Structure: General positive-definite, Log-Cholesky parametrization
##             StdDev    Corr
## (Intercept) 1.9237472 (Intr)
## baseToEndTx 0.1060605 -0.143
## Residual    1.0393128
##
## Fixed effects:  bpiTotal ~ baseToEndTx + endOfTxToFu
##                  Value  Std.Error  DF   t-value p-value
## (Intercept)  5.080971 0.24399090 319 20.824429  0.0000
## baseToEndTx -0.097294 0.02291514 319 -4.245854  0.0000
## endOfTxToFu -0.018001 0.00722273 319 -2.492252  0.0132
##  Correlation:
##             (Intr) bsTEnT
## baseToEndTx -0.264
## endOfTxToFu -0.199  0.327
##
## Standardized Within-Group Residuals:
##         Min          Q1         Med          Q3         Max
## -2.78652303 -0.52408215 -0.01121301  0.52793436  3.27717536
##
## Number of Observations: 395
## Number of Groups: 74
anova(model3, model5) # better fit
##        Model df      AIC      BIC    logLik   Test  L.Ratio p-value
## model3     1  5 1412.556 1432.412 -701.2778
## model5     2  7 1408.409 1436.208 -697.2045 1 vs 2 8.146591   0.017

#add random effect for both endOfTxToFu and baseToEndTx, random linear time model
model6 <- lme(bpiTotal ~ baseToEndTx + endOfTxToFu,
                 data = bpiDat,
                 method = "REML",
                 random = ~baseToEndTx + endOfTxToFu | id,
                 na.action = "na.exclude")
summary(model6)
## Linear mixed-effects model fit by REML
##   Data: bpiDat
```

```
##        AIC      BIC    logLik
##   1406.339 1446.051 -693.1693
##
## Random effects:
##  Formula: ~baseToEndTx + endOfTxToFu | id
##  Structure: General positive-definite, Log-Cholesky parametrization
##             StdDev     Corr
## (Intercept) 1.83784500 (Intr) bsTEnT
## baseToEndTx 0.12680911 -0.107
## endOfTxToFu 0.03451247  0.296  0.684
## Residual    1.00469813
##
## Fixed effects:  bpiTotal ~ baseToEndTx + endOfTxToFu
##                  Value  Std.Error  DF   t-value p-value
## (Intercept)  5.081242 0.23356801 319 21.754870  0.0000
## baseToEndTx -0.096816 0.02441343 319 -3.965685  0.0001
## endOfTxToFu -0.018718 0.00856814 319 -2.184576  0.0296
##  Correlation:
##             (Intr) bsTEnT
## baseToEndTx -0.237
## endOfTxToFu -0.034  0.525
##
## Standardized Within-Group Residuals:
##         Min          Q1         Med          Q3         Max
## -2.77308038 -0.49936739 -0.03047945  0.51943282  3.36951089
##
## Number of Observations: 395
## Number of Groups: 74
anova(model4, model6) # better fit
##        Model df      AIC      BIC    logLik   Test  L.Ratio p-value
## model4     1  7 1412.801 1440.600 -699.4003
## model6     2 10 1406.339 1446.051 -693.1693 1 vs 2 12.46217   0.006

#test model6 with lmer package for convergence
mod6.5 <- lme4::lmer(bpiTotal ~ baseToEndTx + endOfTxToFu + (baseToEndTx + endOfTxToFu | id),
               data = bpiDat)
## Warning in checkConv(attr(opt, "derivs"), opt$par, ctrl = control$checkConv, :
## Model failed to converge with max|grad| = 0.0027241 (tol = 0.002, component 1)

# model5 is best fitting model with predictors of interest
model7 <- lme(bpiTotal ~ baseHamd + baseToEndTx*treatment + endOfTxToFu*treatment,
                 data = bpiDat,
                 method = "REML",
                 random = ~baseToEndTx|id,
                 na.action = "na.exclude")
summary(model7)
## Linear mixed-effects model fit by REML
##   Data: bpiDat
##        AIC      BIC    logLik
##   1318.377 1361.215 -648.1883
##
## Random effects:
##  Formula: ~baseToEndTx | id
```

```
##  Structure: General positive-definite, Log-Cholesky parametrization
##             StdDev   Corr
## (Intercept) 1.451016 (Intr)
## baseToEndTx 0.106336 0.165
## Residual    1.050241
##
## Fixed effects:  bpiTotal ~ baseHamd + baseToEndTx * treatment + endOfTxToFu *      treatment
##                           Value Std.Error  DF   t-value p-value
## (Intercept)            5.233230 0.2794759 296 18.725152  0.0000
## baseHamd               0.164266 0.0267426  67  6.142482  0.0000
## baseToEndTx           -0.132705 0.0306415 296 -4.330885  0.0000
## treatment             -0.237215 0.4104391  67 -0.577954  0.5652
## endOfTxToFu           -0.028054 0.0097895 296 -2.865674  0.0045
## baseToEndTx:treatment  0.092527 0.0491582 296  1.882224  0.0608
## treatment:endOfTxToFu  0.026660 0.0152711 296  1.745793  0.0819
##  Correlation:
##                       (Intr) basHmd bsTEnT trtmnt enOTTF bsTET:
## baseHamd              -0.145
## baseToEndTx           -0.168  0.010
## treatment             -0.696  0.201  0.115
## endOfTxToFu           -0.262  0.011  0.337  0.179
## baseToEndTx:treatment  0.104 -0.004 -0.623 -0.161 -0.210
## treatment:endOfTxToFu  0.165  0.012 -0.216 -0.246 -0.641  0.326
##
## Standardized Within-Group Residuals:
##         Min          Q1         Med          Q3         Max
## -2.80833876 -0.54315722 -0.00701104  0.49644406  3.30551782
##
## Number of Observations: 370
## Number of Groups: 70

#yoga only
yogaBpiDat <- bpiDat %>% filter(treatment == 0)

model8 <- lme(bpiTotal ~ baseHamd + baseToEndTx + endOfTxToFu,
                 data = yogaBpiDat,
                 method = "REML",
                 random = ~baseToEndTx|id,
                 na.action = "na.exclude")
summary(model8)
## Linear mixed-effects model fit by REML
##   Data: yogaBpiDat
##       AIC      BIC   logLik
##   777.782 804.7098 -380.891
##
## Random effects:
##  Formula: ~baseToEndTx | id
##  Structure: General positive-definite, Log-Cholesky parametrization
##             StdDev     Corr
## (Intercept) 1.36474372 (Intr)
## baseToEndTx 0.08524191 -0.202
## Residual    1.09191209
##
```

```
## Fixed effects:  bpiTotal ~ baseHamd + baseToEndTx + endOfTxToFu
##                  Value  Std.Error  DF   t-value p-value
## (Intercept)  5.267804 0.27099047 180 19.439073  0.0000
## baseHamd     0.141179 0.03215727  34  4.390271  0.0001
## baseToEndTx -0.133724 0.02873195 180 -4.654207  0.0000
## endOfTxToFu -0.028096 0.01016776 180 -2.763276  0.0063
##  Correlation:
##             (Intr) basHmd bsTEnT
## baseHamd    -0.178
## baseToEndTx -0.391  0.029
## endOfTxToFu -0.283  0.015  0.363
##
## Standardized Within-Group Residuals:
##        Min         Q1        Med         Q3        Max
## -2.7024028 -0.5056943  0.0196024  0.4934906  3.1228176
##
## Number of Observations: 218
## Number of Groups: 36

#cbt only
cbtBpiDat <- bpiDat %>% filter(treatment == 1)

model9 <- lme(bpiTotal ~ baseHamd + baseToEndTx + endOfTxToFu,
                 data = cbtBpiDat,
                 method = "REML",
                 random = ~baseToEndTx|id,
                 na.action = "na.exclude")
summary(model9)
## Linear mixed-effects model fit by REML
##   Data: cbtBpiDat
##        AIC      BIC    logLik
##   548.7018 572.6795 -266.3509
##
## Random effects:
##  Formula: ~baseToEndTx | id
##  Structure: General positive-definite, Log-Cholesky parametrization
##             StdDev    Corr
## (Intercept) 1.5511829 (Intr)
## baseToEndTx 0.1414245 0.387
## Residual    0.9797010
##
## Fixed effects:  bpiTotal ~ baseHamd + baseToEndTx + endOfTxToFu
##                  Value  Std.Error  DF   t-value p-value
## (Intercept)  5.041166 0.30929798 116 16.298735  0.0000
## baseHamd     0.192819 0.04308388  32  4.475441  0.0001
## baseToEndTx -0.054746 0.04276548 116 -1.280153  0.2030
## endOfTxToFu -0.001393 0.01098669 116 -0.126774  0.8993
##  Correlation:
##             (Intr) basHmd bsTEnT
## baseHamd     0.218
## baseToEndTx  0.031  0.024
## endOfTxToFu -0.206  0.039  0.286
##
```

```
## Standardized Within-Group Residuals:
##        Min         Q1        Med         Q3        Max
## -2.3741087 -0.5247129 -0.0738984  0.5120231  2.8504194
##
## Number of Observations: 152
## Number of Groups: 34

#cohen's d
painTotCohenDat <- bpiDat %>%
        group_by(treatment, week) %>%
        summarise(meanPainTot = mean(bpiTotal, na.rm = T)) %>%
        ungroup() %>%
        group_by(week) %>%
        mutate(meanDif = last(meanPainTot) - first(meanPainTot)) %>%
        ungroup() %>%
        mutate(painTotCohenD = purrr::map_dbl(meanDif, nlmeCohenD, model7)) %>%
        filter(week >= 10) %>%
        select(treatment, week, meanPainTot, painTotCohenD)
## `summarise()` has grouped output by 'treatment'. You can override using the
## `.groups` argument.

painTotFig <- plot(ggpredict(model7, c("endOfTxToFu", "treatment"), ci.lvl = NA), use.theme = F,
     connect.lines = TRUE, colors = "bw") +
        ylim(3,6) +
        geom_point() +
        theme(axis.text=element_text(size = 18),
                                              axis.title=element_text(size = 18, face="bold"),
                                              strip.text.x = element_text(size = 18)) +
        scale_color_manual(name = "",
                           labels = c("Yoga", "CBT"),
                           values = c("#000000", "#000000"),
                           guide = guide_legend(reverse = F)) +
        scale_linetype_manual(name = "",
                           labels = c("Yoga", "CBT"),
                           values = c("dashed", "solid"),
                           guide = guide_legend(reverse = F)) +
        jtools::theme_apa(legend.pos = "top", x.font.size = 18, y.font.size = 18,
                          facet.title.size = 18, legend.use.title = F) +
        scale_x_continuous(breaks=c(0, 8, 16, 24),
                        labels=c("EOT", "2-mos", "4-mos", "6-mos")) +
        theme(axis.text.x = element_text(size = 18),
              axis.text.y = element_text(size = 18),
              panel.border = element_blank()) +
        labs(tag = "C", title = "", size = 18) +
        xlab("") + ylab("BPI-SF Pain Total Score") +
        theme(axis.line = element_line(color = "black")) +
        theme(legend.text=element_text(size=18))
## Scale for y is already present.
## Adding another scale for y, which will replace the existing scale.
## Scale for colour is already present.
## Adding another scale for colour, which will replace the existing scale.
```

Check Assumptions: Model 9

```
#check for independence and normality of within-group errors (participants)
plot(model7, resid(., type="p") ~ fitted(.), abline=0)
```

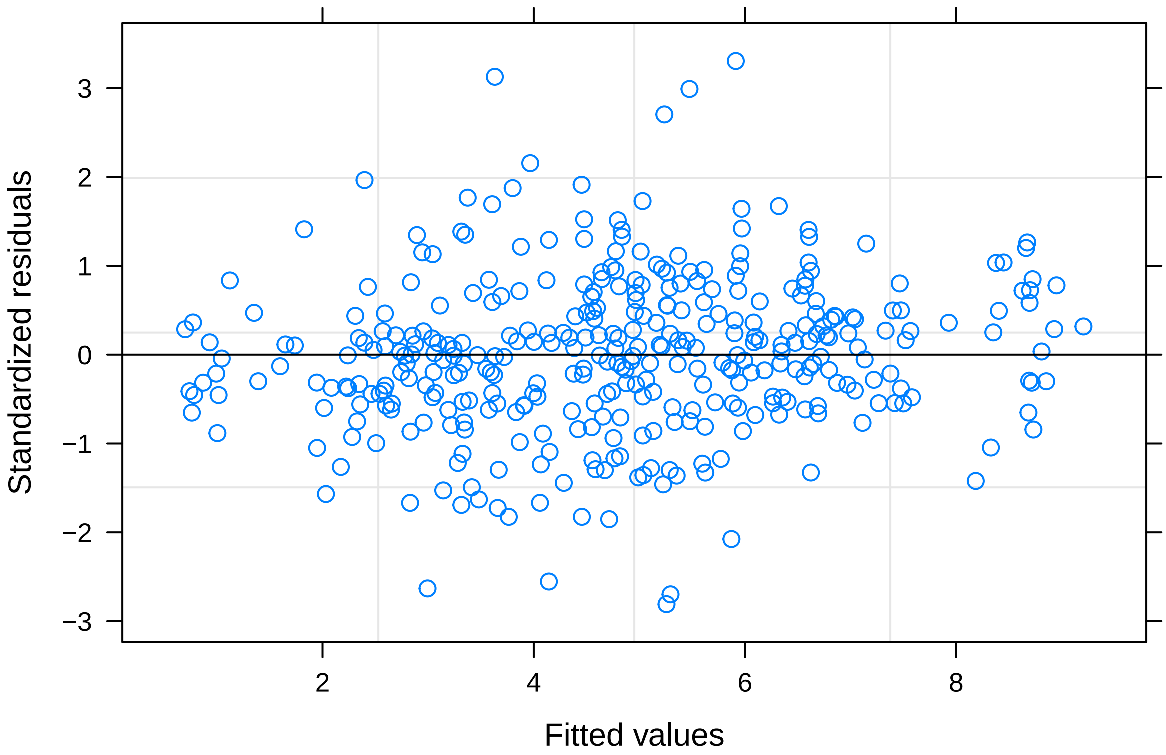


```
plot(model7, id~resid(.), abline = 0 )
```

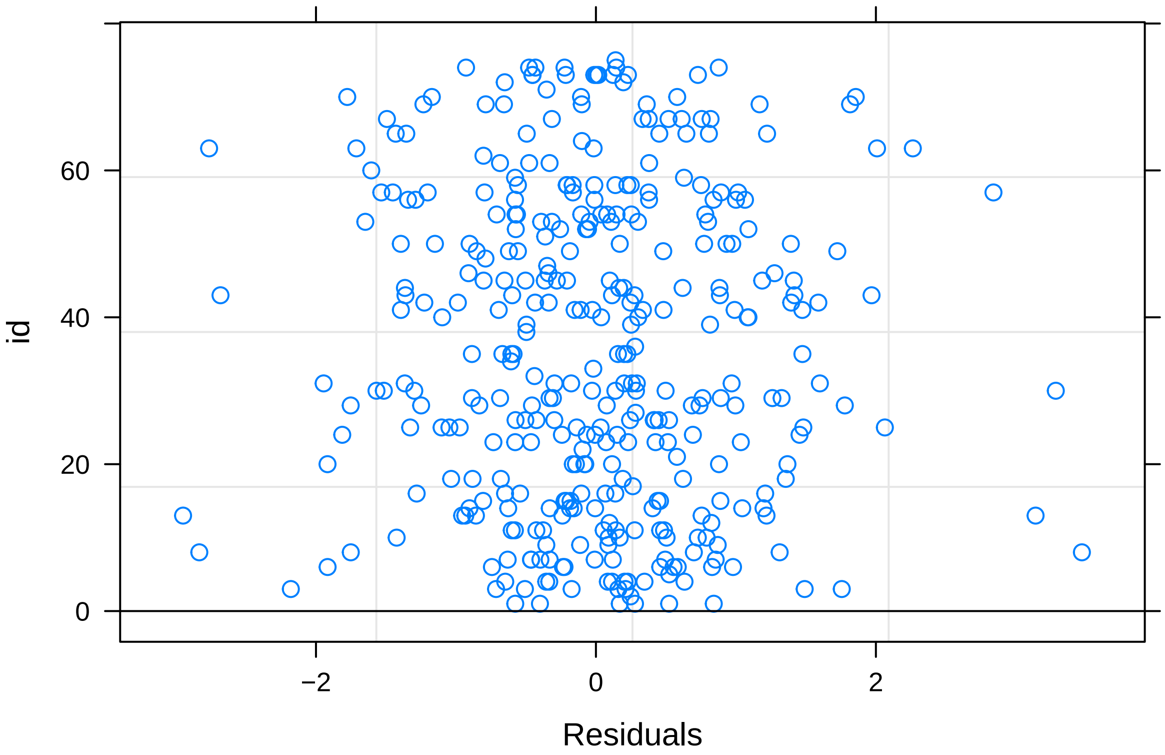


```
plot(model7, resid(., type = "p") ~ fitted(.) | week, id = 0.05, adj = -0.3 )
```

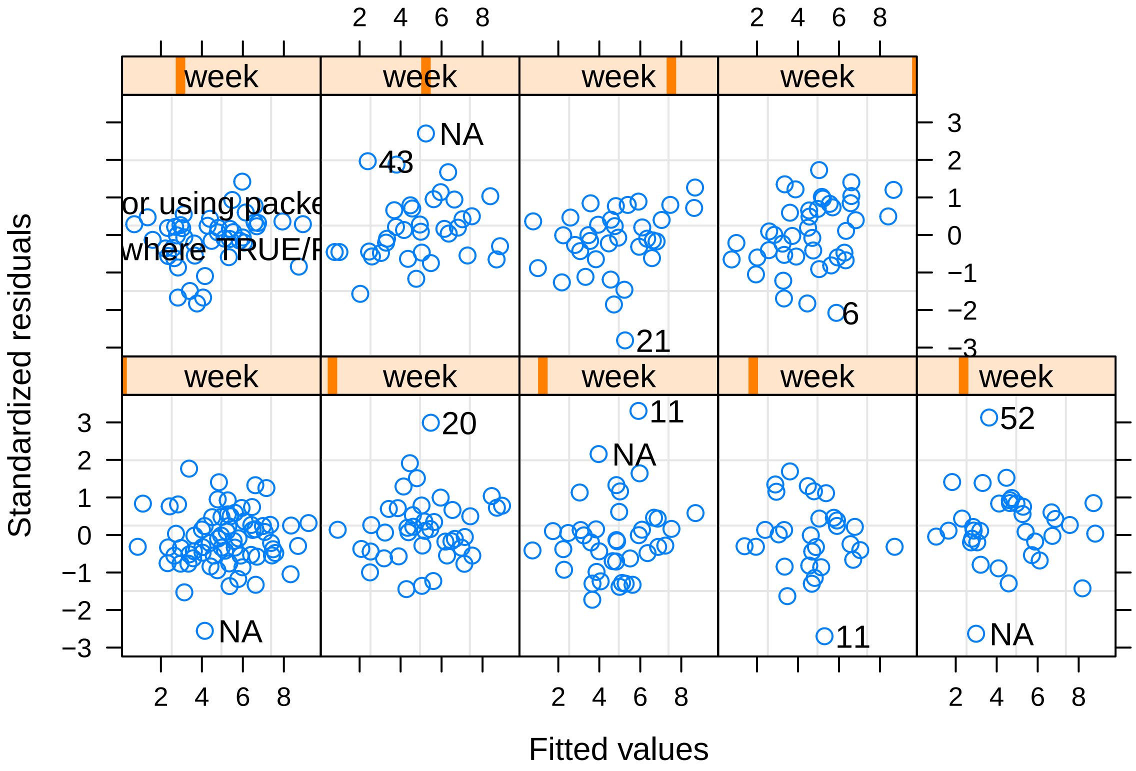

```
plot(model7, painInterferenceMean ~ fitted(.) | week, id = 0.05, adj = -0.3 )
```

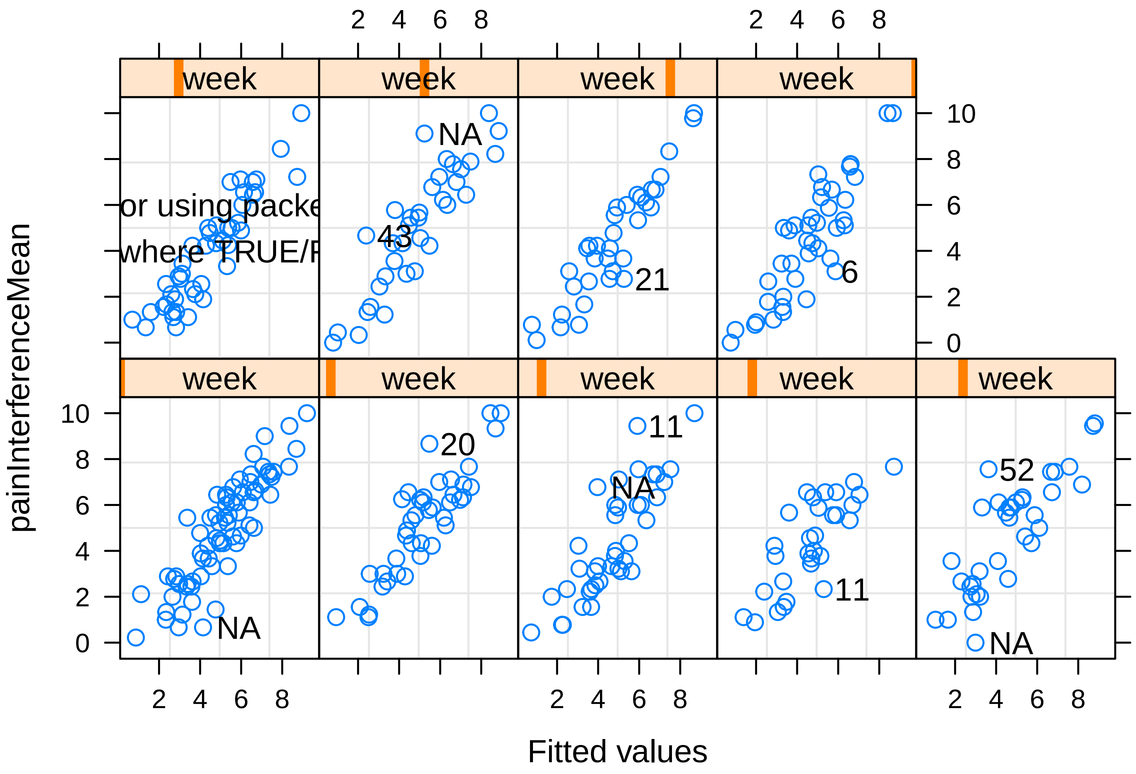


```
plot(model7, painInterferenceMean ~ fitted(.), id=.05, adj=-0.3)
```

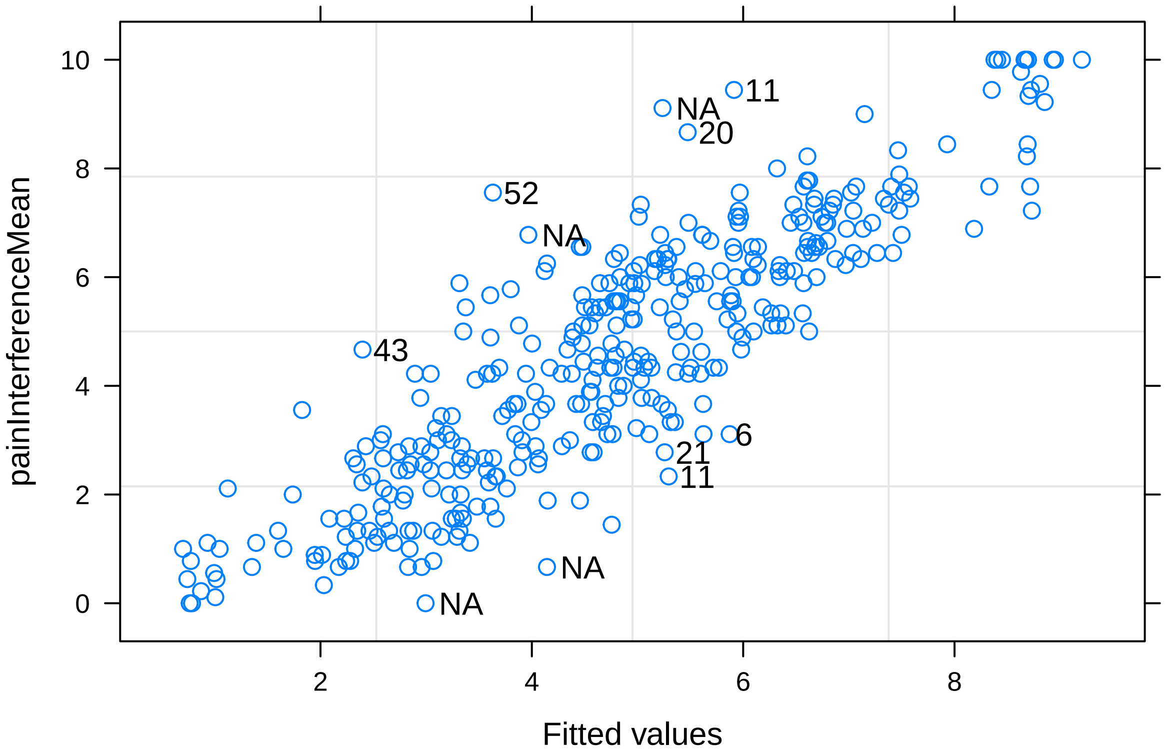

```
hist(residuals(model7), breaks=20, id=.05)
## Warning in plot.window(xlim, ylim, "", ...): "id" is not a graphical parameter
## Warning in title(main = main, sub = sub, xlab = xlab, ylab = ylab, ...): "id"
## is not a graphical parameter
## Warning in axis(1, ...): "id" is not a graphical parameter
## Warning in axis(2, at = yt, ...): "id" is not a graphical parameter
```

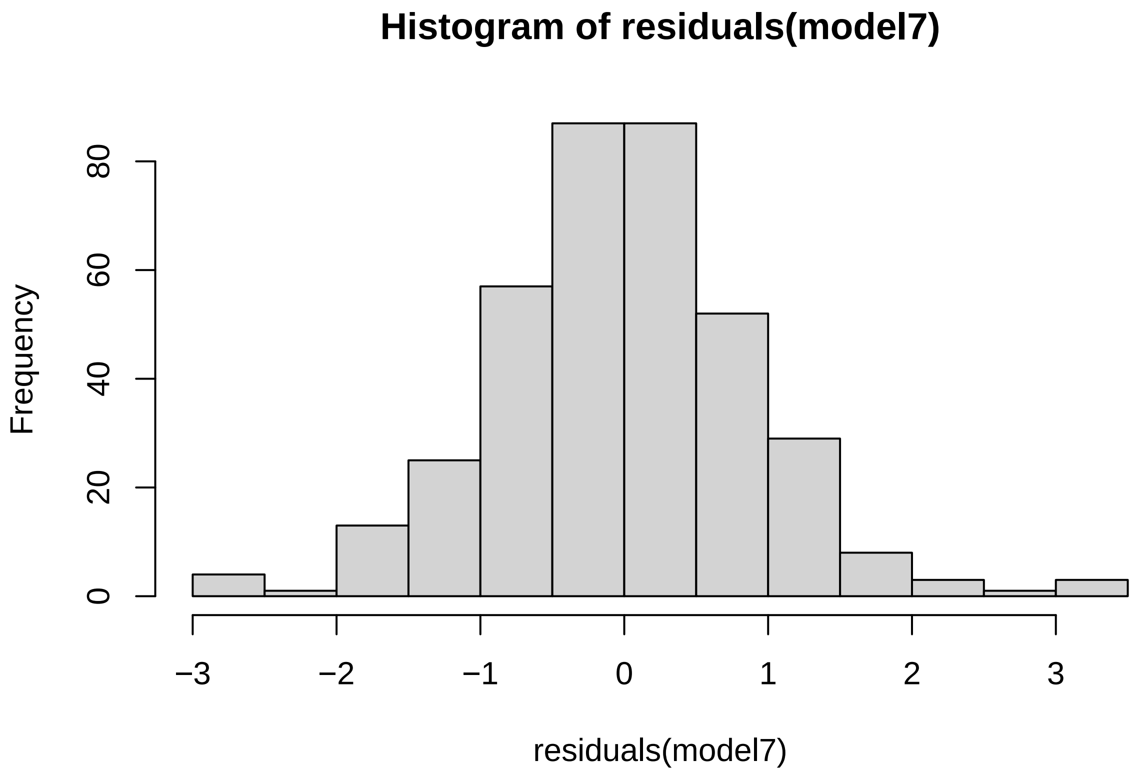


```
qqnorm(model7)
```

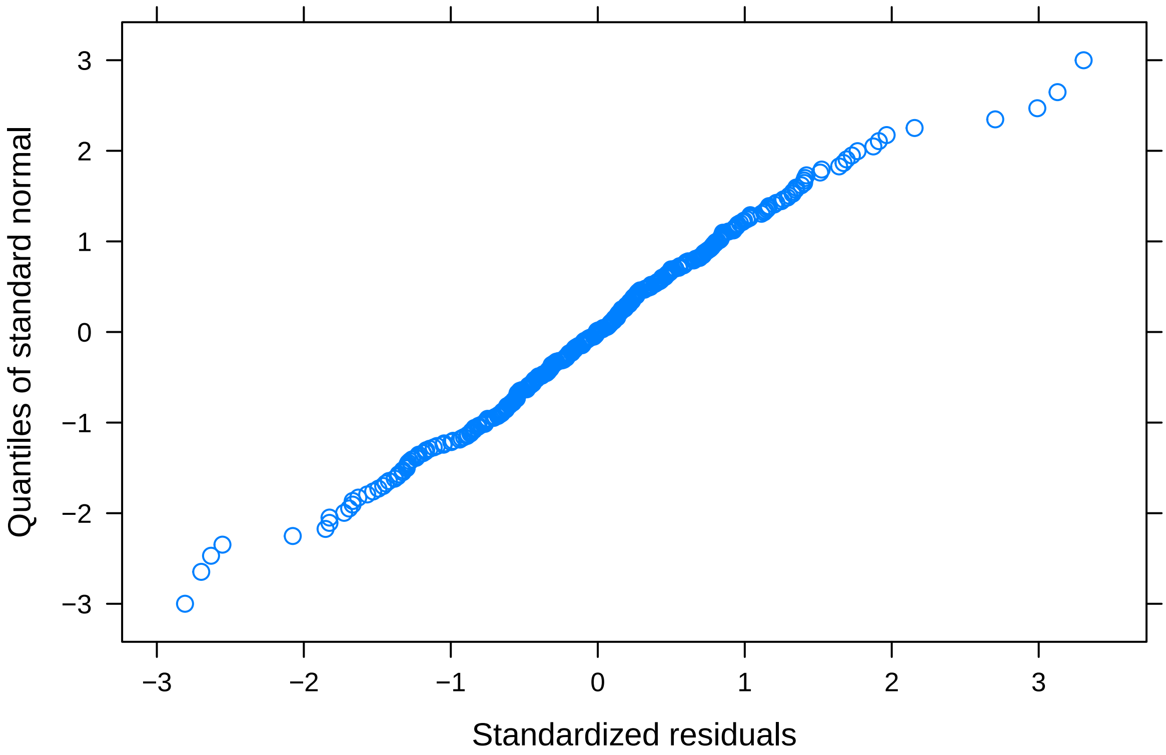


```
#check for normality and independence of random effects
qqnorm(model7, ~ranef(.), id=0.10, cex=0.7)
```

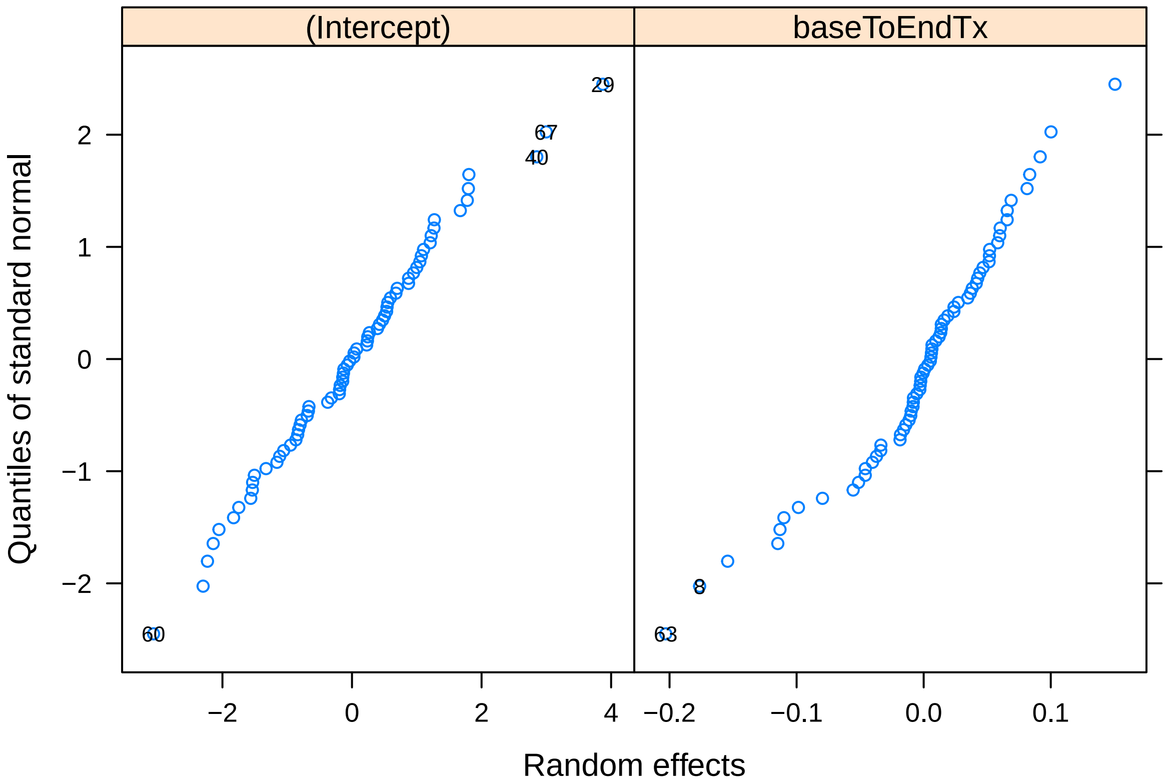

```
pairs(model7, ~ranef(., level=1), adj=-0.3)
```

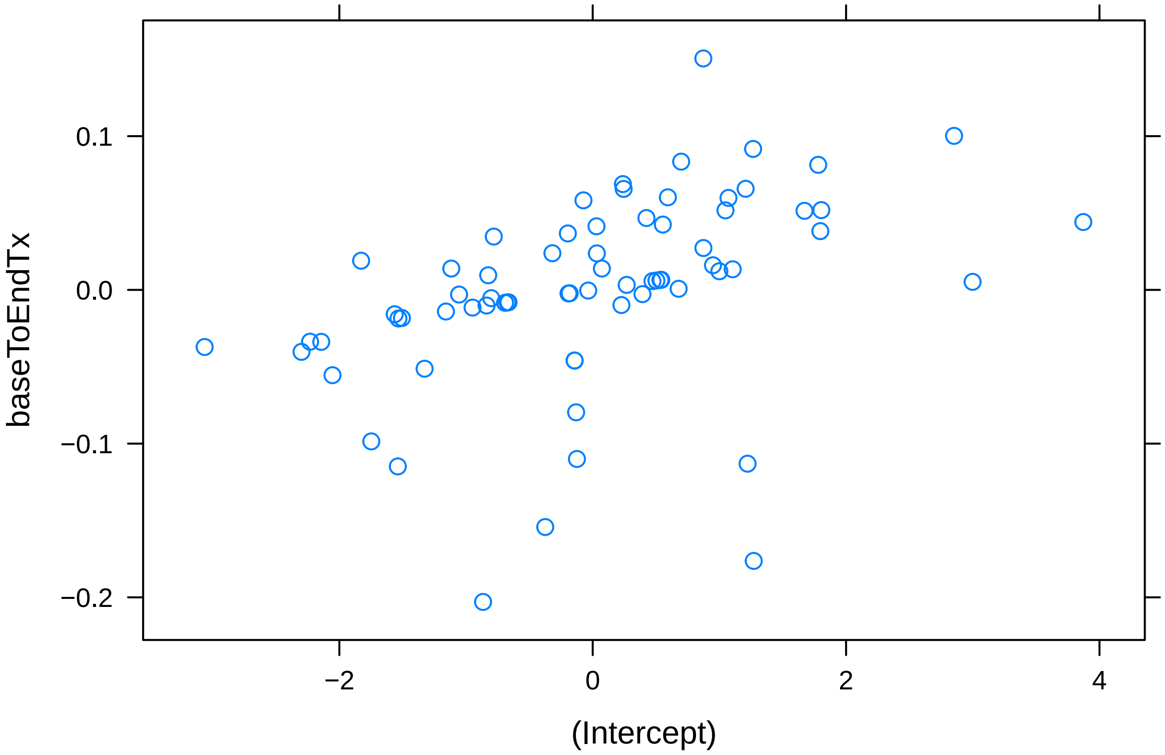

Panel of Figures

```
library(patchwork)
painSevFig + painIntFig + painTotFig
```

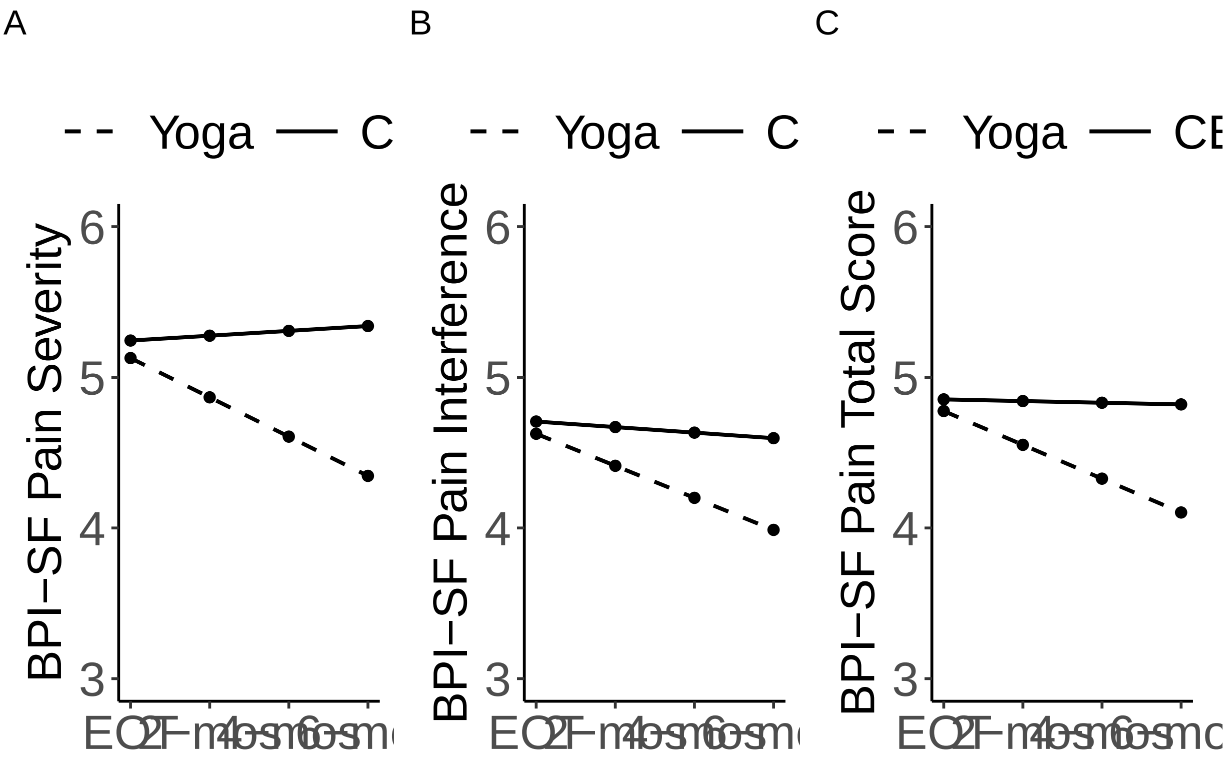


```
plot_layout(ncol = 3)
```

```
## $ncol
## [1] 3
##
## $nrow
## NULL
##
## $byrow
## NULL
##
## $widths
## NULL
##
## $heights
## NULL
##
## $guides
## NULL
##
## $tag_level
## NULL
##
## $design
## NULL
##
## attr(,"class")
## [1] "plot_layout"
```

```
# sjPlot::save_plot(
#   "figPanel.png",
#   fig = last_plot(),
#   width = 45,
#   height = 20,
#   dpi = 300,
#   theme = theme_get(),
#   label.color = "black",
#   label.size = 2.0,
#   axis.textsize = 0.5,
#   axis.titlesize = 0.75,
#   legend.textsize = 0.6,
#   legend.titlesize = 0.60,
#   legend.itemsize = 0.5
# )
```

Generate Pain Table

```
painSevModel <- lme(painSeverityMean ~  baseHamd + baseToEndTx*treatment + endOfTxToFu + I(endOfTxToFu^2
              data = bpiDat,
              method = "REML",
              na.action = "na.exclude",
              random = ~baseToEndTx | id)

painIntModel <- lme(painInterferenceMean ~ baseHamd + baseToEndTx*treatment + endOfTxToFu*treatment,
              data = bpiDat,
              method = "REML",
              na.action = "na.exclude",
              random = ~baseToEndTx | id)

painTotModel <- lme(bpiTotal ~ baseHamd + baseToEndTx*treatment + endOfTxToFu*treatment,
                 data = bpiDat,
                 method = "REML",
                 random = ~baseToEndTx|id,
                 na.action = "na.exclude")

# Generate Table
# sjPlot::tab_model(painSevModel, painIntModel, painTotModel,
#                   show.se = T,
#                   show.stat = T,
#                   show.df = F,
#                   show.p = T,
#                   file = "modelOfTables.doc")
```

Generate Cohen's D Table

```
library(rempsyc)
```

```
## Suggested APA citation: Thériault, R. (2022). rempsyc: Convenience functions for psychology
## (R package version 0.1.1) [Computer software]. https://rempsyc.remi-theriault.com
```

```r
library(flextable)
```

```
##
## Attaching package: 'flextable'

## The following object is masked from 'package:purrr':
##
##     compose
```

```r
library(officer)

primCohTable <- left_join(painSevCohenDat, painIntCohenDat, by = c("treatment", "week")) %>%
        left_join(., painTotCohenDat, by = c("treatment", "week")) %>%
        mutate(treatment = ifelse(treatment == 0, "Yoga", "CBT")) %>%
        mutate(week = case_when(
                week == 10 ~ "EOT",
                week == 18 ~ "2m FU",
                week == 26 ~ "4m FU",
                week == 34 ~ "6m FU"
        ))

primCohTable <- nice_table(primCohTable)

sect_properties <- prop_section(
  page_size = page_size(
    orient = "landscape",
    width = 8.3, height = 11.7
  ))

# save_as_docx(primCohTable, path = "primaryCohenTable.docx",
#              pr_section = sect_properties)

# standard errors and NA per time point
bpiDat %>%
        group_by(treatment, week) %>%
        summarise(painIntSd = sd(painInterferenceMean, na.rm = T),
                  painSevSd = sd(painSeverityMean, na.rm = T),
                  painTotSd = sd(bpiTotal, na.rm = T),
                  numNaInt = sum(!is.na(painInterferenceMean)),
                  numNaSev = sum(!is.na(painSeverityMean)),
                  numNaTot = sum(!is.na(bpiTotal))) %>%
        filter(week >= 10) %>%
        ungroup()
```

```
## `summarise()` has grouped output by 'treatment'. You can override using the
## `.groups` argument.

## # A tibble: 8 x 8
##   treatment  week painIntSd painSevSd painTotSd numNaInt numNaSev numNaTot
```

```
##       <dbl> <dbl>     <dbl>     <dbl>     <dbl>    <int>    <int>    <int>
## 1         0    10      2.17      2.44      2.08       31       32       31
## 2         0    18      2.47      1.89      2.19       23       24       23
## 3         0    26      2.44      2.34      2.24       23       23       23
## 4         0    34      2.49      1.93      2.17       27       29       27
## 5         1    10      2.60      2.60      2.35       16       19       16
## 6         1    18      3.15      2.43      2.87       15       15       15
## 7         1    26      2.78      2.55      2.68       14       15       14
## 8         1    34      2.71      2.67      2.64       17       18       17
```

Clinically Significant Improvements: pain

```
#pain severity- cross-checked with week == 10 in original paper
bpiDat %>%
        filter(week == 0 | week == 34) %>%
        group_by(id) %>%
        mutate(sevPercentChange =
                       ((last(painSeverityMean) - first(painSeverityMean)) /
                                first(painSeverityMean)) * 100 ) %>%
        ungroup() %>%
        #filter(week == 34) %>%
        filter(week == 34 & !is.na(painSeverityMean)) %>%
        group_by(treatment) %>%
        summarise(greater15Reduction = (sum(sevPercentChange <= -15.00, na.rm = T)) / n(),
                  sumSevChange = sum(sevPercentChange <= -15.00, na.rm = T),
                  n() )
```

```
## # A tibble: 2 x 4
##   treatment greater15Reduction sumSevChange `n()`
##       <dbl>              <dbl>        <int> <int>
## 1         0              0.448           13    29
## 2         1              0.278            5    18
```

```
#chi square test: pain severity
#https://statsandr.com/blog/fisher-s-exact-test-in-r-independence-test-for-a-small-sample/
chiSquareSevDat <- bpiDat %>%
        filter(week == 0 | week == 34) %>%
        group_by(id) %>%
        mutate(sevPercentChange =
                       ((last(painSeverityMean) - first(painSeverityMean)) /
                                first(painSeverityMean)) * 100 ) %>%
        ungroup() %>%
        #filter(week == 34) %>%
        filter(week == 34 & !is.na(painSeverityMean)) %>%
        group_by(treatment) %>%
        mutate(greater15Reduction = ifelse(sevPercentChange <= -15.00, "signRed", "nonSignRed")) %>%
        ungroup()

fisher.test(table(chiSquareSevDat$treatment, chiSquareSevDat$greater15Reduction))
```

```
##
```

```
##  Fisher's Exact Test for Count Data
##
## data:  table(chiSquareSevDat$treatment, chiSquareSevDat$greater15Reduction)
## p-value = 0.3563
## alternative hypothesis: true odds ratio is not equal to 1
## 95 percent confidence interval:
##  0.1052606 1.9382133
## sample estimates:
## odds ratio
##  0.4809063
```

```
#pain interference- cross-checked with week == 10 in original paper
bpiDat %>%
        filter(week == 0 | week == 34) %>%
        group_by(id) %>%
        mutate(baseFuDif = last(painInterferenceMean) - first(painInterferenceMean)) %>%
        #filter(week == 34) %>%
        filter(week == 34 & !is.na(painInterferenceMean)) %>%
        group_by(treatment) %>%
        summarise(greater1PtReduction = (sum(baseFuDif <= -1.00, na.rm = T)) / n(),
                  sumBaseFuDif = sum(baseFuDif <= -1.00, na.rm = T),
                  n() )
```

```
## # A tibble: 2 x 4
##   treatment greater1PtReduction sumBaseFuDif `n()`
##       <dbl>               <dbl>        <int> <int>
## 1         0               0.481           13    27
## 2         1               0.235            4    17
```

```
#chi square test: pain interference
chiSquareIntDat <- bpiDat %>%
        filter(week == 0 | week == 34) %>%
        group_by(id) %>%
        mutate(baseFuDif = last(painInterferenceMean) - first(painInterferenceMean)) %>%
        ungroup() %>%
        #filter(week == 34) %>%
        filter(week == 34 & !is.na(painInterferenceMean)) %>%
        group_by(treatment) %>%
        mutate(greater1PtReduction = ifelse(baseFuDif <= -1.00, "signRed", "nonSignRed")) %>%
        ungroup()

fisher.test(table(chiSquareIntDat$treatment, chiSquareIntDat$greater1PtReduction))
```

```
##
##  Fisher's Exact Test for Count Data
##
## data:  table(chiSquareIntDat$treatment, chiSquareIntDat$greater1PtReduction)
## p-value = 0.1245
## alternative hypothesis: true odds ratio is not equal to 1
## 95 percent confidence interval:
##  0.06381233 1.48681642
## sample estimates:
## odds ratio
##  0.3397751
```

Sensitivity Analysis

```
sensAnalDat <- bpiDat %>%
        select(id, treatment, week, baseToEndTx, endOfTxToFu, baseHamd,
               painInterferenceMean, painSeverityMean, bpiTotal)

ggplot(sensAnalDat,
       aes(x = bpiTotal, y = week)) +
        geom_miss_point() +
        facet_wrap(~id)
```

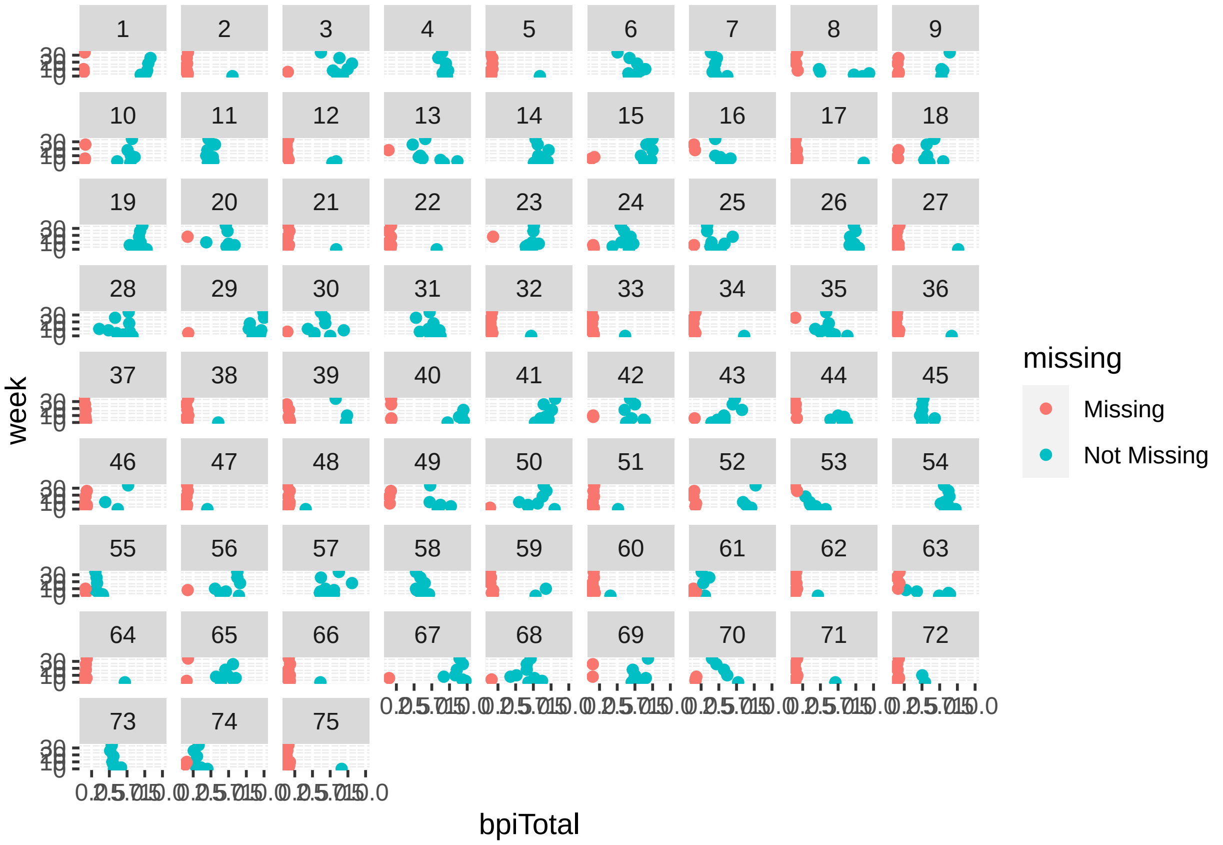


```
sensAnalDat <- sensAnalDat %>%
        group_by(id, week) %>%
        mutate(sevMiss = ifelse(is.na(painSeverityMean), 1, 0)) %>%
        mutate(intMiss = ifelse(is.na(painInterferenceMean), 1, 0)) %>%
        mutate(totMiss = ifelse(is.na(bpiTotal), 1, 0)) %>%
        ungroup() %>%
        group_by(id) %>%
        mutate(sevMissPattern = ifelse(any(week >= 18 & sevMiss == 1), 1, 0)) %>%
        mutate(intMissPattern = ifelse(any(week >= 18 & intMiss == 1), 1, 0)) %>%
        mutate(totMissPattern = ifelse(any(week >= 18 & totMiss == 1), 1, 0))

painSevModel <- lme(painSeverityMean ~  baseHamd + baseToEndTx*treatment + endOfTxToFu + I(endOfTxToFu^
              data = sensAnalDat,
              method = "REML",
```

```r
             na.action = "na.exclude",
             random = ~baseToEndTx | id)

summary(painSevModel)
```

```
## Linear mixed-effects model fit by REML
##   Data: sensAnalDat
##        AIC      BIC    logLik
##   1372.335 1434.994 -670.1676
##
## Random effects:
##  Formula: ~baseToEndTx | id
##  Structure: General positive-definite, Log-Cholesky parametrization
##             StdDev    Corr
## (Intercept) 1.5374812 (Intr)
## baseToEndTx 0.1060579 0.31
## Residual    0.9795392
##
## Fixed effects:  painSeverityMean ~ baseHamd + baseToEndTx * treatment + endOfTxToFu +      I(endOfTx
##                                                Value Std.Error  DF   t-value
## (Intercept)                                 5.501086 0.3953589 305 13.914159
## baseHamd                                    0.117500 0.0282749  66  4.155621
## baseToEndTx                                -0.086582 0.0293983 305 -2.945136
## treatment                                   0.001434 0.5941454  66  0.002414
## endOfTxToFu                                -0.018696 0.0270413 305 -0.691373
## I(endOfTxToFu^2)                           -0.000392 0.0012134 305 -0.323209
## sevMissPattern                             -0.171196 0.5781935  66 -0.296088
## baseToEndTx:treatment                       0.050539 0.0454110 305  1.112914
## treatment:I(endOfTxToFu^2)                  0.001057 0.0006887 305  1.534418
## I(endOfTxToFu^2):sevMissPattern            -0.000171 0.0008101 305 -0.210650
## treatment:sevMissPattern                   -0.030736 0.8209647  66 -0.037438
## treatment:I(endOfTxToFu^2):sevMissPattern   0.001953 0.0013584 305  1.437975
##                                            p-value
## (Intercept)                                 0.0000
## baseHamd                                    0.0001
## baseToEndTx                                 0.0035
## treatment                                   0.9981
## endOfTxToFu                                 0.4899
## I(endOfTxToFu^2)                            0.7468
## sevMissPattern                              0.7681
## baseToEndTx:treatment                       0.2666
## treatment:I(endOfTxToFu^2)                  0.1260
## I(endOfTxToFu^2):sevMissPattern             0.8333
## treatment:sevMissPattern                    0.9702
## treatment:I(endOfTxToFu^2):sevMissPattern   0.1515
##  Correlation:
##                                            (Intr) basHmd bsTEnT trtmnt enOTTF
## baseHamd                                    0.022
## baseToEndTx                                -0.035  0.006
## treatment                                  -0.645  0.102  0.002
## endOfTxToFu                                -0.159  0.005  0.198 -0.003
## I(endOfTxToFu^2)                            0.063 -0.004 -0.094  0.059 -0.928
## sevMissPattern                             -0.684 -0.187 -0.004  0.424  0.084
```

```
## baseToEndTx:treatment                       0.003  0.003 -0.622 -0.010 -0.002
## treatment:I(endOfTxToFu^2)                  0.147  0.001 -0.157 -0.230  0.007
## I(endOfTxToFu^2):sevMissPattern             0.124 -0.009  0.001 -0.084  0.009
## treatment:sevMissPattern                    0.469  0.085  0.017 -0.707  0.013
## treatment:I(endOfTxToFu^2):sevMissPattern -0.073  0.020 -0.001  0.117 -0.010
##                                           I(nOTTF^2) svMssP bsTET: tr:I(OTTF^2)
## baseHamd
## baseToEndTx
## treatment
## endOfTxToFu
## I(endOfTxToFu^2)
## sevMissPattern                            -0.023
## baseToEndTx:treatment                     -0.057      0.012
## treatment:I(endOfTxToFu^2)                -0.252     -0.097  0.242
## I(endOfTxToFu^2):sevMissPattern           -0.205     -0.186  0.001  0.346
## treatment:sevMissPattern                  -0.051     -0.689 -0.012  0.161
## treatment:I(endOfTxToFu^2):sevMissPattern  0.126      0.108  0.013 -0.474
##                                           I(OTTF^2): trt:MP
## baseHamd
## baseToEndTx
## treatment
## endOfTxToFu
## I(endOfTxToFu^2)
## sevMissPattern
## baseToEndTx:treatment
## treatment:I(endOfTxToFu^2)
## I(endOfTxToFu^2):sevMissPattern
## treatment:sevMissPattern                    0.132
## treatment:I(endOfTxToFu^2):sevMissPattern -0.597     -0.169
##
## Standardized Within-Group Residuals:
##         Min          Q1         Med          Q3         Max
## -3.21881622 -0.45193033 -0.02439874  0.51311667  3.99677604
##
## Number of Observations: 383
## Number of Groups: 71
```

```
painIntModel <- lme(painInterferenceMean ~ baseHamd + baseToEndTx*treatment + endOfTxToFu*treatment*intl
              data = sensAnalDat,
              method = "REML",
              na.action = "na.exclude",
              random = ~baseToEndTx | id)

summary(painIntModel)
```

```
## Linear mixed-effects model fit by REML
##   Data: sensAnalDat
##        AIC      BIC    logLik
##   1432.925 1491.175 -701.4627
##
## Random effects:
##  Formula: ~baseToEndTx | id
##  Structure: General positive-definite, Log-Cholesky parametrization
##             StdDev    Corr
```

```
## (Intercept) 1.6069348 (Intr)
## baseToEndTx 0.1224566 0.066
## Residual    1.2177143
##
## Fixed effects:  painInterferenceMean ~ baseHamd + baseToEndTx * treatment + endOfTxToFu *      treatr
##                                           Value Std.Error  DF   t-value p-value
## (Intercept)                            5.189397 0.4471344 294 11.605898  0.0000
## baseHamd                               0.184723 0.0302705  65  6.102404  0.0000
## baseToEndTx                           -0.152415 0.0353652 294 -4.309748  0.0000
## treatment                             -0.153644 0.6876753  65 -0.223426  0.8239
## endOfTxToFu                           -0.020531 0.0136393 294 -1.505248  0.1333
## intMissPattern                        -0.081400 0.6139152  65 -0.132591  0.8949
## baseToEndTx:treatment                  0.106445 0.0567059 294  1.877148  0.0615
## treatment:endOfTxToFu                  0.003164 0.0210496 294  0.150315  0.8806
## endOfTxToFu:intMissPattern            -0.019386 0.0229156 294 -0.845977  0.3983
## treatment:intMissPattern              -0.190730 0.8945769  65 -0.213207  0.8318
## treatment:endOfTxToFu:intMissPattern  0.062504 0.0366479 294  1.705537  0.0892
##  Correlation:
##                                       (Intr) basHmd bsTEnT trtmnt enOTTF intMsP
## baseHamd                               0.030
## baseToEndTx                           -0.180  0.009
## treatment                             -0.647  0.085  0.118
## endOfTxToFu                           -0.289  0.004  0.288  0.189
## intMissPattern                        -0.714 -0.180  0.027  0.446  0.180
## baseToEndTx:treatment                  0.112 -0.009 -0.624 -0.183 -0.180 -0.016
## treatment:endOfTxToFu                  0.188  0.001 -0.187 -0.296 -0.648 -0.118
## endOfTxToFu:intMissPattern             0.143 -0.016 -0.013 -0.095 -0.550 -0.194
## treatment:intMissPattern               0.489  0.080 -0.019 -0.740 -0.124 -0.678
## treatment:endOfTxToFu:intMissPattern -0.089  0.025  0.009  0.143  0.344  0.119
##                                       bsTET: tr:OTTF eOTTF: trt:MP
## baseHamd
## baseToEndTx
## treatment
## endOfTxToFu
## intMissPattern
## baseToEndTx:treatment
## treatment:endOfTxToFu                  0.276
## endOfTxToFu:intMissPattern             0.008  0.356
## treatment:intMissPattern               0.048  0.202   0.134
## treatment:endOfTxToFu:intMissPattern  0.000 -0.530  -0.626 -0.191
##
## Standardized Within-Group Residuals:
##           Min            Q1           Med            Q3           Max
## -2.6715808721 -0.5014361165 -0.0006092798  0.4947657599  3.3240331545
##
## Number of Observations: 370
## Number of Groups: 70
```

```r
painTotModel <- lme(bpiTotal ~ baseHamd + baseToEndTx*treatment + endOfTxToFu*treatment*totMissPattern,
                 data = sensAnalDat,
                 method = "REML",
                 random = ~baseToEndTx|id,
                 na.action = "na.exclude")
```

```r
summary(painTotModel)
```

```
## Linear mixed-effects model fit by REML
##   Data: sensAnalDat
##        AIC      BIC    logLik
##   1331.667 1389.917 -650.8337
##
## Random effects:
##  Formula: ~baseToEndTx | id
##  Structure: General positive-definite, Log-Cholesky parametrization
##             StdDev    Corr
## (Intercept) 1.4627401 (Intr)
## baseToEndTx 0.1122364 0.141
## Residual    1.0428043
##
## Fixed effects:  bpiTotal ~ baseHamd + baseToEndTx * treatment + endOfTxToFu *      treatment * totMis
##                                         Value Std.Error  DF   t-value p-value
## (Intercept)                          5.336970 0.4045125 294 13.193585  0.0000
## baseHamd                             0.168556 0.0274128  65  6.148794  0.0000
## baseToEndTx                         -0.132616 0.0312373 294 -4.245438  0.0000
## treatment                           -0.223944 0.6219097  65 -0.360091  0.7199
## endOfTxToFu                         -0.021954 0.0117029 294 -1.875932  0.0617
## totMissPattern                      -0.205082 0.5581549  65 -0.367429  0.7145
## baseToEndTx:treatment                0.093534 0.0501270 294  1.865950  0.0630
## treatment:endOfTxToFu                0.007132 0.0180629 294  0.394831  0.6933
## endOfTxToFu:totMissPattern          -0.020048 0.0196878 294 -1.018302  0.3094
## treatment:totMissPattern             0.015754 0.8118722  65  0.019404  0.9846
## treatment:endOfTxToFu:totMissPattern  0.066652 0.0314816 294  2.117172  0.0351
##  Correlation:
##                                     (Intr) basHmd bsTEnT trtmnt enOTTF ttMssP
## baseHamd                             0.030
## baseToEndTx                         -0.128  0.008
## treatment                           -0.647  0.084  0.084
## endOfTxToFu                         -0.284  0.004  0.280  0.185
## totMissPattern                      -0.720 -0.181  0.015  0.449  0.184
## baseToEndTx:treatment                0.080 -0.004 -0.623 -0.133 -0.174 -0.010
## treatment:endOfTxToFu                0.184  0.000 -0.181 -0.289 -0.648 -0.120
## endOfTxToFu:totMissPattern           0.148 -0.016 -0.011 -0.098 -0.551 -0.200
## treatment:totMissPattern             0.494  0.081 -0.011 -0.745 -0.127 -0.680
## treatment:endOfTxToFu:totMissPattern -0.092  0.027  0.007  0.147  0.345  0.122
##                                     bsTET: tr:OTTF eOTTF: trt:MP
## baseHamd
## baseToEndTx
## treatment
## endOfTxToFu
## totMissPattern
## baseToEndTx:treatment
## treatment:endOfTxToFu                0.268
## endOfTxToFu:totMissPattern           0.007  0.357
## treatment:totMissPattern             0.033  0.203   0.138
## treatment:endOfTxToFu:totMissPattern  0.004 -0.532  -0.626 -0.195
##
## Standardized Within-Group Residuals:
```

```
##           Min            Q1           Med            Q3           Max
## -2.7006265575 -0.4880289748  0.0005214424  0.4632109814  3.3269142868
##
## Number of Observations: 370
## Number of Groups: 70
```

```
#https://rpsychologist.com/lmm-slope-missingness
emmeans::emmeans(painTotModel,
        pairwise ~ treatment | endOfTxToFu,
        at = list(endOfTxToFu = 24),
        CIs = FALSE,
        lmer.df = "asymptotic", # wald
        weights = "proportional",
        data = sensAnalDat)
```

```
## NOTE: Results may be misleading due to involvement in interactions
```

```
## $emmeans
## endOfTxToFu = 24:
##  treatment emmean    SE df lower.CL upper.CL
##          0   3.91 0.373 65     3.16     4.65
##          1   5.18 0.441 65     4.30     6.06
##
## Results are averaged over the levels of: totMissPattern
## Degrees-of-freedom method: containment
## Confidence level used: 0.95
##
## $contrasts
## endOfTxToFu = 24:
##  contrast                estimate    SE df t.ratio p.value
##  treatment0 - treatment1    -1.27 0.582 65  -2.179  0.0330
##
## Results are averaged over the levels of: totMissPattern
## Degrees-of-freedom method: containment
```

```
# proportion of missing data by group
sensAnalDat %>%
        select(id, treatment, totMissPattern) %>%
        distinct() %>%
        group_by(treatment, totMissPattern) %>%
        summarise(n = n(), prop = n / sum(n)) %>%
        ungroup() %>%
        group_by(treatment) %>%
        summarise(totMissPattern, prop = n / sum(n))
```

```
## `summarise()` has grouped output by 'treatment'. You can override using the
## `.groups` argument.
```

```
## Warning: Returning more (or less) than 1 row per `summarise()` group was deprecated in
## dplyr 1.1.0.
## i Please use `reframe()` instead.
## i When switching from `summarise()` to `reframe()`, remember that `reframe()`
##   always returns an ungrouped data frame and adjust accordingly.
```

```
## `summarise()` has grouped output by 'treatment'. You can override using the
## `.groups` argument.

## # A tibble: 4 x 3
## # Groups:   treatment [2]
##   treatment totMissPattern  prop
##       <dbl>          <dbl> <dbl>
## 1         0              0 0.487
## 2         0              1 0.513
## 3         1              0 0.333
## 4         1              1 0.667
```

```
plot(ggpredict(painTotModel, c("endOfTxToFu", "treatment", "totMissPattern"), ci.lvl = NA), use.theme =
                                        scale_x_continuous(breaks = c(0, 8, 16, 24)) +
                                        theme(axis.text=element_text(size = 14),
                                              axis.title=element_text(size = 16,face="bold"),
                                              strip.text.x = element_text(size = 16)) +
        ylim(0,7) +
        geom_point() +
        scale_colour_brewer(palette = "Set1", labels = c("yoga", "cbt"))
```

```
## Scale for y is already present.
## Adding another scale for y, which will replace the existing scale.

## Scale for colour is already present.
## Adding another scale for colour, which will replace the existing scale.
```

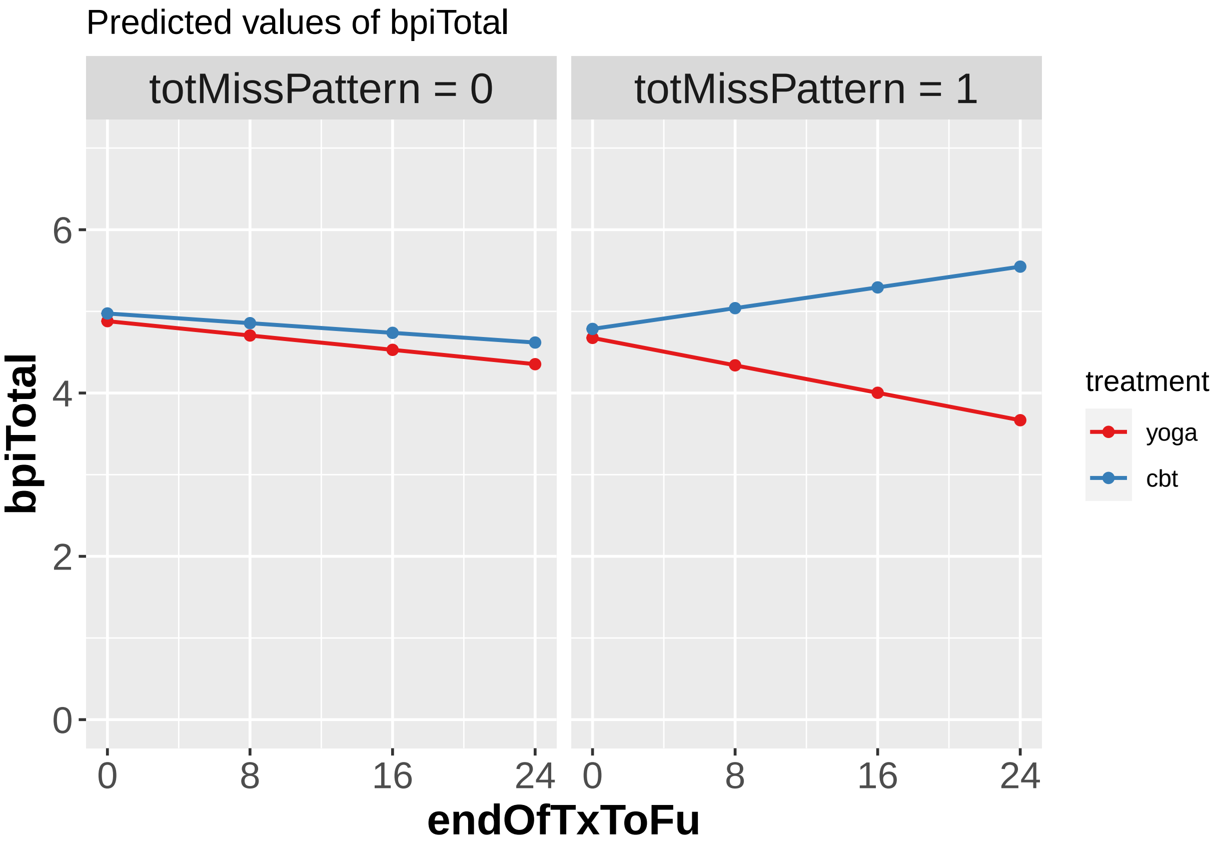

Supplement: Supplementary file 2 — Additional file 2. [file 12906_2023_4145_MOESM2_ESM.zip › primaryFuAnalyses.pdf]
