# Supplementary material for: Yoga is effective for treating chronic pain in veterans with Gulf War Illness at long-term follow-up
Source: BMC Complement Med Ther. 2023 Sep 13;23:319. doi: 10.1186/s12906-023-04145-y (PMC10498617; doi:10.1186/s12906-023-04145-y)
Supplement: Supplementary file 2 — Additional file 2. [file 12906_2023_4145_MOESM2_ESM.zip › secondaryLmms.pdf]

Load Libraries

```
library(tidyverse)
```

```
## -- Attaching packages --------------------------------------- tidyverse 1.3.2 --
## v ggplot2 3.4.1     v purrr   1.0.1
## v tibble  3.1.8     v dplyr   1.1.0
## v tidyr   1.3.0     v stringr 1.5.0
## v readr   2.1.4     v forcats 1.0.0
## -- Conflicts ------------------------------------------ tidyverse_conflicts() --
## x dplyr::filter() masks stats::filter()
## x dplyr::lag()    masks stats::lag()
```

```
library(nlme)
```

```
##
## Attaching package: 'nlme'
##
## The following object is masked from 'package:dplyr':
##
##     collapse
```

```
library(sjPlot)
library("PsychLab")
```

```
## Loading required package: lubridate
##
## Attaching package: 'lubridate'
##
## The following objects are masked from 'package:base':
##
##     date, intersect, setdiff, union
```

```
library(lmerTest)
```

```
## Loading required package: lme4
## Loading required package: Matrix
##
## Attaching package: 'Matrix'
##
## The following objects are masked from 'package:tidyr':
##
##     expand, pack, unpack
##
##
## Attaching package: 'lme4'
##
## The following object is masked from 'package:nlme':
##
##     lmList
```

```
##
##
## Attaching package: 'lmerTest'
##
## The following object is masked from 'package:lme4':
##
##     lmer
##
## The following object is masked from 'package:stats':
##
##     step
```

```
library(ggeffects)
```

Load Data

```
analyzeMe <- read_csv("data.csv")
```

```
## Rows: 75 Columns: 1449
## -- Column specification --------------------------------------------------------
## Delimiter: ","
## chr    (74): ID, Sex, Race, Ethnicity, Marital_Status, employment, service_b...
## dbl  (1367): treatment, tx_grp_mod, completed, cardiac_data, number_sessions...
## lgl     (6): pre_SDANNms, pre_SDNNIms, post_SDANNms, post_SDNNIms, followup_...
## time    (2): walk_time.rb, walk_time.wk10
##
## i Use `spec()` to retrieve the full column specification for this data.
## i Specify the column types or set `show_col_types = FALSE` to quiet this message.
```

```
options(max.print=1000)
```

Secondary Vars: Fatigue/Walk

```
walkDat <- analyzeMe %>%
        select(ID, treatment, bpiTotalBase = BPImrb, walkdrb, walkdw10, walkdw34) %>%
        rowid_to_column(., "id") %>% select(-ID) %>%
        pivot_longer(4:6, names_to = "week", values_to = "walkDistance") %>%
        mutate(week = case_when(
                week == "walkdrb" ~ 0,
                week == "walkdw10" ~ 10,
                week == "walkdw34" ~ 34
        )) %>%
        mutate(baseToEndOfTx = ifelse(week <= 10, week, 1)) %>%
        mutate(endOfTxToFu = case_when(
                week == 0 ~ 0,
                week == 10 ~ 0,
                week == 34 ~ 24
```

```
        ))

walkDat %>%
        group_by(treatment, week) %>%
        summarise(walkDistMean = mean(walkDistance, na.rm = T)) %>%
        ggplot(., aes(week, walkDistMean, color = as.factor(treatment))) +
        geom_point() +
        geom_line() +
        ylim(400,500)
```

```
## `summarise()` has grouped output by 'treatment'. You can override using the
## `.groups` argument.
```

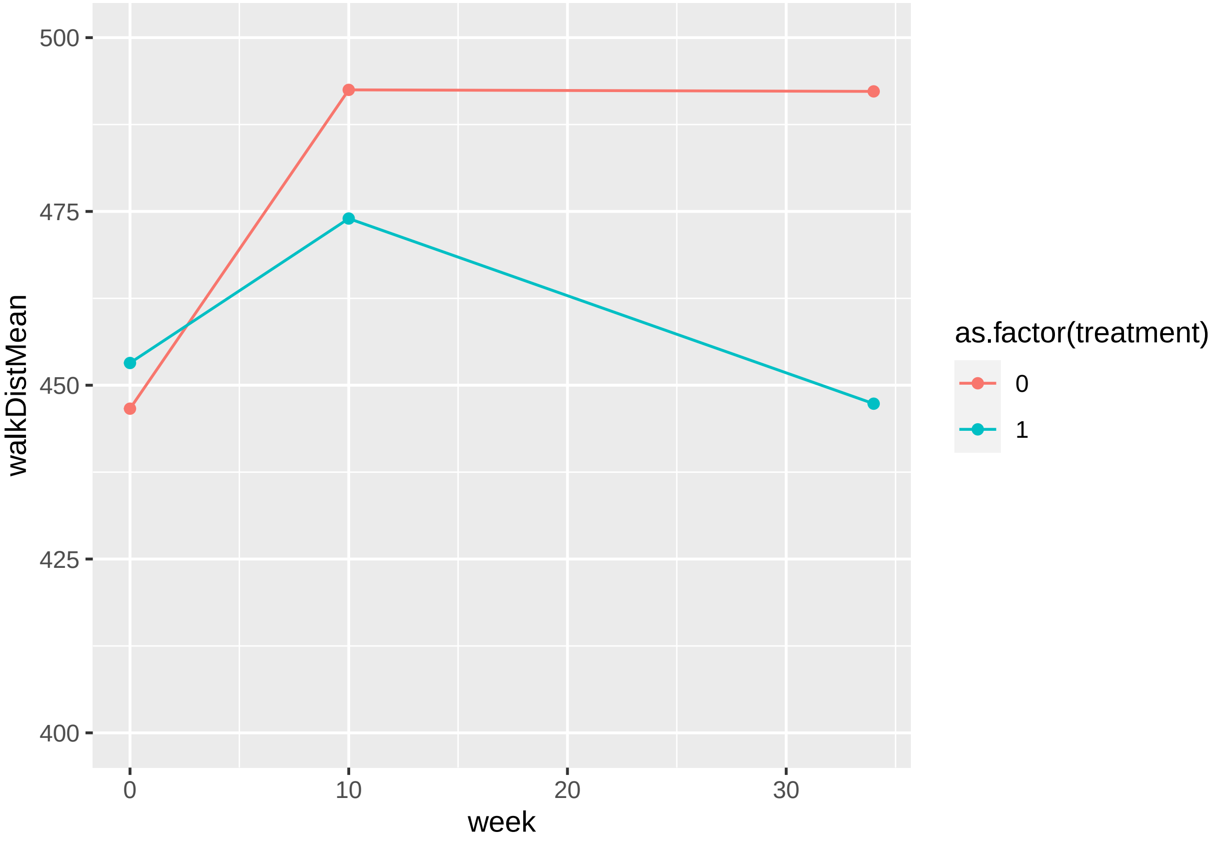


```
cor.test(analyzeMe$BPImrb, analyzeMe$walkdrb)
```

```
##
##  Pearson's product-moment correlation
##
## data:  analyzeMe$BPImrb and analyzeMe$walkdrb
## t = -3.0642, df = 66, p-value = 0.003159
## alternative hypothesis: true correlation is not equal to 0
## 95 percent confidence interval:
##  -0.5454382 -0.1250000
## sample estimates:
##        cor
## -0.3529074
```

```r
###build models: step-up procedure
#empty intercept-only model
model1 <- gls(walkDistance ~ 1,
                  data = walkDat,
                  method = "REML",
                  na.action = "na.exclude")
summary(model1)
```

```
## Generalized least squares fit by REML
##   Model: walkDistance ~ 1
##   Data: walkDat
##        AIC      BIC    logLik
##   1944.217 1950.342 -970.1083
##
## Coefficients:
##                Value Std.Error  t-value p-value
## (Intercept) 467.6419  8.762616 53.36784       0
##
## Standardized residuals:
##         Min          Q1         Med          Q3         Max
## -2.77333055 -0.67202746 -0.03296082  0.77140227  2.06093905
##
## Residual standard error: 110.4924
## Degrees of freedom: 159 total; 158 residual
```

```r
#random intercept model
model2 <- lme(walkDistance ~ 1,
                 data = walkDat,
                 method = "REML",
                 random = ~1|id,
                 na.action = "na.exclude")
summary(model2)
```

```
## Linear mixed-effects model fit by REML
##   Data: walkDat
##        AIC      BIC    logLik
##   1843.766 1852.954 -918.8829
##
## Random effects:
##  Formula: ~1 | id
##         (Intercept) Residual
## StdDev:    96.63076 48.91906
##
## Fixed effects:  walkDistance ~ 1
##                Value Std.Error DF  t-value p-value
## (Intercept) 464.1413  12.16244 87 38.16184       0
##
## Standardized Within-Group Residuals:
##         Min          Q1         Med          Q3         Max
## -3.71367132 -0.33523171 -0.04033151  0.43462573  2.57381630
##
## Number of Observations: 159
## Number of Groups: 72
```

```
anova(model1, model2)
```

```
##        Model df      AIC      BIC    logLik   Test  L.Ratio p-value
## model1     1  2 1944.217 1950.342 -970.1083
## model2     2  3 1843.766 1852.954 -918.8829 1 vs 2 102.4509  <.0001
```

```
icc(model2)
```

```
## [1] 0.8
```

```
#add fixed effects for time, fixed linear time model
model3 <- lme(walkDistance ~ bpiTotalBase + baseToEndOfTx*treatment + endOfTxToFu*treatment,
                 data = walkDat,
                 method = "REML",
                 random = ~1|id,
                 na.action = "na.exclude")
summary(model3)
```

```
## Linear mixed-effects model fit by REML
##   Data: walkDat
##        AIC      BIC    logLik
##   1799.034 1826.189 -890.5169
##
## Random effects:
##  Formula: ~1 | id
##         (Intercept) Residual
## StdDev:    90.32627  46.2563
##
## Fixed effects:  walkDistance ~ bpiTotalBase + baseToEndOfTx * treatment + endOfTxToFu *      treatme
##                             Value Std.Error DF   t-value p-value
## (Intercept)              554.1132  35.67217 83 15.533487  0.0000
## bpiTotalBase             -19.9208   5.96008 68 -3.342373  0.0014
## baseToEndOfTx              4.5725   1.22616 83  3.729135  0.0004
## treatment                -11.3467  24.71690 68 -0.459068  0.6476
## endOfTxToFu                1.1637   0.50770 83  2.292116  0.0244
## baseToEndOfTx:treatment   -2.9637   1.93028 83 -1.535393  0.1285
## treatment:endOfTxToFu     -1.2490   0.82108 83 -1.521137  0.1320
##  Correlation:
##                          (Intr) bpTtlB bsTEOT trtmnt enOTTF bTEOT:
## bpiTotalBase             -0.881
## baseToEndOfTx            -0.150 -0.002
## treatment                -0.492  0.191  0.220
## endOfTxToFu              -0.141  0.002  0.432  0.201
## baseToEndOfTx:treatment   0.116 -0.022 -0.635 -0.276 -0.274
## treatment:endOfTxToFu     0.099 -0.015 -0.267 -0.243 -0.618  0.362
##
## Standardized Within-Group Residuals:
##         Min          Q1         Med          Q3         Max
## -3.28723522 -0.37521217 -0.00938461  0.37166402  2.65955945
##
## Number of Observations: 158
## Number of Groups: 71
```

```r
#yoga only
yogaWalkDat <- walkDat %>% filter(treatment == 0)

yogaWalkDat <- as.data.frame(yogaWalkDat)

model4 <- lme(walkDistance ~ bpiTotalBase + baseToEndOfTx + endOfTxToFu,
                 data = yogaWalkDat,
                 method = "REML",
                 random = ~1|id,
                 na.action = "na.exclude")
summary(model4)
```

```
## Linear mixed-effects model fit by REML
##   Data: yogaWalkDat
##        AIC      BIC    logLik
##   1047.515 1062.311 -517.7577
##
## Random effects:
##  Formula: ~1 | id
##         (Intercept) Residual
## StdDev:    94.28421 51.57005
##
## Fixed effects:  walkDistance ~ bpiTotalBase + baseToEndOfTx + endOfTxToFu
##                  Value Std.Error DF   t-value p-value
## (Intercept)   561.7037  47.24390 52 11.889443  0.0000
## bpiTotalBase  -21.3662   8.29249 35 -2.576574  0.0144
## baseToEndOfTx   4.5729   1.36495 52  3.350259  0.0015
## endOfTxToFu     1.1711   0.56516 52  2.072103  0.0432
##  Correlation:
##               (Intr) bpTtlB bsTEOT
## bpiTotalBase  -0.925
## baseToEndOfTx -0.125 -0.003
## endOfTxToFu   -0.120  0.003  0.430
##
## Standardized Within-Group Residuals:
##         Min          Q1         Med          Q3         Max
## -2.92517289 -0.36663255  0.03785548  0.36218036  2.40543118
##
## Number of Observations: 91
## Number of Groups: 37
```

```r
#cbt only
cbtWalkDat <- walkDat %>% filter(treatment == 1)

cbtWalkDat <- as.data.frame(cbtWalkDat)

model5 <- lme(walkDistance ~ bpiTotalBase + baseToEndOfTx + endOfTxToFu,
                 data = cbtWalkDat,
                 method = "REML",
                 random = ~1|id,
                 na.action = "na.exclude")
summary(model5)
```

```
## Linear mixed-effects model fit by REML
##   Data: cbtWalkDat
##        AIC      BIC    logLik
##   745.4511 758.3099 -366.7255
##
## Random effects:
##  Formula: ~1 | id
##         (Intercept) Residual
## StdDev:     88.2949 35.79427
##
## Fixed effects:  walkDistance ~ bpiTotalBase + baseToEndOfTx + endOfTxToFu
##                   Value Std.Error DF   t-value p-value
## (Intercept)   532.6786  42.13281 32 12.642845  0.0000
## bpiTotalBase  -17.6689   8.66934 32 -2.038086  0.0499
## baseToEndOfTx   1.5224   1.16224 31  1.309870  0.1999
## endOfTxToFu    -0.0875   0.50267 31 -0.174014  0.8630
##  Correlation:
##               (Intr) bpTtlB bsTEOT
## bpiTotalBase  -0.922
## baseToEndOfTx -0.042 -0.038
## endOfTxToFu   -0.047 -0.022  0.323
##
## Standardized Within-Group Residuals:
##         Min          Q1         Med          Q3         Max
## -2.13086124 -0.43569739 -0.04030605  0.38209616  1.75415844
##
## Number of Observations: 67
## Number of Groups: 34
```

```r
#cohen's d
#https://stats.stackexchange.com/questions/257985/how-can-i-derive-effect-sizes-in-lme4-and-describe-th
nlmeCohenD <- function(meanDif, model) {
        cohenD <- meanDif / (sqrt(as.numeric(VarCorr(model)[1,1]) +
                                as.numeric(VarCorr(model)[2,1])))
        cohenD <- as.numeric(cohenD)
        return(cohenD)
}

walkCohenDat <- walkDat %>%
        group_by(treatment, week) %>%
        summarise(meanWalkDist = mean(walkDistance, na.rm = T)) %>%
        ungroup() %>%
        group_by(week) %>%
        mutate(meanDif = last(meanWalkDist) - first(meanWalkDist)) %>%
        ungroup() %>%
        mutate(walkDistCohenD = purrr::map_dbl(meanDif, nlmeCohenD, model3)) %>%
        filter(week >= 10) %>%
        select(treatment, week, meanWalkDist, walkDistCohenD)
```

```
## `summarise()` has grouped output by 'treatment'. You can override using the
## `.groups` argument.
```

```
#clinical improvement (> 30.5 per https://onlinelibrary.wiley.com/doi/10.1111/jep.12629)
walkDat %>%
        select(id, treatment, week, walkDistance) %>%
        filter(week == 0 | week == 34) %>%
        group_by(id) %>%
        mutate(walkDistChange = last(walkDistance) - first(walkDistance)) %>%
        ungroup() %>%
        select(id, treatment, walkDistChange) %>%
        distinct() %>%
        mutate(clinImp = ifelse(walkDistChange >= 30.5, 1, 0)) %>%
        group_by(treatment) %>%
        summarise((sum(clinImp, na.rm = T) / sum(!is.na(clinImp))),
                  sum(clinImp, na.rm = T),
                  sum(!is.na(clinImp)))
```

```
## # A tibble: 2 x 4
##   treatment `(sum(clinImp, na.rm = T)/sum(!is.na(clinImp)))` sum(clinI~1 sum(!~2
##       <dbl>                                            <dbl>       <dbl>   <int>
## 1         0                                            0.458          11      24
## 2         1                                            0.333           5      15
## # ... with abbreviated variable names 1: `sum(clinImp, na.rm = T)`,
## #   2: `sum(!is.na(clinImp))`
```

```
chiSquareWalkDat <- walkDat %>%
        select(id, treatment, week, walkDistance) %>%
        filter(week == 0 | week == 34) %>%
        group_by(id) %>%
        mutate(walkDistChange = last(walkDistance) - first(walkDistance)) %>%
        ungroup() %>%
        select(id, treatment, walkDistChange) %>%
        distinct() %>%
        mutate(clinImp = ifelse(walkDistChange >= 30.5, 1, 0))

fisher.test(table(chiSquareWalkDat$treatment, chiSquareWalkDat$clinImp))
```

```
##
##  Fisher's Exact Test for Count Data
##
## data:  table(chiSquareWalkDat$treatment, chiSquareWalkDat$clinImp)
## p-value = 0.5166
## alternative hypothesis: true odds ratio is not equal to 1
## 95 percent confidence interval:
##  0.1210385 2.6853849
## sample estimates:
## odds ratio
##  0.5989188
```

```
#plot model 3
fatigueFig <- plot(ggpredict(model3, terms = c("endOfTxToFu", "treatment"), ci.lvl = NA), use.theme = F
     color = "bw", connect.lines = T) +
                                        scale_x_continuous(breaks = c(0, 24)) +
                                        theme(axis.text=element_text(size = 14),
```

```
                                                 axis.title=element_text(size = 16, face="bold"),
                                                 strip.text.x = element_text(size = 16)) +
                                           ylim(400,550) + geom_point() +
        scale_color_manual(name = "",
                           labels = c("Yoga", "CBT"),
                           values = c("#000000", "#000000"),
                           guide = guide_legend(reverse = F)) +
        scale_linetype_manual(name = "",
                           labels = c("Yoga", "CBT"),
                           values = c("dashed", "solid"),
                           guide = guide_legend(reverse = F)) +
        jtools::theme_apa(legend.pos = "top", x.font.size = 12, y.font.size = 12,
                          facet.title.size = 12, legend.use.title = F) +
        scale_x_continuous(breaks=c(0, 8, 16, 24),
                       labels=c("EOT", "2-months", "4-months", "6-months")) +
        theme(axis.text.x = element_text(size = 12),
              axis.text.y = element_text(size = 12),
              panel.border = element_blank()) +
        labs(tag = "D", title = "") +
        xlab("") + ylab("Fatigue (meters)") +
        theme(axis.line = element_line(color = "black"))
```

```
## Scale for y is already present.
## Adding another scale for y, which will replace the existing scale.
## Scale for colour is already present.
## Adding another scale for colour, which will replace the existing scale.
## Scale for x is already present.
## Adding another scale for x, which will replace the existing scale.
```

Check Assumptions: Model 3

```
#check for independence and normality of within-group errors (participants)
plot(model3, resid(., type="p") ~ fitted(.), abline=0)
```

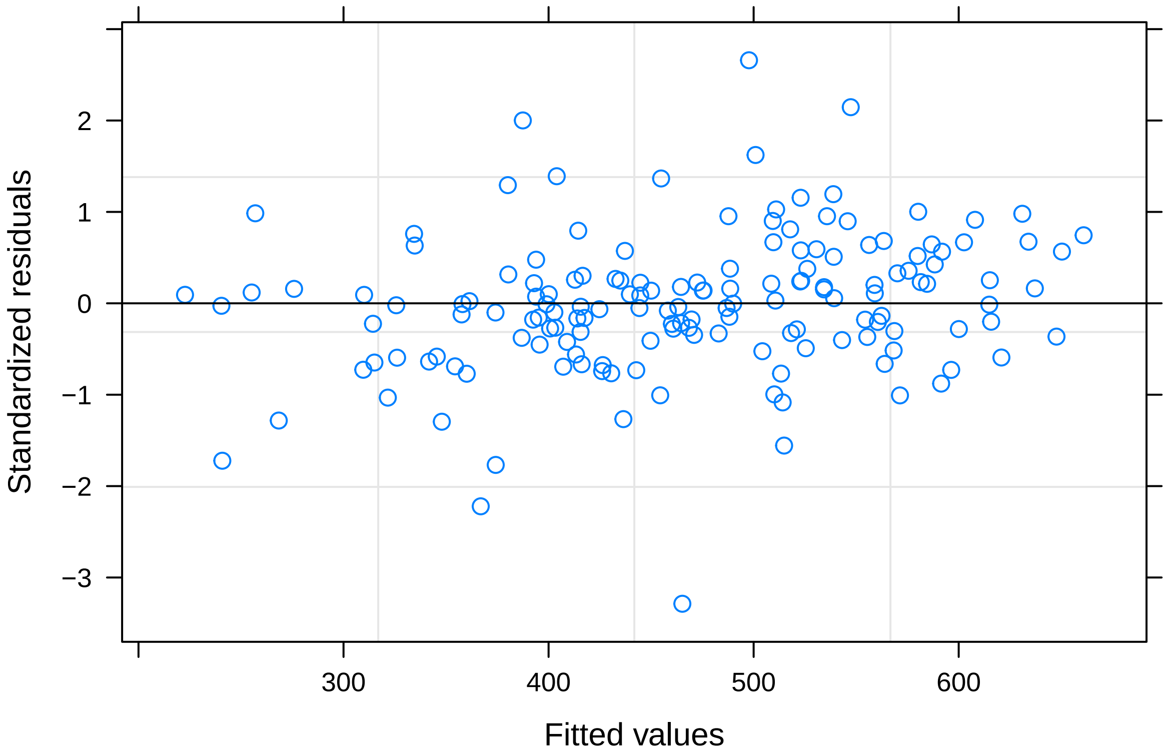


```
plot(model3, id~resid(.), abline = 0 )
```

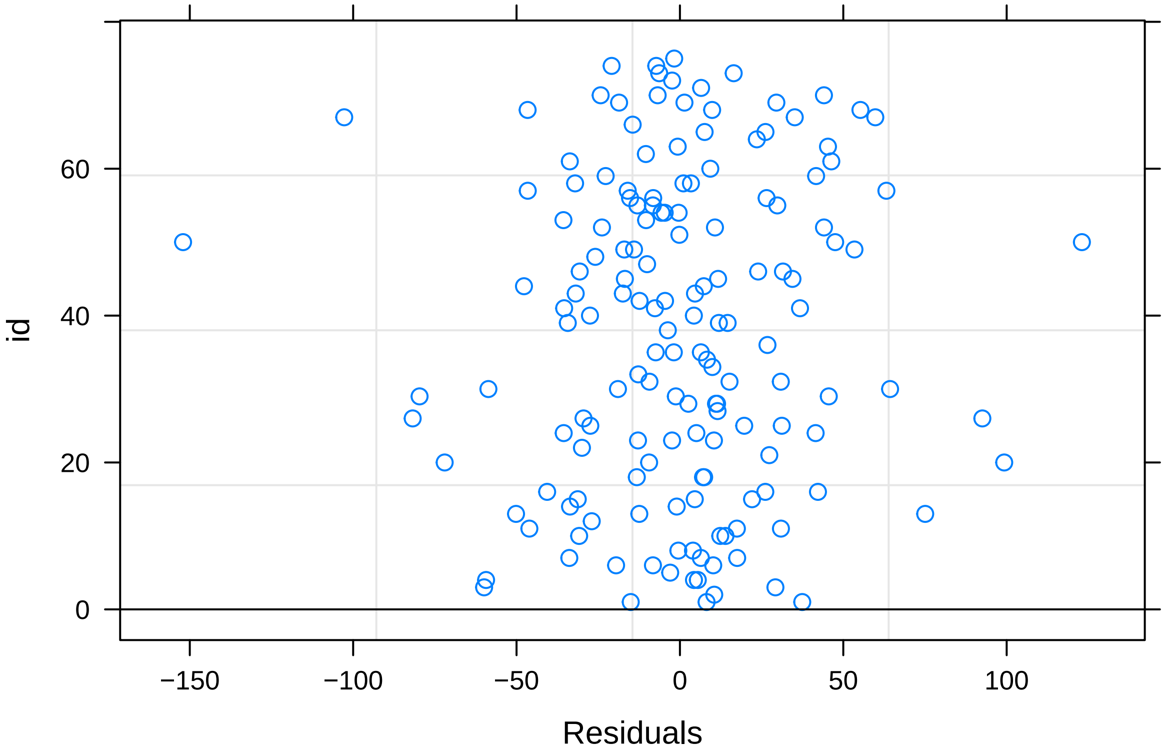


```
plot(model3, resid(., type = "p") ~ fitted(.) | week, id = 0.05, adj = -0.3 )
```

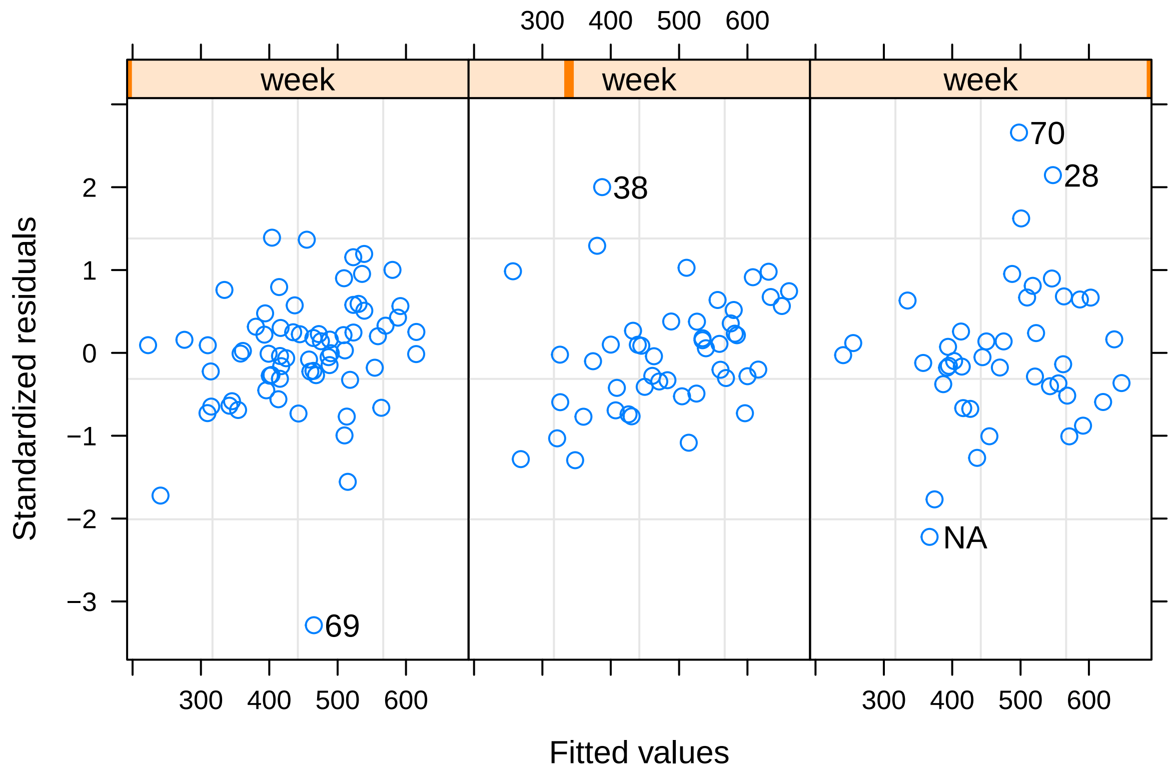


```
plot(model3, walkDistance ~ fitted(.) | week, id = 0.05, adj = -0.3 )
```

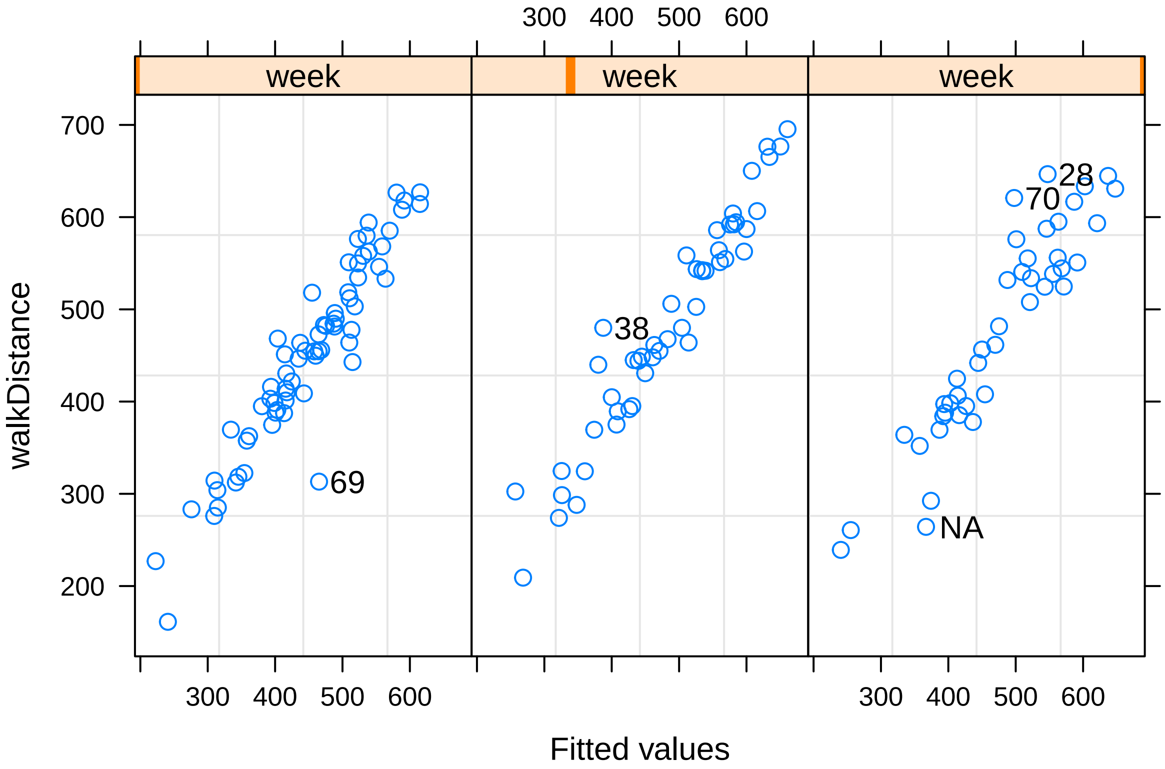

```
plot(model3, walkDistance ~ fitted(.), id=.05, adj=-0.3)
```

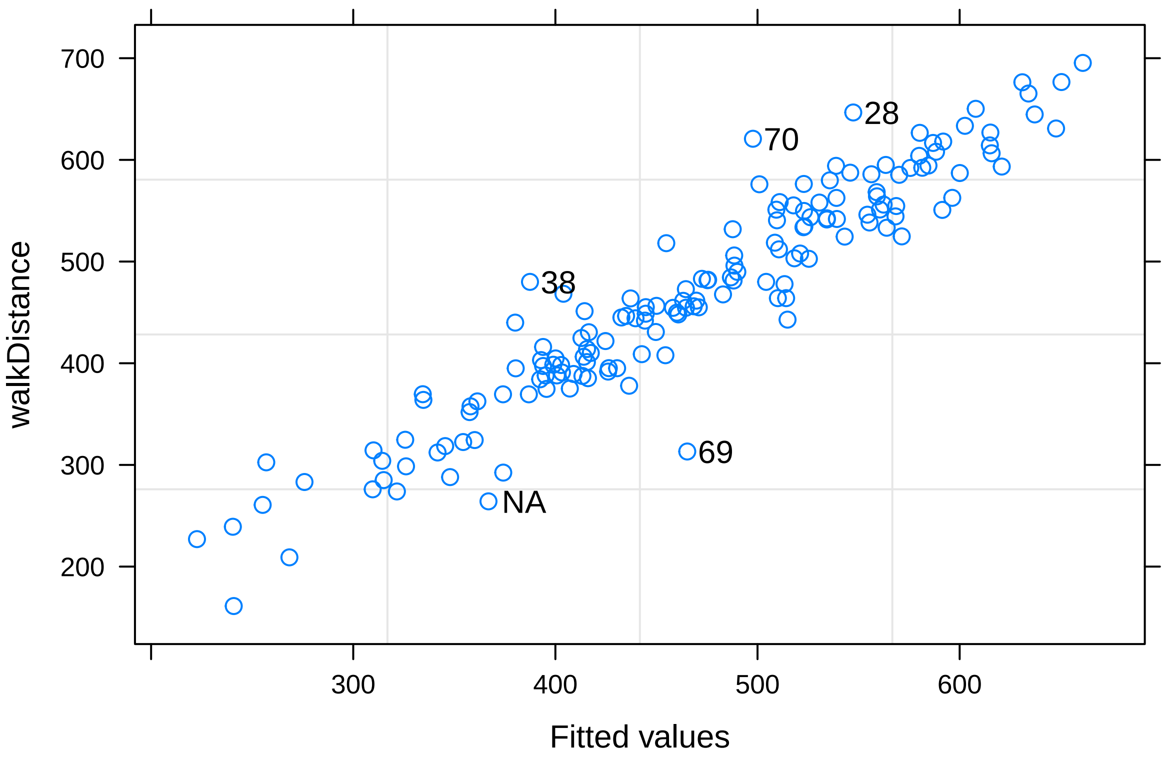


```
hist(residuals(model1), breaks=20, id=.05)
## Warning in plot.window(xlim, ylim, "", ...): "id" is not a graphical parameter
## Warning in title(main = main, sub = sub, xlab = xlab, ylab = ylab, ...): "id"
## is not a graphical parameter
## Warning in axis(1, ...): "id" is not a graphical parameter
## Warning in axis(2, at = yt, ...): "id" is not a graphical parameter
```

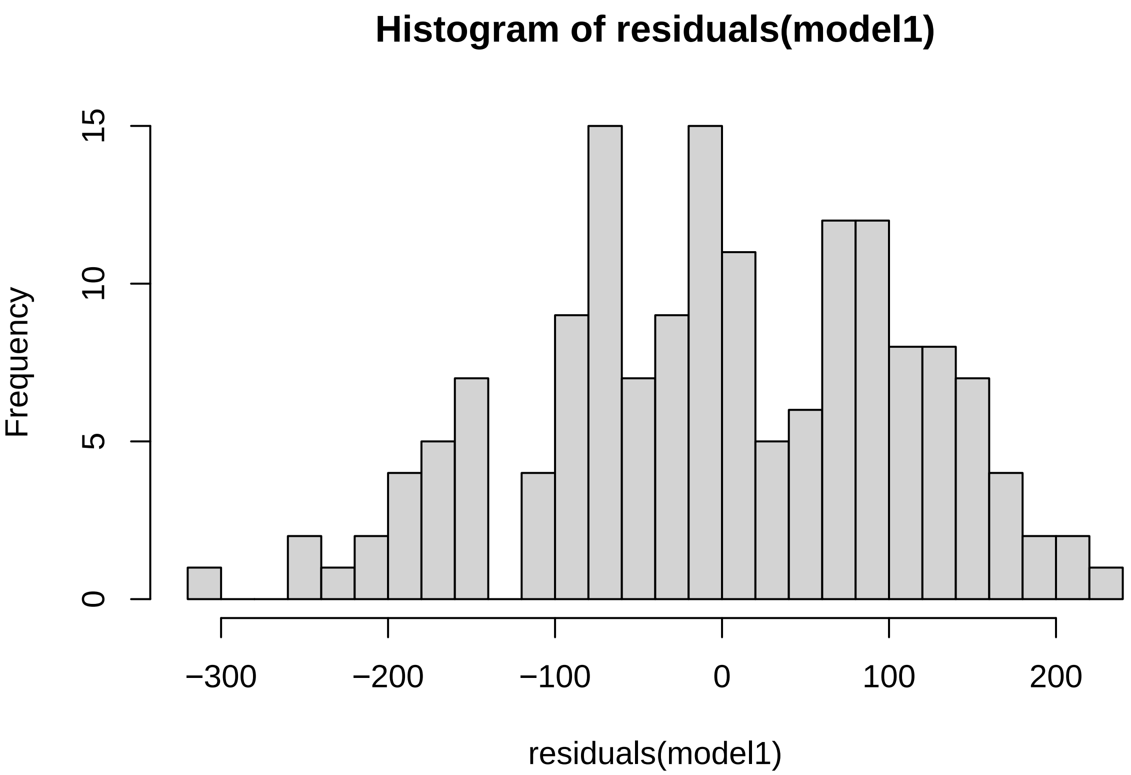


```
qqnorm(model3)
```

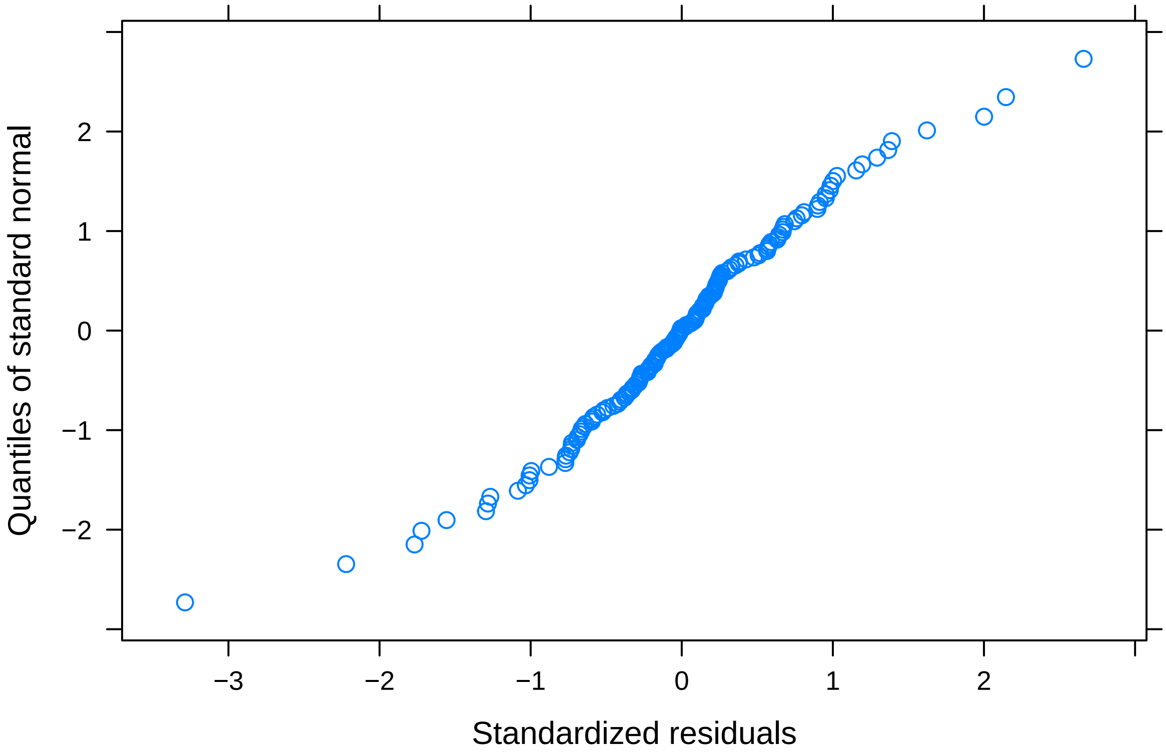

```
#check for normality and independence of random effects
qqnorm(model3, ~ranef(.), id=0.10, cex=0.7)
```

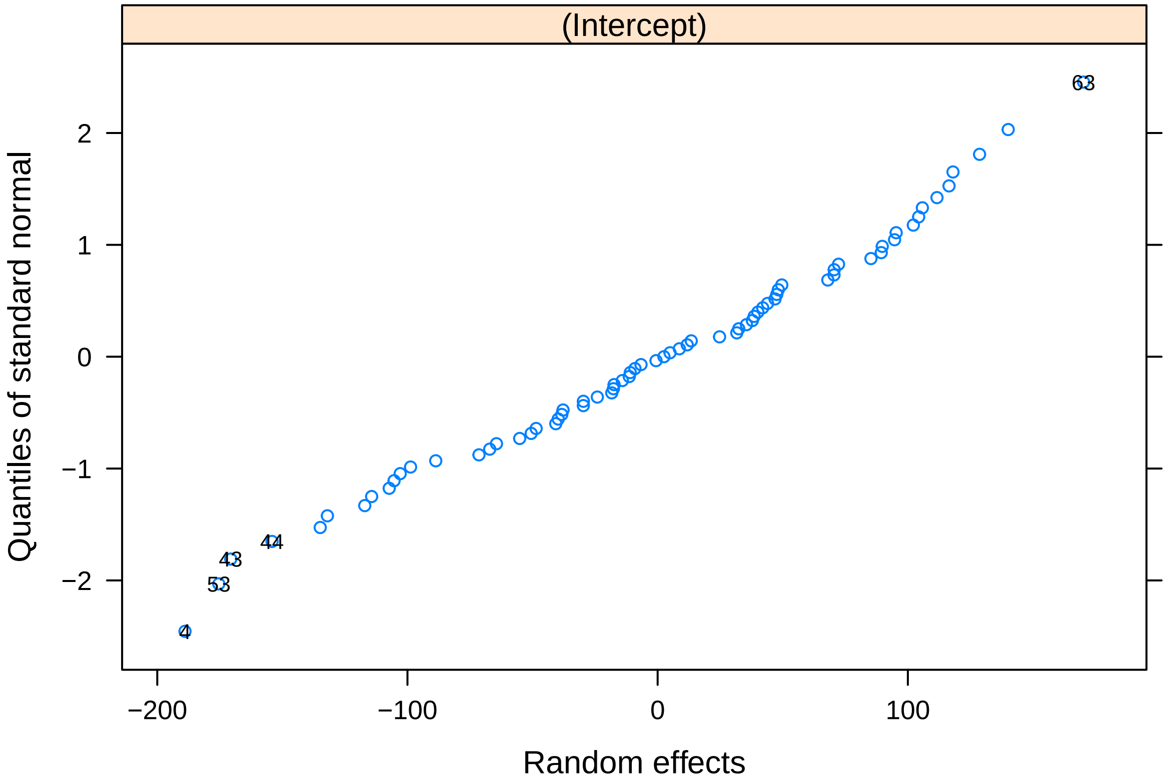

Secondary Vars: HAM-D

```
hamdDat <- analyzeMe %>%
        select(ID, treatment, bpiTotalBase = BPImrb, hamdtotrb, hamdtotw10, hamdtotw34) %>%
        rowid_to_column(., "id") %>% select(-ID) %>%
        pivot_longer(4:6, names_to = "week", values_to = "hamdScore") %>%
        mutate(week = case_when(
                week == "hamdtotrb" ~ 0,
                week == "hamdtotw10" ~ 10,
                week == "hamdtotw34" ~ 34
        )) %>%
        mutate(baseToEndOfTx = ifelse(week <= 10, week, 1)) %>%
        mutate(endOfTxToFu = case_when(
                week == 0 ~ 0,
                week == 10 ~ 0,
                week == 34 ~ 24
        ))

cor.test(analyzeMe$BPImrb, analyzeMe$hamdtotrb)
```

```
##
```

```
##  Pearson's product-moment correlation
##
## data:  analyzeMe$BPImrb and analyzeMe$hamdtotrb
## t = 7.1822, df = 68, p-value = 6.657e-10
## alternative hypothesis: true correlation is not equal to 0
## 95 percent confidence interval:
##  0.4987828 0.7725330
## sample estimates:
##       cor
## 0.6567814
```

```
hamdDat %>%
        group_by(treatment, week) %>%
        summarise(hamdMean = mean(hamdScore, na.rm = T)) %>%
        ggplot(., aes(week, hamdMean, color = as.factor(treatment))) +
        geom_point() +
        geom_line() +
        ylim(0, 15)
```

```
## `summarise()` has grouped output by 'treatment'. You can override using the
## `.groups` argument.
```

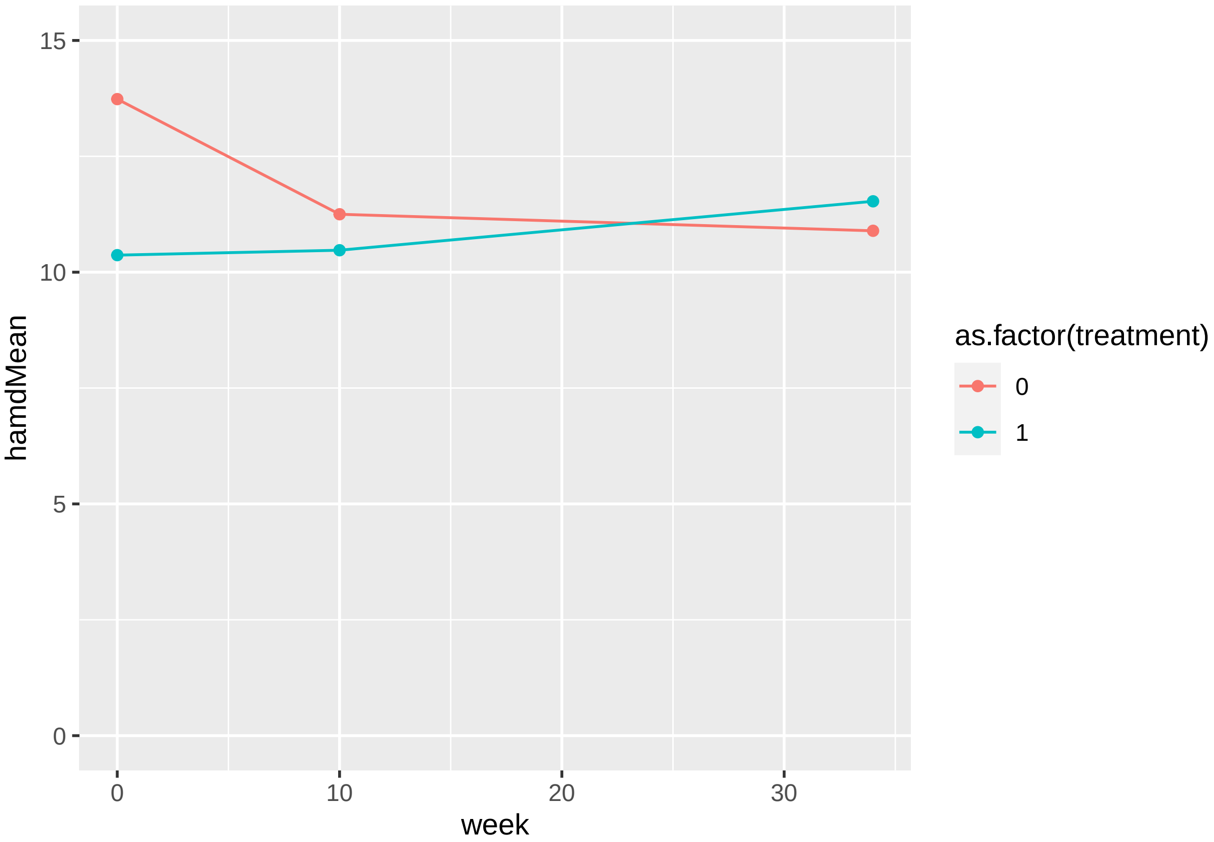


```
###build models: step-up procedure
#empty intercept-only model
model1 <- gls(hamdScore ~ 1,
                  data = hamdDat,
```

```r
                  method = "REML",
                  na.action = "na.exclude")
summary(model1)
```

```
## Generalized least squares fit by REML
##   Model: hamdScore ~ 1
##   Data: hamdDat
##        AIC      BIC    logLik
##   1153.041 1159.265 -574.5207
##
## Coefficients:
##                Value Std.Error  t-value p-value
## (Intercept) 11.48086 0.5872038 19.55175       0
##
## Standardized residuals:
##         Min          Q1         Med          Q3         Max
## -1.51295999 -0.85405484 -0.06336866  0.66142701  2.70403298
##
## Residual standard error: 7.588346
## Degrees of freedom: 167 total; 166 residual
```

```r
#random intercept model
model2 <- lme(hamdScore ~ 1,
                 data = hamdDat,
                 method = "REML",
                 random = ~1|id,
                 na.action = "na.exclude")
summary(model2)
```

```
## Linear mixed-effects model fit by REML
##   Data: hamdDat
##        AIC      BIC    logLik
##   1082.937 1092.273 -538.4685
##
## Random effects:
##  Formula: ~1 | id
##         (Intercept) Residual
## StdDev:    6.461437 4.088325
##
## Fixed effects:  hamdScore ~ 1
##                Value Std.Error DF  t-value p-value
## (Intercept) 11.45024 0.8264987 93 13.85392       0
##
## Standardized Within-Group Residuals:
##         Min          Q1         Med          Q3         Max
## -2.47304612 -0.51395209 -0.05597818  0.59672746  2.17433403
##
## Number of Observations: 167
## Number of Groups: 74
```

```r
anova(model1, model2)
```

```
##        Model df      AIC      BIC    logLik   Test  L.Ratio p-value
## model1     1  2 1153.041 1159.265 -574.5207
## model2     2  3 1082.937 1092.273 -538.4685 1 vs 2 72.10429  <.0001
```

```r
icc(model2)
```

```
## [1] 0.71
```

```r
model3 <- lme(hamdScore ~ bpiTotalBase + baseToEndOfTx*treatment + endOfTxToFu*treatment,
              data = hamdDat,
              method = "REML",
              na.action = "na.exclude",
              random = ~1 |id)

summary(model3)
```

```
## Linear mixed-effects model fit by REML
##   Data: hamdDat
##        AIC     BIC  logLik
##   1055.54 1083.16 -518.77
##
## Random effects:
##  Formula: ~1 | id
##         (Intercept) Residual
## StdDev:    4.610016 4.075461
##
## Fixed effects:  hamdScore ~ bpiTotalBase + baseToEndOfTx * treatment + endOfTxToFu *      treatment
##                              Value Std.Error DF   t-value p-value
## (Intercept)              0.8807765 2.0469259 89  0.430292  0.6680
## bpiTotalBase             2.2942018 0.3275072 70  7.005042  0.0000
## baseToEndOfTx           -0.1560607 0.1029105 89 -1.516471  0.1329
## treatment               -0.7721900 1.4905412 70 -0.518060  0.6060
## endOfTxToFu             -0.0831189 0.0430754 89 -1.929613  0.0568
## baseToEndOfTx:treatment  0.0780568 0.1620623 89  0.481647  0.6312
## treatment:endOfTxToFu    0.1274511 0.0679531 89  1.875575  0.0640
##  Correlation:
##                         (Intr) bpTtlB bsTEOT trtmnt enOTTF bTEOT:
## bpiTotalBase            -0.870
## baseToEndOfTx           -0.246  0.021
## treatment               -0.508  0.200  0.317
## endOfTxToFu             -0.221  0.017  0.411  0.286
## baseToEndOfTx:treatment  0.183 -0.044 -0.636 -0.410 -0.262
## treatment:endOfTxToFu    0.157 -0.030 -0.261 -0.366 -0.634  0.353
##
## Standardized Within-Group Residuals:
##         Min          Q1         Med          Q3         Max
## -2.25377155 -0.48720171  0.01316624  0.50787551  2.04021989
##
## Number of Observations: 166
## Number of Groups: 73
```

```r
#cohen's d
#https://stats.stackexchange.com/questions/257985/how-can-i-derive-effect-sizes-in-lme4-and-describe-th
hamdCohenDat <- hamdDat %>%
        group_by(treatment, week) %>%
        summarise(meanHamd = mean(hamdScore, na.rm = T)) %>%
        ungroup() %>%
        group_by(week) %>%
        mutate(meanDif = last(meanHamd) - first(meanHamd)) %>%
        ungroup() %>%
        mutate(hamdCohenD = purrr::map_dbl(meanDif, nlmeCohenD, model3)) %>%
        filter(week >= 10) %>%
        select(treatment, week, meanHamd, hamdCohenD)
```

```
## `summarise()` has grouped output by 'treatment'. You can override using the
## `.groups` argument.
```

Secondard Vars: SF-36 Health-Related Quality of Life

```r
sfGeneralDat <- analyzeMe %>%
        select(ID, treatment, bpiTotalBase = BPImrb, SFghrb, SFghw10, SFghw34) %>%
        rowid_to_column(., "id") %>% select(-ID) %>%
        pivot_longer(4:6, names_to = "week", values_to = "generalHealthScore") %>%
        mutate(week = case_when(
                week == "SFghrb" ~ 0,
                week == "SFghw10" ~ 10,
                week == "SFghw34" ~ 34
        )) %>%
        mutate(baseToEndOfTx = ifelse(week <= 10, week, 1)) %>%
        mutate(endOfTxToFu = case_when(
                week == 0 ~ 0,
                week == 10 ~ 0,
                week == 34 ~ 24
        ))

sfGeneralDat %>%
        group_by(treatment, week) %>%
        summarise(genHealthMean = mean(generalHealthScore, na.rm = T)) %>%
        ggplot(., aes(week, genHealthMean, color = as.factor(treatment))) +
        geom_point() +
        geom_line() +
        ylim(0, 60)
```

```
## `summarise()` has grouped output by 'treatment'. You can override using the
## `.groups` argument.
```

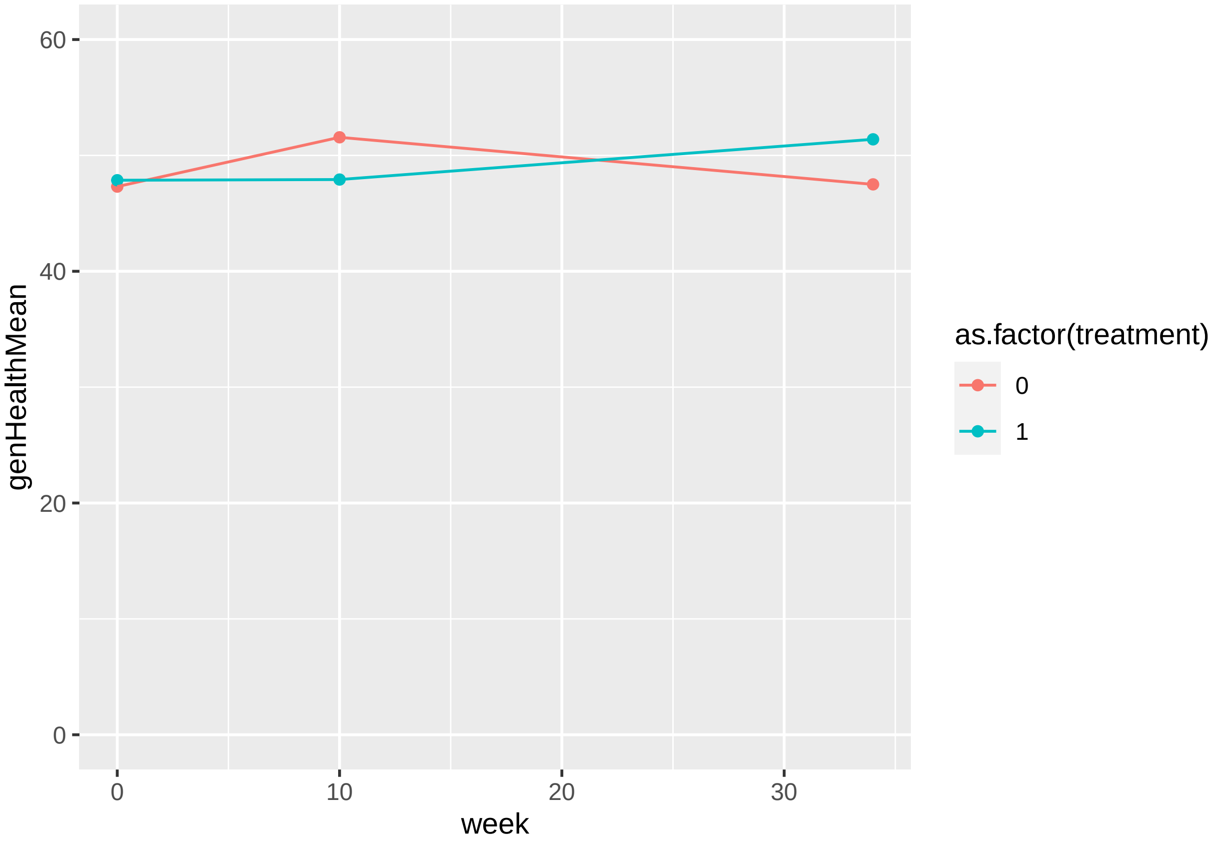


```
cor.test(analyzeMe$BPI9mrb, analyzeMe$SFghrb)
```

```
##
##  Pearson's product-moment correlation
##
## data:  analyzeMe$BPI9mrb and analyzeMe$SFghrb
## t = -4.3715, df = 72, p-value = 4.079e-05
## alternative hypothesis: true correlation is not equal to 0
## 95 percent confidence interval:
##  -0.6214482 -0.2563065
## sample estimates:
##        cor
## -0.4579831
```

```
###build models: step-up procedure
#empty intercept-only model
model1 <- gls(generalHealthScore ~ 1,
                  data = sfGeneralDat,
                  method = "REML",
                  na.action = "na.exclude")
summary(model1)
```

```
## Generalized least squares fit by REML
##   Model: generalHealthScore ~ 1
##   Data: sfGeneralDat
##        AIC      BIC    logLik
```

```
##   1524.698 1530.958 -760.3491
##
## Coefficients:
##             Value Std.Error t-value p-value
## (Intercept) 48.75  1.643905  29.655       0
##
## Standardized residuals:
##        Min         Q1        Med         Q3        Max
## -2.2744346 -0.6415072 -0.1749565  0.5248695  2.1577970
##
## Residual standard error: 21.4339
## Degrees of freedom: 170 total; 169 residual
```

```r
#random intercept model
model2 <- lme(generalHealthScore ~ 1,
                 data = sfGeneralDat,
                 method = "REML",
                 random = ~1|id,
                 na.action = "na.exclude")
summary(model2)
```

```
## Linear mixed-effects model fit by REML
##   Data: sfGeneralDat
##        AIC     BIC    logLik
##   1429.091 1438.48 -711.5453
##
## Random effects:
##  Formula: ~1 | id
##         (Intercept) Residual
## StdDev:    19.19201 10.07885
##
## Fixed effects:  generalHealthScore ~ 1
##                Value Std.Error DF  t-value p-value
## (Intercept) 48.33713  2.387311 96 20.24752       0
##
## Standardized Within-Group Residuals:
##        Min         Q1        Med         Q3        Max
## -1.9443205 -0.4710847  0.0390741  0.5311113  1.8464806
##
## Number of Observations: 170
## Number of Groups: 74
```

```r
anova(model1, model2)
```

```
##        Model df      AIC      BIC    logLik   Test  L.Ratio p-value
## model1     1  2 1524.698 1530.958 -760.3491
## model2     2  3 1429.091 1438.480 -711.5453 1 vs 2 97.60756  <.0001
```

```r
icc(model2)
```

```
## [1] 0.78
```

```r
model3 <- lme(generalHealthScore ~ bpiTotalBase + baseToEndOfTx*treatment + endOfTxToFu*treatment,
              data = sfGeneralDat,
              method = "REML",
              na.action = "na.exclude",
              random = ~1 |id)

summary(model3)
```

```
## Linear mixed-effects model fit by REML
##   Data: sfGeneralDat
##        AIC     BIC    logLik
##   1418.846 1446.69 -700.4231
##
## Random effects:
##  Formula: ~1 | id
##         (Intercept) Residual
## StdDev:    16.83019 10.09812
##
## Fixed effects:  generalHealthScore ~ bpiTotalBase + baseToEndOfTx * treatment +      endOfTxToFu * t
##                             Value Std.Error DF   t-value p-value
## (Intercept)              74.27859  6.721101 92 11.051551  0.0000
## bpiTotalBase             -4.98136  1.097268 71 -4.539785  0.0000
## baseToEndOfTx             0.22716  0.249450 92  0.910639  0.3649
## treatment                -3.99712  4.678366 71 -0.854384  0.3958
## endOfTxToFu              -0.06949  0.104807 92 -0.663016  0.5090
## baseToEndOfTx:treatment   0.14321  0.407470 92  0.351465  0.7260
## treatment:endOfTxToFu     0.19282  0.166338 92  1.159193  0.2494
##  Correlation:
##                         (Intr) bpTtlB bsTEOT trtmnt enOTTF bTEOT:
## bpiTotalBase            -0.884
## baseToEndOfTx           -0.165  0.010
## treatment               -0.503  0.214  0.226
## endOfTxToFu             -0.155  0.018  0.385  0.204
## baseToEndOfTx:treatment  0.130 -0.039 -0.613 -0.298 -0.236
## treatment:endOfTxToFu    0.103 -0.017 -0.242 -0.270 -0.630  0.347
##
## Standardized Within-Group Residuals:
##         Min          Q1         Med          Q3         Max
## -2.00555972 -0.46643402 -0.01150429  0.51221009  1.94162015
##
## Number of Observations: 170
## Number of Groups: 74
```

```r
#cohen's d
#https://stats.stackexchange.com/questions/257985/how-can-i-derive-effect-sizes-in-lme4-and-describe-th
sfGeneralCohenDat <- sfGeneralDat %>%
        group_by(treatment, week) %>%
        summarise(meanSfGen = mean(generalHealthScore, na.rm = T)) %>%
        ungroup() %>%
        group_by(week) %>%
        mutate(meanDif = last(meanSfGen) - first(meanSfGen)) %>%
        ungroup() %>%
        mutate(sfGenCohenD = purrr::map_dbl(meanDif, nlmeCohenD, model3)) %>%
```

```
        filter(week >= 10) %>%
        select(treatment, week, meanSfGen, sfGenCohenD)
```

```
## `summarise()` has grouped output by 'treatment'. You can override using the
## `.groups` argument.
```

Secondard Vars: Autonomic Symptom Severity

```
autoSxDat <- analyzeMe %>%
        select(ID, treatment, bpiTotalBase = BPImrb, comtotrb, comtotw10, comtotw34) %>%
        rowid_to_column(., "id") %>% select(-ID) %>%
        pivot_longer(4:6, names_to = "week", values_to = "autoSxScore") %>%
        mutate(week = case_when(
                week == "comtotrb" ~ 0,
                week == "comtotw10" ~ 10,
                week == "comtotw34" ~ 34
        )) %>%
        mutate(baseToEndOfTx = ifelse(week <= 10, week, 1)) %>%
        mutate(endOfTxToFu = case_when(
                week == 0 ~ 0,
                week == 10 ~ 0,
                week == 34 ~ 24
        ))

autoSxDat %>%
        group_by(treatment, week) %>%
        summarise(autoSxMean = mean(autoSxScore, na.rm = T)) %>%
        ggplot(., aes(week, autoSxMean, color = as.factor(treatment))) +
        geom_point() +
        geom_line() +
        ylim(0, 60)
```

```
## `summarise()` has grouped output by 'treatment'. You can override using the
## `.groups` argument.
```

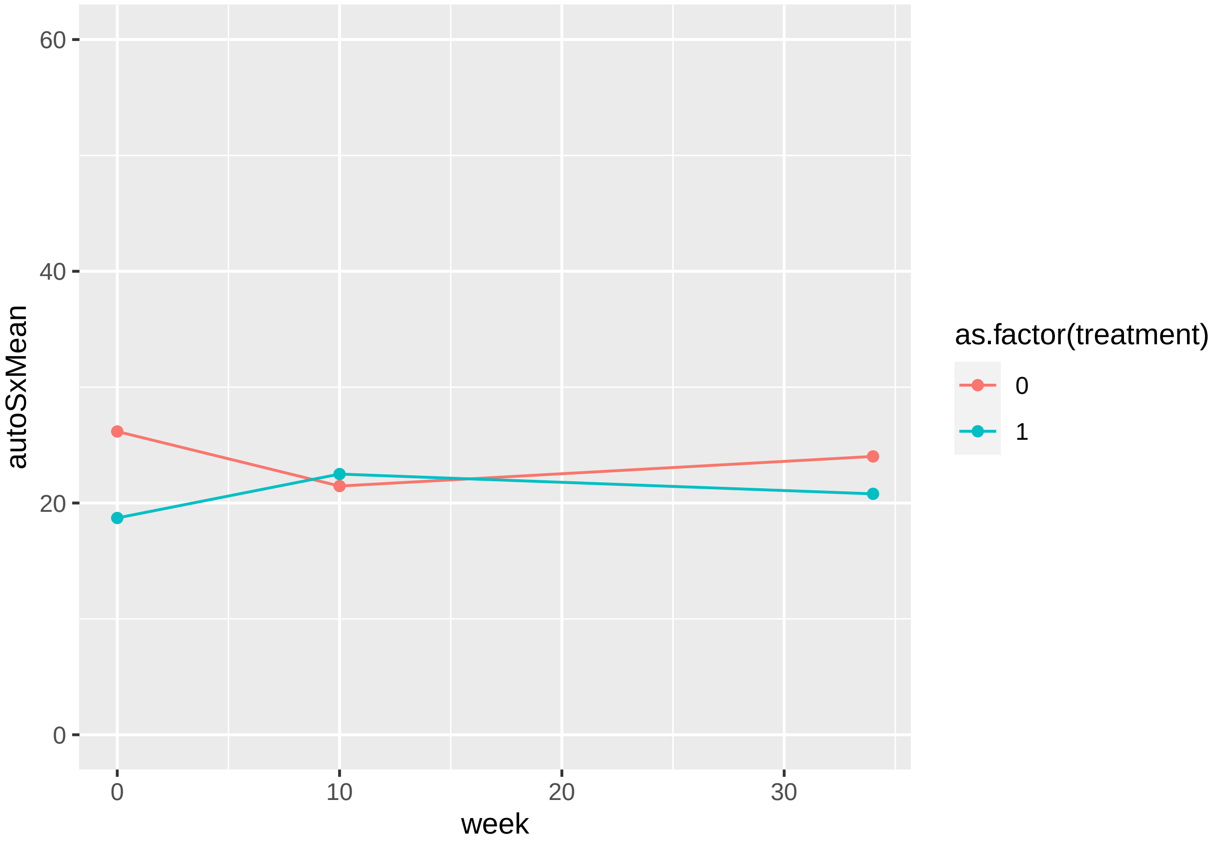


```
cor.test(analyzeMe$BPImrb, analyzeMe$comtotrb)
```

```
##
##  Pearson's product-moment correlation
##
## data:  analyzeMe$BPImrb and analyzeMe$comtotrb
## t = 3.9981, df = 63, p-value = 0.0001701
## alternative hypothesis: true correlation is not equal to 0
## 95 percent confidence interval:
##  0.2313515 0.6251705
## sample estimates:
##       cor
## 0.4498663
```

```
###build models: step-up procedure
#empty intercept-only model
model1 <- gls(autoSxScore ~ 1,
                  data = autoSxDat,
                  method = "REML",
                  na.action = "na.exclude")
summary(model1)
```

```
## Generalized least squares fit by REML
##   Model: autoSxScore ~ 1
##   Data: autoSxDat
##        AIC      BIC    logLik
```

```
##   1328.656 1334.769 -662.3281
##
## Coefficients:
##                Value Std.Error  t-value p-value
## (Intercept) 22.57718  1.286971 17.54288       0
##
## Standardized residuals:
##        Min         Q1        Med         Q3        Max
## -1.3956365 -0.8495930 -0.1063262  0.6408654  2.7185781
##
## Residual standard error: 16.17698
## Degrees of freedom: 158 total; 157 residual
```

```
#random intercept model
model2 <- lme(autoSxScore ~ 1,
                 data = autoSxDat,
                 method = "REML",
                 random = ~1|id,
                 na.action = "na.exclude")
summary(model2)
```

```
## Linear mixed-effects model fit by REML
##   Data: autoSxDat
##        AIC      BIC    logLik
##   1274.175 1283.343 -634.0873
##
## Random effects:
##  Formula: ~1 | id
##         (Intercept) Residual
## StdDev:    12.85723 9.686717
##
## Fixed effects:  autoSxScore ~ 1
##                Value Std.Error DF  t-value p-value
## (Intercept) 21.76453  1.777319 91 12.24571       0
##
## Standardized Within-Group Residuals:
##        Min         Q1        Med         Q3        Max
## -2.0599674 -0.5201826 -0.1253809  0.5115965  2.9025978
##
## Number of Observations: 158
## Number of Groups: 67
```

```
anova(model1, model2)
```

```
##        Model df      AIC      BIC    logLik   Test  L.Ratio p-value
## model1     1  2 1328.656 1334.769 -662.3281
## model2     2  3 1274.175 1283.343 -634.0873 1 vs 2 56.48161  <.0001
```

```
icc(model2)
```

```
## [1] 0.64
```

```
model3 <- lme(autoSxScore ~ bpiTotalBase + baseToEndOfTx*treatment + endOfTxToFu*treatment,
              data = autoSxDat,
              method = "REML",
              na.action = "na.exclude",
              random = ~1 |id)

summary(model3)
```

```
## Linear mixed-effects model fit by REML
##   Data: autoSxDat
##        AIC      BIC    logLik
##   1267.309 1294.464 -624.6543
##
## Random effects:
##  Formula: ~1 | id
##         (Intercept) Residual
## StdDev:    11.05614 9.730659
##
## Fixed effects:  autoSxScore ~ bpiTotalBase + baseToEndOfTx * treatment + endOfTxToFu *      treatmen
##                              Value Std.Error DF   t-value p-value
## (Intercept)               8.623966  4.926396 87  1.750563  0.0835
## bpiTotalBase              3.218554  0.796832 64  4.039190  0.0001
## baseToEndOfTx            -0.409921  0.247404 87 -1.656892  0.1011
## treatment                -3.919115  3.772757 64 -1.038793  0.3028
## endOfTxToFu              -0.077762  0.100650 87 -0.772592  0.4419
## baseToEndOfTx:treatment   0.399398  0.397516 87  1.004735  0.3178
## treatment:endOfTxToFu     0.061553  0.163593 87  0.376258  0.7076
##  Correlation:
##                          (Intr) bpTtlB bsTEOT trtmnt enOTTF bTEOT:
## bpiTotalBase             -0.872
## baseToEndOfTx            -0.214 -0.001
## treatment                -0.514  0.230  0.280
## endOfTxToFu              -0.202  0.010  0.370  0.255
## baseToEndOfTx:treatment   0.177 -0.050 -0.622 -0.412 -0.231
## treatment:endOfTxToFu     0.146 -0.031 -0.228 -0.377 -0.615  0.353
##
## Standardized Within-Group Residuals:
##        Min         Q1        Med         Q3        Max
## -2.0423729 -0.5320480 -0.1208358  0.5349130  2.5995768
##
## Number of Observations: 158
## Number of Groups: 67
```

```
#cohen's d
#https://stats.stackexchange.com/questions/257985/how-can-i-derive-effect-sizes-in-lme4-and-describe-th
autoSxCohenDat <- autoSxDat %>%
        group_by(treatment, week) %>%
        summarise(meanAutoSx = mean(autoSxScore, na.rm = T)) %>%
        ungroup() %>%
        group_by(week) %>%
        mutate(meanDif = last(meanAutoSx) - first(meanAutoSx)) %>%
        ungroup() %>%
        mutate(autoSxCohenD = purrr::map_dbl(meanDif, nlmeCohenD, model3)) %>%
```

```
        filter(week >= 10) %>%
        select(treatment, week, meanAutoSx, autoSxCohenD)
```

```
## `summarise()` has grouped output by 'treatment'. You can override using the
## `.groups` argument.
```

Generate Cohen's D Table

```
library(rempsyc)
```

```
## Suggested APA citation: Thériault, R. (2022). rempsyc: Convenience functions for psychology
## (R package version 0.1.1) [Computer software]. https://rempsyc.remi-theriault.com
```

```
library(flextable)
```

```
##
## Attaching package: 'flextable'

## The following object is masked from 'package:purrr':
##
##     compose
```

```
library(officer)

secCohTable <- left_join(walkCohenDat, hamdCohenDat, by = c("treatment", "week")) %>%
        left_join(., autoSxCohenDat, by = c("treatment", "week")) %>%
        left_join(., sfGeneralCohenDat, by = c("treatment", "week")) %>%
        mutate(treatment = ifelse(treatment == 0, "Yoga", "CBT")) %>%
        mutate(week = case_when(
                week == 10 ~ "EOT",
                week == 18 ~ "2m FU",
                week == 26 ~ "4m FU",
                week == 34 ~ "6m FU"
        ))

sect_properties <- prop_section(
  page_size = page_size(
    orient = "landscape",
    width = 8.3, height = 11.7
  ))

save_as_docx(secCohTable, path = "secondaryCohTable.docx",
             pr_section = sect_properties)

# standard errors and NA per time point
walkDat %>%
        group_by(treatment, week) %>%
        summarise(walkDistSd = sd(walkDistance, na.rm = T),
                  numNaWalk = sum(!is.na(walkDistance))) %>%
        filter(week == 10 | week == 34)
```

```
## `summarise()` has grouped output by 'treatment'. You can override using the
## `.groups` argument.

## # A tibble: 4 x 4
## # Groups:   treatment [2]
##   treatment  week walkDistSd numNaWalk
##       <dbl> <dbl>      <dbl>     <int>
## 1         0    10      122.         30
## 2         0    34      120.         27
## 3         1    10      114.         18
## 4         1    34       98.9        15
```

```
hamdDat %>%
        group_by(treatment, week) %>%
        summarise(hamdSd = sd(hamdScore, na.rm = T),
                  numNaHamd = sum(!is.na(hamdScore))) %>%
        filter(week == 10 | week == 34)
```

```
## `summarise()` has grouped output by 'treatment'. You can override using the
## `.groups` argument.

## # A tibble: 4 x 4
## # Groups:   treatment [2]
##   treatment  week hamdSd numNaHamd
##       <dbl> <dbl>  <dbl>     <int>
## 1         0    10   8.04        32
## 2         0    34   7.57        28
## 3         1    10   7.31        19
## 4         1    34   8.35        17
```

```
autoSxDat %>%
        group_by(treatment, week) %>%
        summarise(autoSxSd = sd(autoSxScore, na.rm = T),
                  numNaAutoSx = sum(!is.na(autoSxScore))) %>%
        filter(week == 10 | week == 34)
```

```
## `summarise()` has grouped output by 'treatment'. You can override using the
## `.groups` argument.

## # A tibble: 4 x 4
## # Groups:   treatment [2]
##   treatment  week autoSxSd numNaAutoSx
##       <dbl> <dbl>    <dbl>       <int>
## 1         0    10     17.0          30
## 2         0    34     18.4          28
## 3         1    10     15.5          18
## 4         1    34     17.1          17
```

```
sfGeneralDat %>%
        group_by(treatment, week) %>%
        summarise(sfGenSd = sd(generalHealthScore, na.rm = T),
                  numNaSfGen = sum(!is.na(generalHealthScore))) %>%
        filter(week == 10 | week == 34)
```

```
## `summarise()` has grouped output by 'treatment'. You can override using the
## `.groups` argument.

## # A tibble: 4 x 4
## # Groups:   treatment [2]
##   treatment  week sfGenSd numNaSfGen
##       <dbl> <dbl>   <dbl>      <int>
## 1         0    10    21.3         32
## 2         0    34    21.4         28
## 3         1    10    22.3         18
## 4         1    34    22.7         18
```
